# I*t*Oct (I*t*Octyl) – pushing the limits of I*t*Bu: highly hindered electron-rich N-aliphatic N-heterocyclic carbenes†

**DOI:** 10.1039/d3sc01006f

**Published:** 2023-04-18

**Authors:** Md. Mahbubur Rahman, Guangrong Meng, Elwira Bisz, Błażej Dziuk, Roger Lalancette, Roman Szostak, Michal Szostak

**Affiliations:** a Department of Chemistry, Rutgers University 73 Warren Street Newark NJ 07102 USA michal.szostak@rutgers.edu; b Department of Chemistry, Opole University 48 Oleska Street Opole 45-052 Poland; c Department of Chemistry, Wroclaw University of Science and Technology Norwida 4/6 14 Wroclaw 50-373 Poland; d Department of Chemistry, Wroclaw University F. Joliot-Curie 14 Wroclaw 50-383 Poland

## Abstract

I*t*Bu (I*t*Bu = 1,3-di-*tert*-butylimidazol-2-ylidene) represents the most important and most versatile *N*-alkyl N-heterocyclic carbene available in organic synthesis and catalysis. Herein, we report the synthesis, structural characterization and catalytic activity of I*t*Oct (I*t*Octyl), *C*_2_-symmetric, higher homologues of I*t*Bu. The new ligand class, including saturated imidazolin-2-ylidene analogues has been commercialized in collaboration with MilliporeSigma: I*t*Oct, 929 298; SI*t*Oct, 929 492 to enable broad access of the academic and industrial researchers within the field of organic and inorganic synthesis. We demonstrate that replacement of the *t*-Bu side chain with *t*-Oct results in the highest steric volume of *N*-alkyl N-heterocyclic carbenes reported to date, while retaining the electronic properties inherent to N-aliphatic ligands, such as extremely strong σ-donation crucial to the reactivity of *N*-alkyl N-heterocyclic carbenes. An efficient large-scale synthesis of imidazolium I*t*Oct and imidazolinium SI*t*Oct carbene precursors is presented. Coordination chemistry to Au(i), Cu(i), Ag(i) and Pd(ii) as well as beneficial effects on catalysis using Au(i), Cu(i), Ag(i) and Pd(ii) complexes are described. Considering the tremendous importance of I*t*Bu in catalysis, synthesis and metal stabilization, we anticipate that the new class of I*t*Oct ligands will find wide application in pushing the boundaries of new and existing approaches in organic and inorganic synthesis.

## Abstract 2

We report the synthesis, structural characterization and catalytic activity of I*t*Oct (I*t*Octyl), *C*_2_-symmetric, higher homologues of I*t*Bu. Replacement of the *t*-Bu side chain with *t*-Oct results in the highest steric volume of *N*-alkyl N-heterocyclic carbenes reported to date.

## Graphical abstract

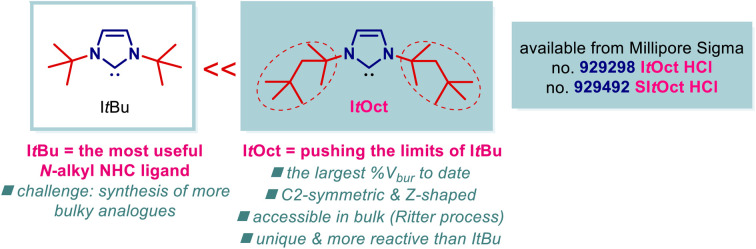

## Introduction

**Fig. 1:**
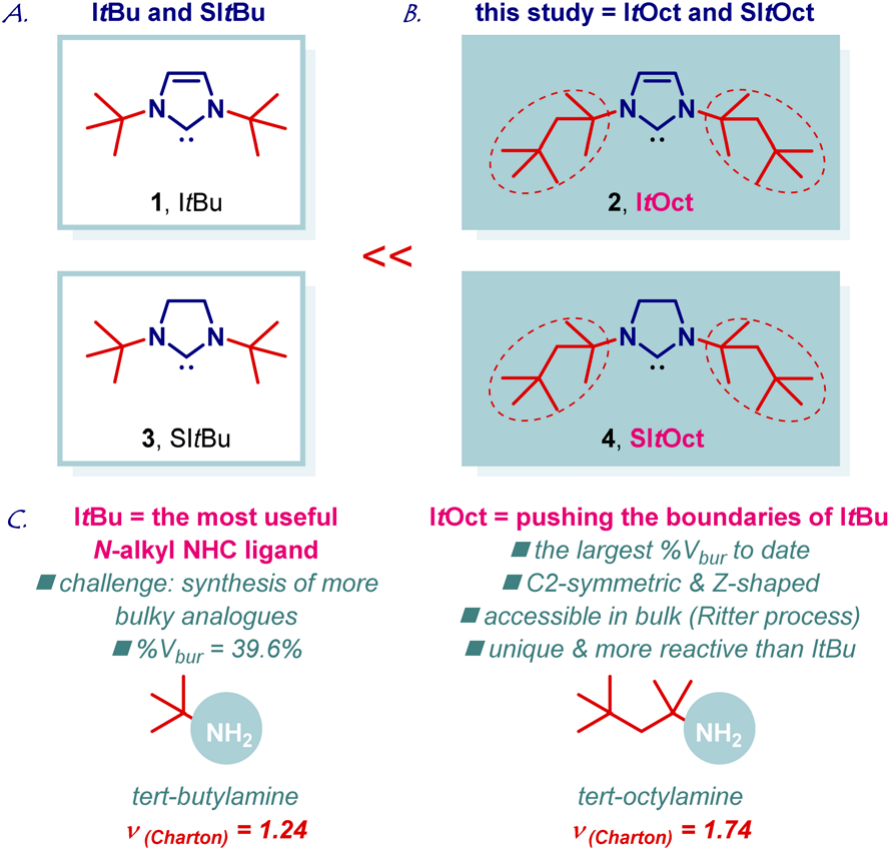
(A–C) Pushing the limits of sterically-demanding *N*-alkyl-heterocyclic carbenes: moving beyond I*t*Bu. I*t*Oct and SI*t*Oct are commercially available from MilliporeSigma: I*t*Oct, 929 298; SI*t*Oct, 929 492.

I*t*Bu (I*t*Bu = 1,3-di-*tert*-butylimidazol-2-ylidene) is the most useful and most general bulky *N*-alkyl N-heterocyclic carbene in organic synthesis and catalysis (Fig. 1A, 1).^1–3^ The importance of I*t*Bu is reflected by the numerous applications in transition-metal-catalysis using an entire palette of metals and transformations. The extraordinary high utility of I*t*Bu stems from the large steric volume (%*V*_bur_ = 39.6%; %*V*_bur_ = %buried volume, [Au(I*t*Bu)Cl]) provided by the bulky *t*-Bu group at the N-wingtip.^4^ Simultaneously, the electron-donating *N*-alkyl groups engender the ligand with strong σ-donation (TEP, 2049 cm^−1^, [Rh(I*t*Bu)(CO)_2_Cl]) and high π-acceptance (^77^Se NMR, *δ*_Se_, 183 ppm, [Se(I*t*Bu)]), which supersede the values observed for N-aromatic NHCs.^5^ Overall, this results in a unique NHC scaffold that has become an indispensable part of the synthetic, organometallic and inorganic toolbox, while providing direct access to novel reactivity, and is now routinely utilized in metal stabilization, reaction screening and optimization. I*t*Bu imidazolium precursor is now commercially available from several suppliers as Cl or BF_4_ salts (CAS: 157197-54-1; CAS: 263163-17-3).^6,7^

**Fig. 2:**
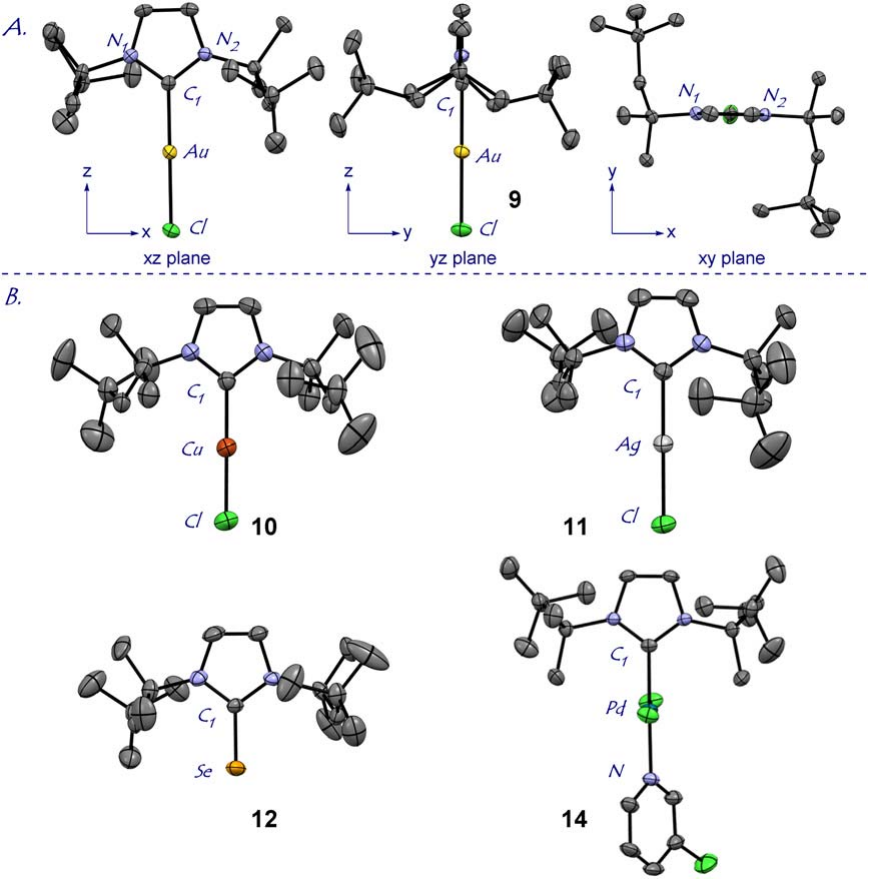
X-ray crystal structures of complexes 9–12, 14. (A) 9: Views along three axes are shown. (B) 10–12, 14. Hydrogen atoms have been omitted for clarity. See ESI† for bond lengths [Å], angles and expanded structures. Crystallographic data have been deposited with the Cambridge Crystallographic Data Center. CCDC 2239373 (9); CCDC 2239374 (10); CCDC 2239375 (11); CCDC 2239376 (12); CCDC 2239377 (14).

**Fig. 3:**
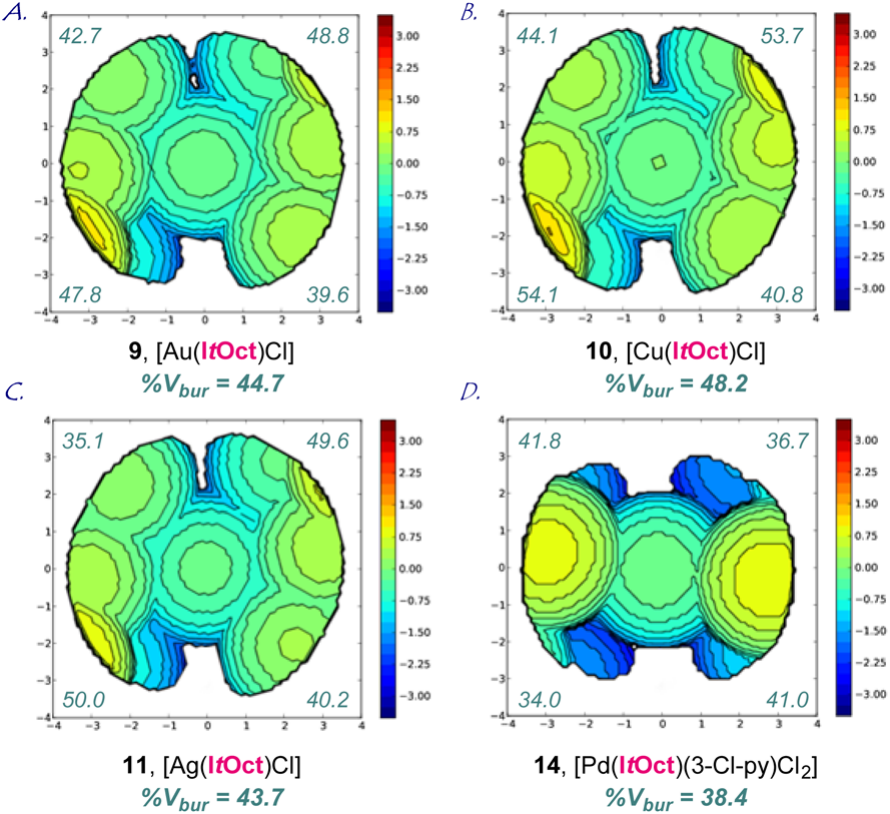
(A–D) Topographical steric maps of [Au(I*t*Oct)Cl] (9), [Cu(I*t*Oct)Cl] (10), [Ag(I*t*Oct)Cl] (11) and [Pd(I*t*Oct)(3-Cl-py)Cl_2_] (14) showing %*V*_bur_ per quadrant. See ESI† for additional details.

**Fig. 4:**
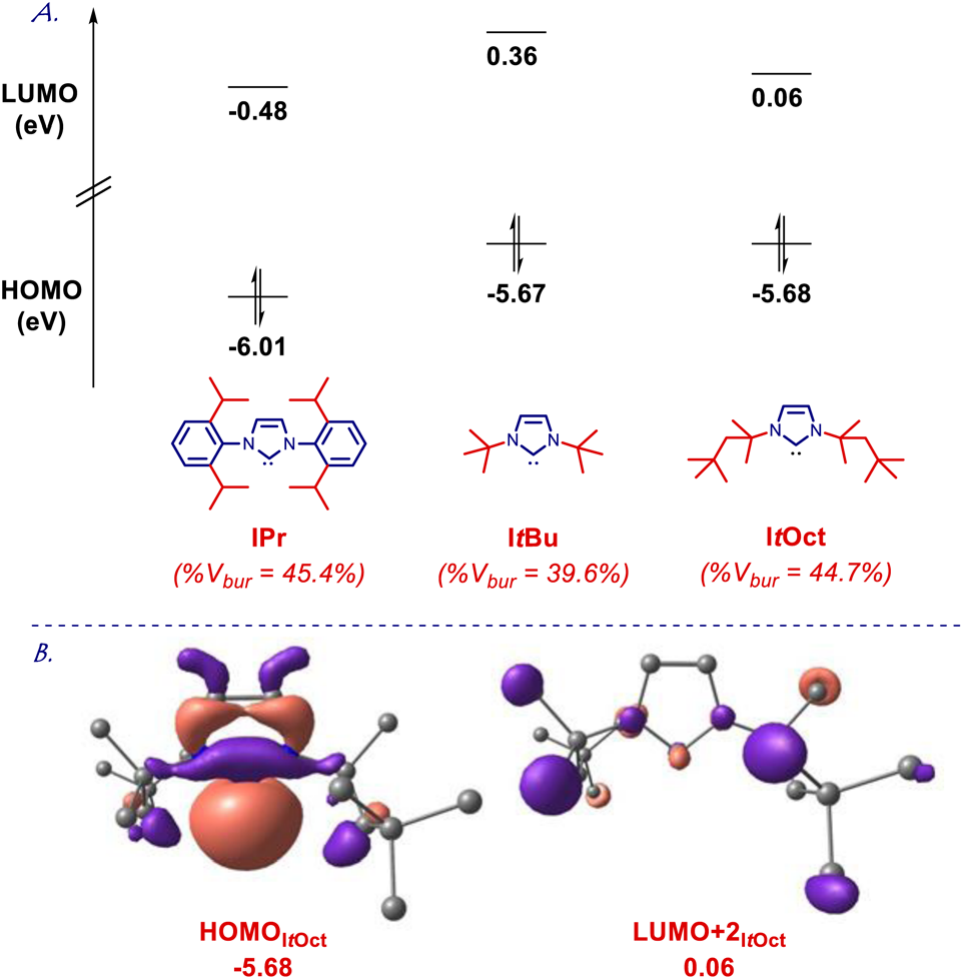
(A) HOMO and LUMO energy levels (eV). (B) HOMO and LUMO+2 (eV) of I*t*Oct calculated at B3LYP 6-311++g(d,p). See ESI.†

As part of our program in transition-metal-catalysis,^8,9^ herein we report the synthesis, structural characterization, and catalytic activity of I*t*Oct (I*t*Octyl) class of ligands, which are *C*_2_-symmetric, higher homologues of I*t*Bu (Fig. 1B, 2). The new ligand class, including saturated imidazolin-2-ylidene analogue, has been commercialized in collaboration with MilliporeSigma: I*t*Oct, 929 298; SI*t*Oct, 929 492, to enable broad access of academic and industrial researchers.^10^ We demonstrate that I*t*Bu to I*t*Oct exchange results in the highest steric volume reported to date for *N*-alkyl N-heterocyclic carbenes (%*V*_bur_ = 44.7%), while retaining electronic properties inherent to *N*-alkyl ligands, such as extremely strong σ-donation and π-acceptance.^4,5^ Notably, the steric volume of I*t*Oct matches the values observed for the archetypal N-aromatic NHC ligands for the first time (IPr, %*V*_bur_ = 45.4%; IMes, %*V*_bur_ = 36.5%, [Au(NHC)Cl]). I*t*Oct features a unique *C*_2_-symmetric and Z-shape scaffold.^11^ The saturated congener, SI*t*Oct (Fig. 1B, 4), is homologous to SI*t*Bu (Fig. 1A, 3). Large scale synthesis, coordination chemistry to Au(i), Cu(i), Ag(i) and Pd(ii), structure and electronic properties of the carbene center as well as beneficial effects on catalysis using Au(i), Cu(i), Ag(i) and Pd(ii) complexes are described. Considering the tremendous importance and utility of I*t*Bu in catalysis, synthesis and metal stabilization^1–3^ we anticipate that the new class of I*t*Oct ligands will find wide application in pushing the limits of *N*-alkyl N-heterocyclic carbenes in organic and inorganic synthesis.

## Results and discussion

The chemistry of *N*-bulky NHC ligands has been studied in a wide array of contexts, including catalysis, coordination chemistry and stabilization of reactive metal centers.^11,12^ We reasoned that an increase in sterics as measured by the Charton parameter (*t*-Bu, *ν* = 1.24; *t*-Oct, *ν* = 1.74, Fig. 1C) would result in an attractive new class of N-aliphatic bulky NHC ligands. As a key element of our design, we recognized that *tert*-octylamine is considerably cheaper than other bulky amines and readily available on kg scale by the Ritter process of the isomeric 2,2,4-trimethylpentenes,^13,14^ which are commercially produced from isobutene feedstock.

**Scheme 1:**
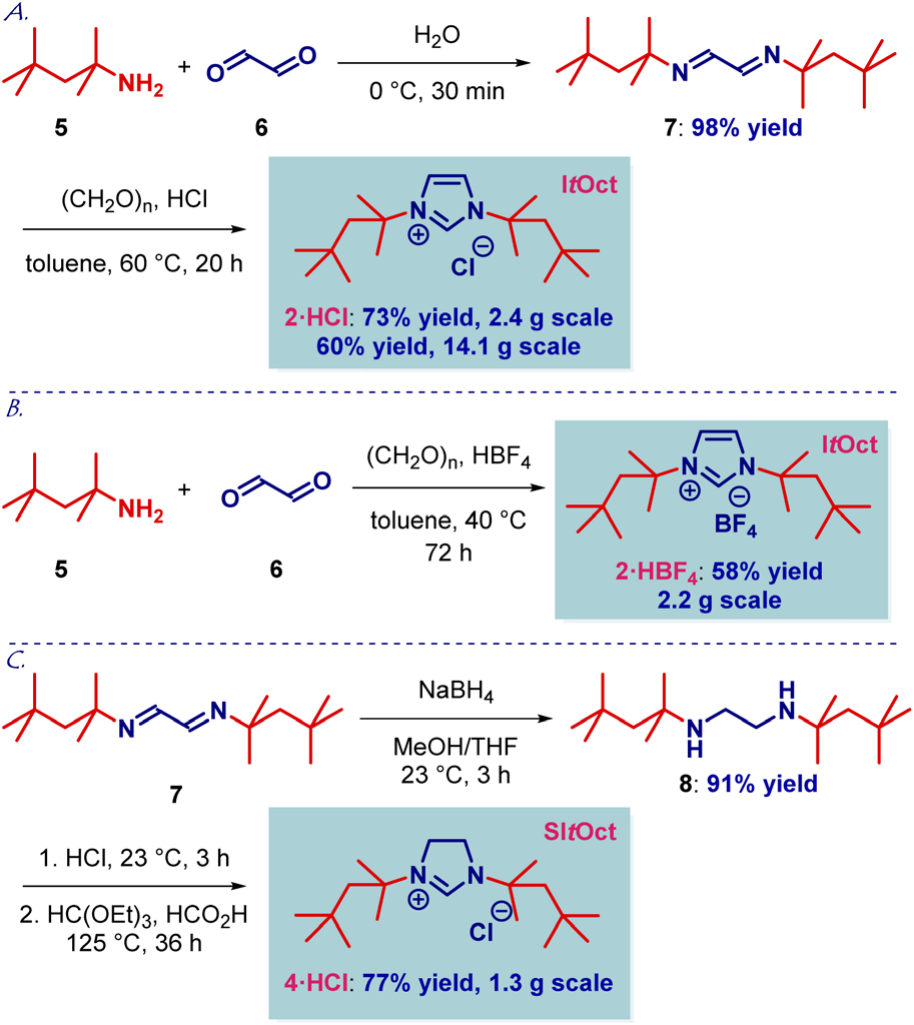
Synthesis of I*t*Oct, SI*t*Oct and precursors. Conditions: (A) 5 (1.0 equiv.), (CHO)_2_ (40%, aq. 0.5 equiv.), H_2_O, 0 °C, then (CH_2_O)_*n*_ (1.0 equiv.), HCl (4.0 M, dioxane, 1.0 equiv.), toluene, 60 °C. (B) 7 (1.0 equiv.), (CH_2_O)_*n*_ (1.0 equiv.), HBF_4_ (48% aq. 1.0 equiv.), toluene, 40 °C; one-step: 5 (1.2 equiv.), (CH_2_O)_*n*_ (1.0 equiv.), HBF_4_ (48% aq. 1.0 equiv.), toluene, 40 °C. (C) 7 (1.0 equiv.), NaBH_4_ (8.0 equiv.), MeOH/THF, 23 °C, then HCl, 23 °C, HC(OEt)_3_ (10.0 equiv.), HCO_2_H, 125 °C.

We initiated our studies by developing a flexible and robust synthesis of I*t*Oct imidazolium precursor using the readily available *tert*-octylamine^13–15^ as the starting material (Scheme 1A). As shown in Scheme 1, the optimized synthesis of I*t*Oct precursor proceeds in cost-effective, chromatography-free, and straightforward manner. Thus, condensation of *tert*-octylamine with glyoxal at room temperature and cyclization of the diimine using a combination of HCl/(CH_2_O)_*n*_ in toluene at 60 °C delivered the desired I*t*Oct as HCl salt after simple filtration, allowing for a routine preparation of gram quantities of the product (step 1: 98% yield, 61 mmol scale; step 2: 73% yield, 10 mmol scale). The synthesis of I*t*Oct as HBF_4_ salt was optimized to proceed in 82% yield (Scheme 1B), while a one-step procedure was developed using the combination of *tert*-octylamine, HBF_4_/(CH_2_O)_*n*_ and glyoxal in 58% yield (see ESI†). The synthesis of SI*t*Oct as HCl salt was accomplished by the reduction of diimine 7 to the diamine using NaBH_4_ in MeOH/THF at room temperature (91% yield, 18 mmol scale) and cyclization to the imidazolinium SI*t*Oct salt using a combination of HC(OEt)_3_/HCO_2_H at 125 °C (77% yield, 5 mmol scale) (Scheme 1C). It should be noted that the synthesis is highly practical and allows for the isolation of I*t*Oct·HCl, I*t*Oct·HBF_4_ and SI*t*Oct·HCl by simple filtration and recrystallization from the reaction mixtures.

**Scheme 2:**
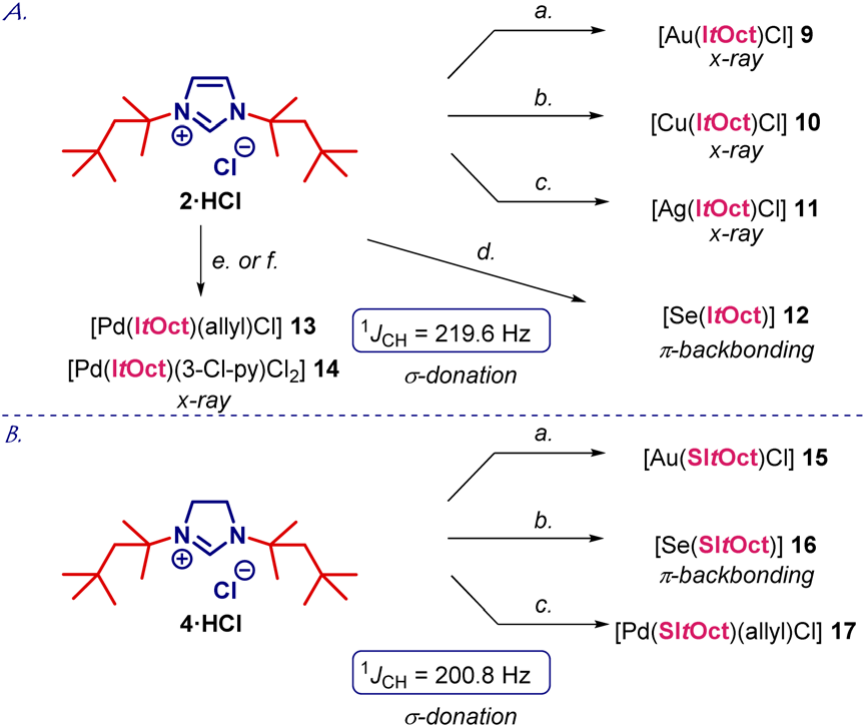
Synthesis of I*t*Oct and SI*t*Oct complexes. Conditions: (A) (a) AuCl·Me_2_S (1.0 equiv.), LiHMDS (1.1 equiv.), THF, 23 °C, 15 h, 84%. (b) CuCl (2.0 equiv.), K_2_CO_3_ (3.0 equiv.), dioxane, 80 °C, 15 h, 76%. (c) Ag_2_O (2.0 equiv.), K_2_CO_3_ (3.0 equiv.), dioxane, 80 °C, 15 h, 80%. (d) Se (2.0 equiv.), K_2_CO_3_ (3.0 equiv.), dioxane, 80 °C, 15 h, 75%. (e) LiHMDS (1.1 equiv.), [Pd(allyl)Cl]_2_ (1.0 equiv.), THF, 23 °C, 15 h, 89%. (f) PdCl_2_ (1.0 equiv.), K_2_CO_3_ (3.0 equiv.), 3-Cl-py, 80 °C, 15 h, 76%. (B) (a) AuCl·Me_2_S (1.0 equiv.), LiHMDS (1.1 equiv), THF, 23 °C, 15 h, 81%. (b) Se (2.0 equiv.), KO*t*-Bu (3.0 equiv.), THF, 23 °C, 15 h, 69%. (c) LiHMDS (1.1 equiv.), [Pd(allyl)Cl]_2_ (1.0 equiv.), THF, 23 °C, 15 h, 74%.

With facile access to I*t*Oct in hand, we next focused on comprehensive evaluation of steric and electronic properties of this novel NHC ligand (Scheme 2A). As shown in Scheme 2, the gold complex [Au(I*t*Oct)Cl] (9) was prepared using LiHMDS/THF, while the method using K_2_CO_3_/acetone gave lower yields.^16^ Moreover, [Ag(I*t*Oct)Cl] (10) and [Cu(I*t*Oct)Cl] (11) were prepared using Ag_2_O/CuCl and K_2_CO_3_ in 1,4-dioxane at 80 °C.^17^ The selenium adduct [Se(I*t*Oct)] (12) was synthesized using selenium/K_2_CO_3_ at 80 °C,^18^ while the Pd(ii) complexes [Pd(I*t*Oct)(allyl)Cl] (13) and [Pd(I*t*Oct)(3-Cl-py)Cl_2_] (14) were prepared from the palladium allyl dimer [{Pd(allyl)(μ-Cl)}_2_] and PdCl_2_/3-Cl-py in the presence of LiHMDS and K_2_CO_3_, respectively.^19,20^ It should be noted that NHC salts (2) and (4) as well as all products 9–14 were found to be stable to air and moisture. Complexes 9–12 and 14 were fully characterized by X-ray crystallography (Fig. 2, 3, and ESI†).^21^

**Fig. 5:**
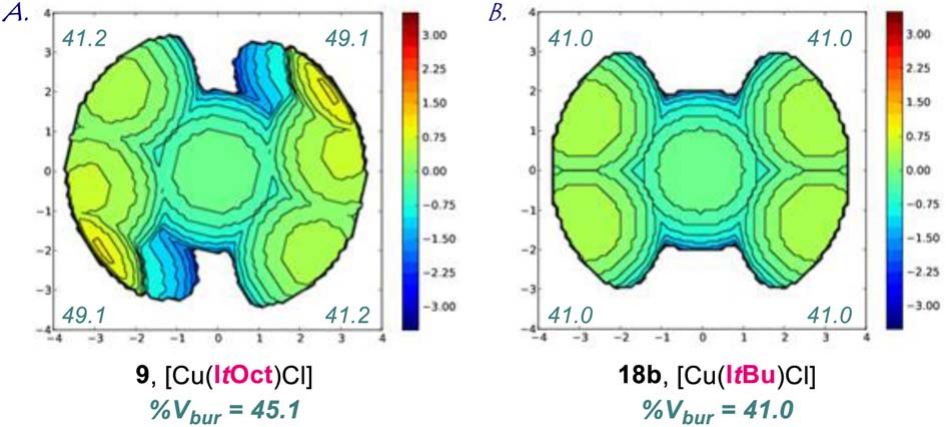
(A and B) Topographical steric maps of [Cu(I*t*Oct)Cl] (9) and [Cu(I*t*Bu)Cl] (18b) showing %*V*_bur_ per quadrant determined at the B3LYP 6-311++g(d,p) level. See ESI† for additional details. Note the steric difference between the I*t*Oct and I*t*Bu ligands.

The X-ray structure of [Au(I*t*Oct)Cl] (9) revealed a unique *C*_2_-symmeric and Z-shape arrangement of *N*-alkyl substituents with a linear (C–Au–Cl, 179.9°; C–Au, 2.007 Å) geometry (Fig. 2A). The % buried volume (%*V*_bur_) of [Au(I*t*Oct)Cl] is 44.7%. Crucially, [Au(I*t*Oct)Cl] represents the most bulky *N*-alkyl NHC ligand reported to date.^4,5^ This value can be compared with the (%*V*_bur_) of 39.6% determined for [Au(I*t*Bu)Cl].^4*b*^ Furthermore, it should be noted that the gem-Me_2_ substitution^22^ of the longer *tert*-Oct side-chain places the metal within the pocket formed by the alkyl side chain. The steric mapping of the metal center^23^ in [Au(I*t*Oct)Cl] is shown in Fig. 3 (see Fig. 5 and ESI† for comparison between [M(I*t*Oct)X] and [M(I*t*Bu)X]). It should be noted that I*t*Oct is large and flexible, while the %*V*_bur_ determined by XRD represent local minima in terms of energies.

Complexes [Ag(I*t*Oct)Cl] (10), [Cu(I*t*Oct)Cl] (11), [Se(I*t*Oct)] (12) and [Pd(I*t*Oct)(3-Cl-py)Cl_2_] (14) were also fully characterized by X-ray crystallography (Fig. 2, 3 and ESI†). The summary of structural parameters is presented in the ESI.† Importantly, the %buried volume (%*V*_bur_) of linear [Ag(I*t*Oct)Cl] (10), [Cu(I*t*Oct)Cl] (11), and [Se(I*t*Oct)] (12) of 43.7%, 48.2% and 44.1%, attests to the immense steric impact of the I*t*Oct substitution. The (%*V*_bur_) of square planar [Pd(I*t*Oct)(3-Cl-py)Cl_2_] (14) is lower of 38.4%, which demonstrates the capacity of the *tert*-octyl side chains to adjust to the steric impact of the metal center (see ESI†).^11^

The selenourea adduct [Se(I*t*Oct)] (12) permits to gauge π-backbonding of I*t*Oct from the ^77^Se NMR spectra.^18^ The *δ*_Se_ value of 216.7 ppm for [Se(I*t*Oct)] (CDCl_3_) indicates that I*t*Oct is more π-accepting than I*t*Bu (*δ*_Se_, 183 ppm, CDCl_3_). Furthermore, ^1^*J*_CH_ coupling constant from the ^13^C satellites of ^1^H NMR spectra of 219.60 Hz for I*t*Oct·HCl (CDCl_3_) gives an accurate indication of σ-donation,^24^ and indicates that this ligand is more strongly donating than *N*-aryl ligands, such as IPr (^1^*J*_CH_ = 223.70 Hz; *cf.*I*t*Bu: ^1^*J*_CH_ = 219.35 Hz).

We also performed the synthesis of representative complexes using the imidazolinium precursor SI*t*Oct (Scheme 2B). The synthesis of [Au(SI*t*Oct)Cl] (15), [Se(SI*t*Oct)] (16) and [Pd(I*t*Oct)(allyl)Cl] (17) proceeded smoothly under the conditions developed for I*t*Oct·HCl (Scheme 2A). The *δ*_Se_ value of 298.2 ppm and the ^1^*J*_CH_ value of 200.80 Hz indicate an increased π-acceptance and σ-donation of the saturated imidazolin-2-ylidene SI*t*Oct, as expected.^4,5^

**Scheme 3:**
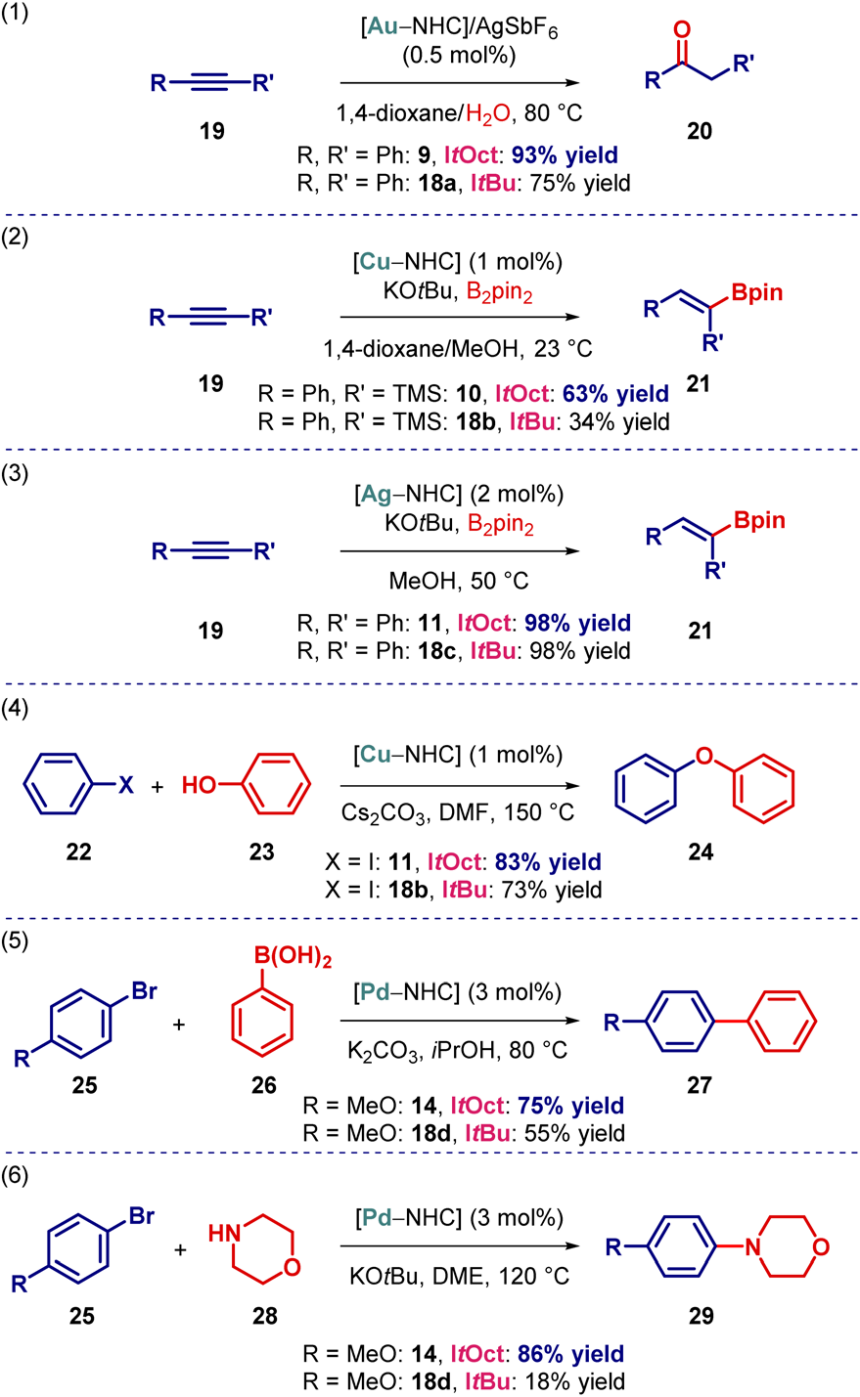
Activity of [I*t*Oct–M] in catalysis. See ESI† for additional details.

From the outset, we proposed that the increased steric bulk of the I*t*Oct ligand would be beneficial on transition-metal-catalysis. To demonstrate the effect of increased steric substitution, we performed several representative reactions in Au(i), Cu(i), Ag(i) and Pd(0) catalysis (Scheme 3). For direct comparison, the corresponding [I*t*Bu–M] complexes were prepared and tested in parallel. As shown, the performance of [Au(I*t*Oct)Cl], [Cu(I*t*Oct)Cl], [Ag(I*t*Oct)Cl], [Pd(I*t*Oct)(3-Cl-py)Cl_2_] in Au(i)-catalyzed hydration,^25^ Cu(i)-catalyzed hydroboration,^26^ Ag(i)-catalyzed hydroboration,^27^ Cu(i)-catalyzed C–O coupling^28^ and Pd(0)-catalyzed C–C and C–N coupling^29^ supersede the analogous [I*t*Bu–M] complexes.

These highly promising preliminary studies provide a strong support for the routine addition of the I*t*Oct class of ligands to the toolbox for reaction screening. Further, it is expected that the I*t*Oct to I*t*Bu replacement will have an even greater effect on stabilizing reactive metal centers by metal shielding.^1–3^ Studies in this direction are currently underway and will be reported in due course.

To gain further insight into the electronic structure of the I*t*Oct class of ligands, we determined HOMO and LUMO energy levels at the B3LYP 6-311++g(d,p) level (Fig. 4 and ESI†). It is well established that computed HOMO and LUMO provide the most accurate estimation of nucleophilicity and electrophilicity of NHC ligands.^5,6^ The HOMO of I*t*Oct (−5.68 eV) is in the same range as I*t*Bu (−5.67 eV), which is much higher than for the archetypal IPr^30^ (−6.01 ev). The HOMO of SI*t*Oct is even higher (−5.50 eV), which can be compared with SI*t*Bu (−5.46 eV). The π-accepting orbital (LUMO+2 due to required symmetry) of I*t*Oct (0.06 eV) and SI*t*Oct (−0.04 eV) are comparable to I*t*Bu (0.36 eV) and SI*t*Bu (0.14 eV), which could be compared with IPr (−0.48 eV). Overall, these results confirm I*t*Oct as strongly σ-nucleophilic and sterically-bulky ligands with electronics matching those of I*t*Bu.

Furthermore, to eliminate impact from steric packing, we have determined the (%*V*_bur_) for the linear [Cu(NHC)Cl] complexes at the B3LYP 6-311++g(d,p) level (NHC = I*t*Oct, I*t*Bu, SI*t*Oct, SI*t*Bu, Fig. 5 and ESI†). The accurate determination of the computed linear geometry obviates effects from crystal packing.^5,6^ [Cu(i)–NHC] complexes were selected to facilitate computations. The %*V*_bur_ of I*t*Oct (45.1%), I*t*Bu (41.0%), SI*t*Oct (47.1%), SI*t*Bu (41.7%) confirm the effects observed in the X-ray analysis and clearly demonstrate the increased steric demand and unique *C*_2_-symmetric Z-shape of I*t*Oct ligands.

**Fig. 6:**
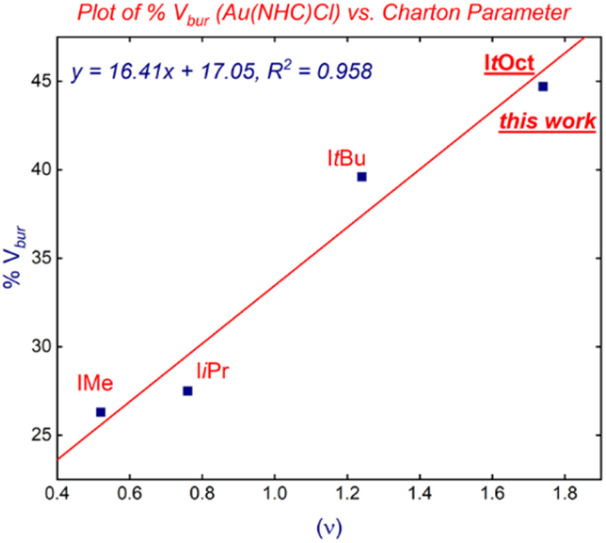
Plot of %*V*_bur_*vs.* Charton parameter in [Au(NHC)Cl] complexes. Note that I*t*Oct is the most sterically-demanding *N*-alkyl-NHC to date.

Interestingly, we found that there is a very good linear correlation between the (%*V*_bur_) and the steric Charton parameter (*ν*)^31^ (Fig. 6) using linear [Au(NHC)Cl] complexes. This finding further establishes I*t*Oct as the most sterically-demanding *N*-alkyl NHC ligands. The present correlation appears to be general and can be used for the future determination of steric impact of *N*-alkyl-substituted NHC ligands. Finally, it should be noted that the expensive yet extremely useful bulky adamantyl (IAd)^32^ is much smaller in volume than I*t*Oct (%*V*_bur_ = 39.8%, IAd *vs.* 44.7%, I*t*Oct).

## Conclusions

In summary, we have reported I*t*Oct (I*t*Octyl) class of ligands that push the limits of I*t*Bu, which is the most useful *N*-alkyl NHC ligand developed to date in various facets of organic and inorganic synthesis. The I*t*Oct class of ligands is characterized by the highest steric volume reported to date for N-aliphatic NHC ligands, while exploiting extremely strong σ-donating electronic properties inherent to *N*-alkyl N-heterocyclic carbenes. The facile preparation of I*t*Oct has been developed using *tert*-octylamine as a product of downstream conversion of feedstock isobutene, which allows for rapid and cost-effective synthesis of I*t*Oct ligands. This route enables routine access and commercial availability. Further, the I*t*Oct class of ligands feature a unique *C*_2_-symmetric Z-shaped steric architecture, making it attractive for future development of strongly σ-donating carbenes. Considering the tremendous importance of N-aliphatic ligands and the commercial availability of the I*t*Oct ligands (MilliporeSigma: I*t*Oct, 929 298; SI*t*Oct, 929 492),^10^ we anticipate that I*t*Oct ligands will find wide application in pushing the boundaries of new and existing approaches in organic and inorganic synthesis.

## Data availability

Experimental procedures, characterization data, crystallographic and computational details are available in the ESI.†

## Author contributions

M. R. developed the ligands and performed the experiments. G. M. was involved in ligand design. E. B. and B. D. provided crystallographic analysis. R. L. was involved in crystallographic studies. R. S. performed DFT computations. M. S. conceived and directed the project and wrote the manuscript.

## Conflicts of interest

The authors declare the following competing financial interests: Rutgers University has filed patents on ligands and precatalysts described in this manuscript (US 63/155492, Mar 2, 2021).

## Supplementary Material

SC-014-D3SC01006F-s001

SC-014-D3SC01006F-s002
