# Supplementary material for: ItOct (ItOctyl) – pushing the limits of ItBu: highly hindered electron-rich N-aliphatic N-heterocyclic carbenes
Source: Chem Sci. 2023 Apr 18;14(19):5141–7. doi: 10.1039/d3sc01006f (PMC10189875; doi:10.1039/d3sc01006f)
Supplement: SC-014-D3SC01006F-s001 [file SC-014-D3SC01006F-s001.pdf]

Electronic Supplementary Material (ESI) for Chemical Science.
This journal is © The Royal Society of Chemistry 2023

Supporting Information

I*t*Oct (I*t*Octyl) – Pushing the Limits of I*t*Bu: Highly Hindered Electron-Rich *N*-Aliphatic *N*-Heterocyclic Carbenes

Md. Mahbubur Rahman,[†] Guangrong Meng,[†] Elwira Bisz,[‡] Błażej Dziuk,[||] Roger Lalancette,[†] Roman Szostak,[§] and Michal Szostak*,[†]

[†]*Department of Chemistry, Rutgers University, 73 Warren Street, Newark, New Jersey 07102, USA*
[‡]*Department of Chemistry, Opole University, 48 Oleska Street, Opole 45-052, Poland*
[||]*Department of Chemistry, Wroclaw University of Science and Technology, Norwida 4/6 14, Wroclaw 50-373, Poland*
[§]*Department of Chemistry, Wroclaw University, F. Joliot-Curie 14, Wroclaw 50-383, Poland*

michal.szostak@rutgers.edu

Table of Contents 1

List of Known Compounds/General Methods 2
Experimental Procedures and Characterization Data 3
Crystallographic Studies 24
Computational Methods 42
References 56
^{1}H and ^{13}C NMR Spectra 57
Cartesian Coordinates with Zero-Point Energies and Thermal Corrections 85

Corresponding Author:
Prof. Dr. M. Szostak
Department of Chemistry, Rutgers University
73 Warren Street, Newark, NJ 07102, United States
E-mail: michal.szostak@rutgers.edu

List of Known Compounds/General Methods

All starting materials reported in the manuscript have been previously described in literature and prepared by the method reported previously unless stated otherwise. All experiments were performed using standard Schlenk techniques under nitrogen or argon unless stated otherwise. All solvents were purchased at the highest commercial grade and used as received or after purification by passing through activated alumina columns or distillation from sodium/benzophenone under nitrogen. All solvents were deoxygenated prior to use. All other chemicals were purchased at the highest commercial grade and used as received. Reaction glassware was oven-dried at 140 °C for at least 24 h or flame-dried prior to use, allowed to cool under vacuum and purged with argon (three cycles). All products were identified using ^{1}H NMR analysis and comparison with authentic samples. GC and/or GC/MS analysis was used for volatile products. All yields refer to yields determined by ^{1}H NMR and/or GC or GC/MS using an internal standard (optimization) and isolated yields (preparative runs) unless stated otherwise. ^{1}H NMR and ^{13}C NMR spectra were recorded in $CDCl_3$ on Bruker spectrometers at 500 (^{1}H NMR) and 125 MHz (^{13}C NMR). All shifts are reported in parts per million (ppm) relative to residual $CHCl_3$ peak (7.26 and 77.36 ppm, ^{1}H NMR and ^{13}C NMR, respectively). All coupling constants (*J*) are reported in hertz (Hz). Abbreviations are: s, singlet; d, doublet; t, triplet; q, quartet; brs, broad singlet. GC-MS chromatography was performed using Agilent HP6890 GC System and Agilent 5973A inert XL EI/CI MSD using helium as the carrier gas at a flow rate of 1 mL/min and an initial oven temperature of 50 °C. The injector temperature was 250 °C. The detector temperature was 250 °C. For runs with the initial oven temperature of 50 °C, temperature was increased with a 10 °C/min ramp after 50 °C hold for 3 min to a final temperature of 220 °C, then hold at 220 °C for 15 min (split less mode of injection, total run time of 22.0 min).High-resolution mass spectra (HRMS) were measured on a 7T Bruker Daltonics FT-MS instrument. All flash chromatography was performed using silica gel, 60 Å, 300 mesh. TLC analysis was carried out on glass plates coated with silica gel 60 F254, 0.2 mm thickness. The plates were visualized using a 254 nm UV lamp or aqueous potassium permanganate.^{1}H NMR and ^{13}C NMR data are given for all compounds in the Supporting Experimental for characterization purposes. ^{1}H NMR, ^{13}C NMR, and HRMS data are given for all new compounds. All products have been previously reported, unless stated otherwise.

Experimental Procedures and Characterization Data

2·HCl, **4·HCl**, **7**, **8**, **9**, **10**, **11**, **12**, **13**, **14**, **15**, **16** and **17** are new compounds. **18a**,[1] **18b**,[2] **18c**,[3] **18d**,[4] **20a**,[5] **20b**,[5] **21a**,[6] **21b**,[7] **24**,[8] **27a**,[9] **27b**,[10] **30a**[11] and **30b**[11] have been previously reported in the literature. Spectroscopic properties matched literature data.

1. General Procedure for the Synthesis of Diimine 7.

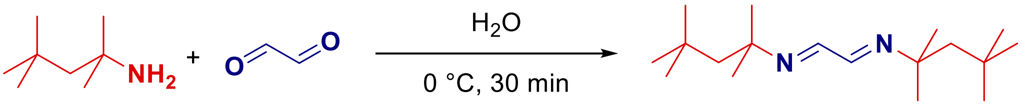

5 **6** **7**

(1*E*,2*E*)-*N*[1],*N*[2]-bis(2,4,4-Trimethylpentan-2-yl)ethane-1,2-diimine (7). A 250 mL round-bottomed flask equipped with a stir bar was charged with glyoxal (**6**) (aq., 40% w/w, 10.0 mL, 87.2 mmol) and water (40.0 mL) and placed in a water bath. *t*-Octylamine (**5**) (20.0 mL, 124.5 mmol) was added dropwise over 60 s with continuous stirring at room temperature. After stirring for 5 min, the reaction mixture was cooled down to 0 °C, and stirred for 30 min at 0 °C. After the indicated time, the resulting precipitate was filtered, washed with an ice-cold water and dried under vacuum to obtain the title product as white solid in 98% yield (17.1 g, 61.0 mmol). (5 mmol scale: 95% yield). Mp = 45–46 °C. ^{1}H NMR (500 MHz, $CDCl_3$) δ 7.90 (s, 2H), 1.66 (s, 4H), 1.25 (s, 12H), 0.89 (s, 18H). ^{13}C NMR (126 MHz, $CDCl_3$) δ 157.93, 62.27, 56.17, 32.41, 31.98, 29.71. IR (KBr, *v*, cm^{-1}): 2956, 2899, 2868, 1621, 1463, 1361, 1355, 1216. HRMS (ESI) m/z: $[M + H]^+$ Calcd for $C_{18}H_{37}N_2$ 281.2951; Found 281.2954.

2. General Procedure for the Synthesis of I*t*Oct·HCl (2·HCl).

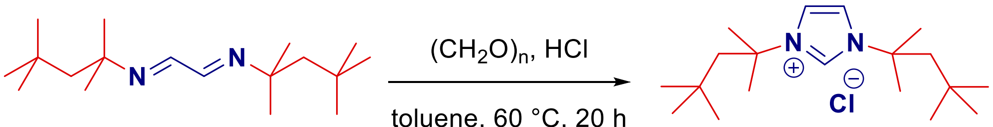

7 **2·HCl**

1,3-bis(2,4,4-Trimethylpentan-2-yl)-1*H*-imidazol-3-ium (I*t*Oct·HCl, 2·HCl). A mixture of paraformaldehyde (305.0 mg, 10.0 mmol) and HCl (4.0 *N*, dioxane, 2.5 mL, 10.0 mmol) was stirred for 1 h at room temperature. After the indicate time, the resulting suspension was added dropwise over 60 s to a solution of diimine (**7**) (2.80 g, 10.0 mmol) in toluene (10.0 mL) in a 100 mL round-bottom flask at room temperature with continuous stirring. The resulting reaction mixture was stirred at 60 °C for 20 h. After the indicated time, diethyl ether

(50 mL) was added and the reaction mixture was stirred for 30 min. The resulting precipitate was filtered, washed with a minimum of diethyl ether and dried under vacuum to obtain the title product as white solid in 73% yield (2.41 g, 7.3 mmol). (71 mmol scale: 60% yield, 14.1 g using EtOAc as a solvent instead of toluene at 40 °C for 48 h). Mp = 246–247 °C. ^{1}H NMR (500 MHz, $CDCl_3$) δ 10.63 (s, 1H), 7.58 (d, J = 0.7 Hz, 2H), 2.04 (s, 4H), 1.78 (s, 12H), 0.78 (s, 18H). ^{13}C NMR (126 MHz, $CDCl_3$) δ 136.68, 119.95, 64.19, 54.44, 31.95, 30.97, 30.72. IR (KBr, ν, cm^{-1}): 3029, 2953, 2905, 2868, 1737, 1538, 1469, 1380, 1217, 1144, 905, 821, 674. HRMS (ESI) m/z: [M – Cl]$^+$ Calcd for $C_{19}H_{37}N_2$ 293.2951; Found 293.2961. *Note:* optimization of the reaction conditions revealed the following points: (1) the reaction proceeds at 23 °C, however increasing the temperature to 40 °C or 60 °C leads to slightly increased yields; (2) the reaction is almost complete after 24 h, however extending the reaction time to 48 h or 72 h, leads to slightly increased yields; (3) EtOAc, toluene and CH_2Cl_2 are all suitable solvents for the reaction; (4) one-pot process is also feasible, however it was not further optimized at this point.

3. General Procedure for the Synthesis of I*t*Oct·HCl (2·HBF_4).

Method A.

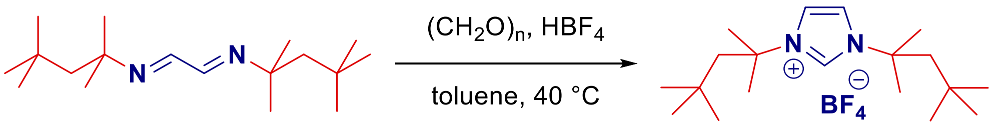

1,3-bis(2,4,4-Trimethylpentan-2-yl)-1*H*-imidazol-3-ium (I*t*Oct·HBF_4, 2·HBF_4). A 100 mL round-bottomed flask equipped with a stir bar was charged with diimine (**7**) (1.4 g, 5.0 mmol), paraformaldehyde (152.0 mg, 5.0 mmol) and toluene (10.0 mL) at room temperature and the resulting mixture was stirred at 40 °C for 1 h. After the indicated time, the reaction mixture was cooled down to room temperature. HBF_4 (aq., 48 % w/w, 0.65 mL, 5.0 mmol) was added with continuous stirring over 60 s, and the resulting mixture was stirred at 40 °C for 15 h. After the indicate time, the reaction mixture was cooled down to room temperature and concentrated. The resulting precipitate was filtered, washed with a minimum of diethyl ether and dried under vacuum to obtain the title product as white solid in 82% yield (1.55 g, 4.08 mmol). Mp = 159–160°C. ^{1}H NMR (500 MHz, $CDCl_3$) δ 9.00 (s, 1H), 7.54 (d, J = 1.5 Hz, 2H), 1.96 (s, 4H), 1.75 (s, 12H), 0.85 (s, 18H). ^{13}C NMR (126 MHz, $CDCl_3$) δ 133.50, 120.61, 64.32, 54.45, 32.04, 30.99, 30.08. ^{19}F NMR (471 MHz, $CDCl_3$) δ -150.64. IR (KBr,

ν, cm^{-1}): 3152, 2952, 1546, 1476, 1219, 1052, 1036, 669, 521. HRMS (ESI) m/z: $[2M – BF_4]^+$ Calcd for $C_{38}H_{74}N_4BF_4$ 673.5944; Found 673.5970.

Note: Alternatively, Method B can also be used for the synthesis of **2·HBF₄**

Method B.

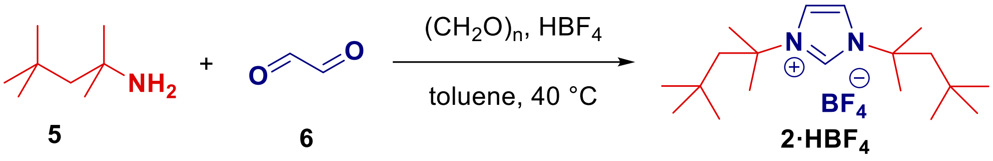

1,3-bis(2,4,4-Trimethylpentan-2-yl)-1*H*-imidazol-3-ium (I*t*Oct·HBF_4, 2·HBF_4). A 100 mL round-bottomed flask equipped with a stir bar was charged with *t*-octylamine (**5**) (2.0 mL, 12.4 mmol), paraformaldehyde (305.0 mg, 10.0 mmol), and toluene (10.0 mL) at room temperature. The resulting mixture was stirred for 30 min at room temperature, cooled down to at 0 °C, and stirred for an addition 1 h at 0 °C. After the indicated time, another portion of *t*-octylamine (**5**) (1.9 mL, 11.8 mmol) was added dropwise over 60 s, followed by the addition of HBF_4 (aq., 48 % w/w, 1.3 mL, 10.0 mmol) dropwise over 60 s. The resulting solution was warmed up at room temperature, glyoxal (**6**) (aq., 40% w/w, 10.0 mL, 87.2 mmol) was added dropwise over 60 s, and the resulting mixture was stirred at 40 °C for 72 h. After the indicate time, the reaction was cooled down to room temperature and concentrated to form a white precipitate. The resulting precipitate was filtered, washed with a minimum of diethyl ether and dried under vacuum to obtain the title product as white solid in 58% yield (2.2 g, 5.8 mmol). ^{1}H NMR (500 MHz, $CDCl_3$) δ 9.00 (s, 1H), 7.54 (d, *J* = 1.5 Hz, 2H), 1.96 (s, 4H), 1.75 (s, 12H), 0.85 (s, 18H). ^{13}C NMR (126 MHz, $CDCl_3$) δ 133.50, 120.61, 64.32, 54.45, 32.04, 30.99, 30.08. ^{19}F NMR (471 MHz, $CDCl_3$) δ -150.64. *Note:* it is important that the reaction is performed for 72 h to obtain satisfactory yields.

4. General Procedure for the Synthesis of Diamine 8.

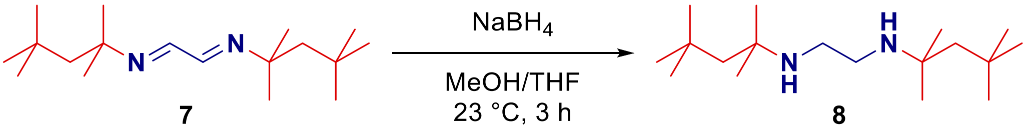

***N*¹,*N*²-bis(2,4,4-Trimethylpentan-2-yl)ethane-1,2-diamine (8).** A 250 mL round-bottomed flask equipped with a stir bar was charged with diimine (**7**) (5.61 g, 20.0 mmol), methanol (20.0 mL) and THF (40.0 mL). $NaBH_4$ (6.05 g, 160.0 mmol) was added slowly at room temperature with vigorous stirring, and the resulting reaction mixture was stirred for 3 h at

room temperature. After the indicated time, the reaction mixture was diluted with diethyl ether (50 mL), washed with saturated NH_4Cl (aq., 50 mL), and the aqueous layer was extracted with diethyl ether (3 x 20 mL). The combined organic layers were dried under vacuum to afford the title product. Yield 91% (5.16 g, 18.1 mmol). (3 mmol scale: 91% yield). White solid. Mp = 30–31 °C. ^{1}H NMR (500 MHz, $CDCl_3$) δ 2.59 (s, 4H), 1.39 (s, 4H), 1.09 (s, 12H), 0.97 (s, 17H). ^{13}C NMR (126 MHz, $CDCl_3$) δ 54.27, 53.88, 42.98, 32.12, 31.96, 29.17. IR (KBr, *v*, cm^{-1}): 3295, 2953, 2906, 1472. HRMS (ESI) m/z: $[M + H]^+$ Calcd for $C_{18}H_{41}N_2$ 285.3264; Found 285.3265.

5. General Procedure for the Synthesis of SI*t*Oct·HCl (4·HCl).

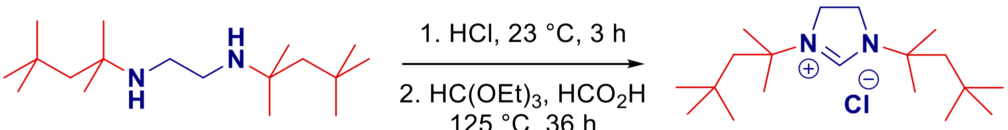

8 4·HCl

***N*¹,*N*²-bis(2,4,4-Trimethylpentan-2-yl)ethane-1,2-diamine·HCl (8·HCl).** A mixture of diamine **8** (4.27 g, 15.0 mmol) and HCl (aq., 1.0 *N*, 50.0 mL) was stirred at room temperature for 3 h. After the indicated time, the resulting precipitate was filtered, washed with a minimum of diethyl ether and dried under vacuum to afford the title product. Yield 96% (5.14 g, 14.4 mmol). White solid. **1,3-bis(2,4,4-Trimethylpentan-2-yl)-4,5-dihydro-1*H*-imidazol-3-ium (SI*t*Oct·HCl, 4·HCl).** A 100 mL round-bottomed flask equipped with a stir bar was charged with triethyl orthoformate (8.3 mL, 50.0 mmol), diamine·HCl salt **8·HCl** (1.79 g, 5.0 mmol) and formic acid (2 drops) at room temperature. The resulting reaction mixture was stirred at 125 °C for 36 h. After the indicate time, the reaction was cooled down to room temperature and concentrated to form a white precipitate. The resulting precipitate was filtered, washed with a minimum of diethyl ether and dried under vacuum to obtain the title product as white solid in 77% yield (1.28 g, mmol, 3.9 mmol). (1.75 mmol scale: 77% yield). Mp = 210–211 °C. ^{1}H NMR (500 MHz, $CDCl_3$) δ 8.81 (s, 1H), 4.00 (s, 4H), 1.63 (s, 4H), 1.54 (s, 12H), 0.91 (s, 18H). ^{13}C NMR (126 MHz, $CDCl_3$) δ 155.15, 61.08, 51.27, 45.76, 31.80, 31.40, 28.82. IR (KBr, *v*, cm^{-1}): 3451, 3389, 2947, 2905, 2870, 1622, 1464, 1292, 1211, 1135, 503. HRMS (ESI) m/z: $[M – Cl]^+$ Calcd for $C_{19}H_{39}N_2$ 295.3108; Found 295.3117.

6. General Procedure for the Synthesis of [Au(I*t*Oct)Cl].

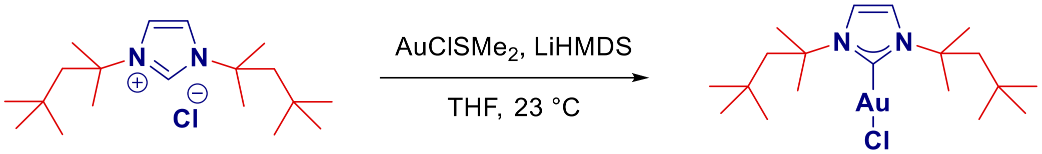

2·HCl **9**

[Au(I*t*Oct)Cl] (9). An oven-dried flask equipped with a stir bar was charged with I*t*Oct·HCl (39.5 mg, 0.12 mmol, 1.2 equiv), placed under a positive pressure of argon and subjected to three evacuation/backfilling cycles under high vacuum. THF (0.5 mL) and LiHMDS (1.0 M in THF, 0.11 mL, 0.11 mmol, 1.1 equiv) were added and the reaction mixture was stirred at room temperature for 2 h. After the indicated time, a solution of $AuClSMe_2$ (29.5 mg, 0.1 mmol, 1.0 equiv) in THF (0.5 mL) was added dropwise, and the resulting mixture was stirred at room temperature for 15 h. After the indicated time, the reaction mixture was diluted with CH_2Cl_2 (10 mL) and filtered. The solution was collected and concentrated. The title product was obtained by trituration from hexanes as a white solid. Yield 84% (43.9 mg). Mp = 219–220 °C. ^{1}H NMR (500 MHz, $CDCl_3$) δ 7.10 (s, 2H), 2.64 (s, 4H), 1.85 (s, 12H), 0.82 (s, 18H). ^{13}C NMR (126 MHz, $CDCl_3$) δ 170.34, 117.39, 62.57, 54.95, 32.36, 32.28, 31.49. IR (KBr, *v*, cm^{-1}): 3166, 2948, 2868, 1739, 1568, 1472, 1407, 1390, 1363, 1215, 1176, 730, 701, 636. HRMS (ESI) m/z: $[2M + K]^+$ Calcd for $C_{38}H_{72}N_4Cl_2Au_2K$ 1087.4097; Found 1087.4168.

7. General Procedure for the Synthesis of [Cu(I*t*Oct)Cl].

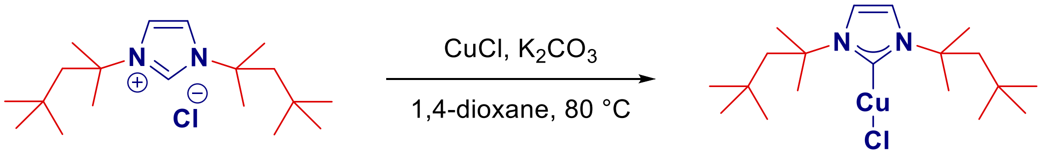

2·HCl **10**

[Cu(I*t*Oct)Cl] (10). An oven-dried flask equipped with a stir bar was charged with I*t*Oct·HCl (32.9 mg, 0.1 mmol, 1.0 equiv), CuCl (19.8 mg, 0.2 mmol, 2.0 equiv) and K_2CO_3 (41.4 mg, 0.3 mmol, 3.0 equiv), placed under a positive pressure of argon, and subjected to three evacuation/backfilling cycles under high vacuum. 1,4-Dioxane (1.0 mL) was added, and the reaction mixture was stirred at 80 °C for 15 h. After the indicated time, the reaction mixture was diluted with CH_2Cl_2 (10 mL) and filtered. The solution was collected and concentrated. The title product was obtained by trituration from hexanes as a white solid. Yield 76% (29.8 mg). Mp = 194–195 °C. ^{1}H NMR (500 MHz, $CDCl_3$) δ 7.04 (s, 2H), 2.33 (s, 4H), 1.80 (s, 12H), 0.82 (s, 18H). ^{13}C NMR (126 MHz, $CDCl_3$) δ 175.12, 117.40, 61.34, 56.52, 32.41,

32.29, 31.43. IR (KBr, *v*, cm^{-1}): 3159, 2949, 2869, 1470, 1400, 1217, 1174, 730, 698, 638. HRMS (ESI) m/z: $[2M – Cl]^+$ Calcd for $C_{38}H_{72}N_4ClCu_2$ 747.4013; Found 747.4021.

8. General Procedure for the Synthesis of [Ag(I*t*Oct)Cl].

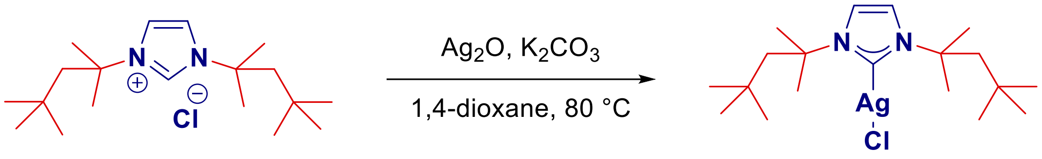

2·HCl 11

[Ag(I*t*Oct)Cl] (11). An oven-dried flask equipped with a stir bar was charged with I*t*Oct·HCl (32.9 mg, 0.1 mmol, 1.0 equiv), Ag_2O (46.3 mg, 0.2 mmol, 2.0 equiv) and K_2CO_3 (41.4 mg, 0.3 mmol, 3.0 equiv), placed under a positive pressure of argon, and subjected to three evacuation/backfilling cycles under high vacuum. 1,4-Dioxane (1.0 mL) was added, and the reaction mixture was stirred at 80 °C for 15 h. After the indicated time, the reaction mixture was diluted with CH_2Cl_2 (10 mL) and filtered. The solution was collected and concentrated. The title product was obtained by trituration from hexanes as a white solid. Yield 80% (35.0 mg). Mp = 169–170 °C. ^{1}H NMR (500 MHz, $CDCl_3$) δ 7.11 (d, J = 1.7 Hz, 2H), 2.26 (s, 4H), 1.76 (s, 12H), 0.79 (s, 18H). ^{13}C NMR (126 MHz, $CDCl_3$) δ 178.03 (dd, J^{Ag} = 257.1, 18.5 Hz), 117.85 (d, J^{Ag} = 8.0 Hz), 61.43, 56.34, 32.33, 32.27, 31.46. IR (KBr, *v*, cm^{-1}): 3152, 2949, 1614, 1471, 1391, 1366, 1216, 1173, 1134, 732, 691, 635. HRMS (ESI) m/z: $[2M – Cl]^+$ Calcd for $C_{38}H_{72}N_4ClAg_2$ 835.3535; Found 835.3559.

9. General Procedure for the Synthesis of [Se(I*t*Oct)].

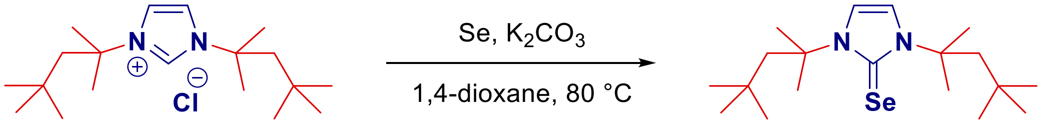

2·HCl 12

[Se(I*t*Oct)] (12). An oven-dried flask equipped with a stir bar was charged with I*t*Oct·HCl (32.9 mg, 0.1 mmol, 1.0 equiv), selenium (15.8 mg, 0.2 mmol, 2.0 equiv), and K_2CO_3 (41.4 mg, 0.3 mmol, 3.0 equiv), placed under a positive pressure of argon, and subjected to three evacuation/backfilling cycles under high vacuum. 1,4-Dioxane (1.0 mL) was added, and the reaction mixture was stirred at 80 °C for 15 h. After the indicated time, the reaction mixture was diluted with CH_2Cl_2 (10 mL) and filtered. The solution was collected and concentrated. The title product was obtained by trituration from hexanes as a white solid. Yield 75% (28.0 mg). Mp = 131–132 °C. ^{1}H NMR (500 MHz, $CDCl_3$) δ 7.01 (s, 2H), 2.77 (s, 4H), 1.85 (s,

12H), 0.86 (s, 18H). ^{13}C NMR (126 MHz, $CDCl_3$) δ 151.95, 116.68, 64.65, 47.44, 32.07, 31.48, 30.87. ^{77}Se NMR (95 MHz, $CDCl_3$) δ 216.72. IR (KBr, *v*, cm^{-1}): 2948, 2900, 2867, 1740, 1476, 1365, 1341, 1209, 1176, 1131, 1043. HRMS (ESI) m/z: $[M + Na]^+$ Calcd for $C_{19}H_{36}N_2SeNa$ 395.1937; Found 395.1949.

10. General Procedure for the Synthesis of [Pd(I*t*Oct)(allyl)Cl].

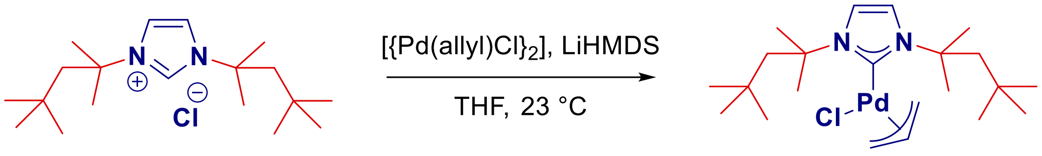

[Pd(I*t*Oct)(allyl)Cl] (13). An oven-dried flask equipped with a stir bar was charged with I*t*Oct·HCl (39.5 mg, 0.12 mmol, 1.2 equiv), placed under a positive pressure of argon and subjected to three evacuation/backfilling cycles under high vacuum. THF (0.5 mL) and LiHMDS (1.0 M in THF, 0.11 mL, 0.11 mmol, 1.1 equiv) were added and the resulting reaction mixture was stirred at room temperature for 2 h. After the indicated time, $[\{Pd(allyl)Cl\}_2]$ (29.5 mg, 0.1 mmol, 1.0 equiv) in THF (0.5 mL) was added and the reaction mixture was stirred at room temperature for 15 h. After the indicated time, the reaction mixture was diluted with CH_2Cl_2 (10 mL) and filtered. The solution was collected and concentrated. The title product was obtained by trituration from hexanes as a yellow solid. Yield 72% (34.1 mg). Mp = 152–153 °C. ^{1}H NMR (500 MHz, $CDCl_3$) δ 7.11 (dd, J = 9.5, 2.1 Hz, 2H), 5.26 – 5.17 (m, 1H), 4.10 (dd, J = 7.3, 2.2 Hz, 1H), 3.39 (d, J = 6.5 Hz, 1H), 3.25 (d, J = 13.2 Hz, 1H), 2.26 (d, J = 11.7 Hz, 1H), 2.13 – 2.05 (m, 4H), 1.99 (s, 2H), 1.92 – 1.85 (m, 7H), 1.69 (s, 3H), 0.83 (d, J = 10.8 Hz, 18H). ^{13}C NMR (126 MHz, $CDCl_3$) δ 177.52, 119.36, 119.03, 112.42, 70.06, 62.63, 62.58, 55.72, 55.16, 52.46, 32.68, 32.62, 32.50, 32.12, 32.08, 32.06, 31.56, 31.47. IR (KBr, *v*, cm^{-1}): 2950, 1739, 1459, 1367, 1210, 1169. HRMS (ESI) m/z: $[2M – Cl]^+$ Calcd for $C_{44}H_{82}N_4ClPd_2$ 915.4303; Found 915.4298.

11. General Procedure for the Synthesis of [Pd(I*t*Oct)(3-Cl-py)Cl_2].

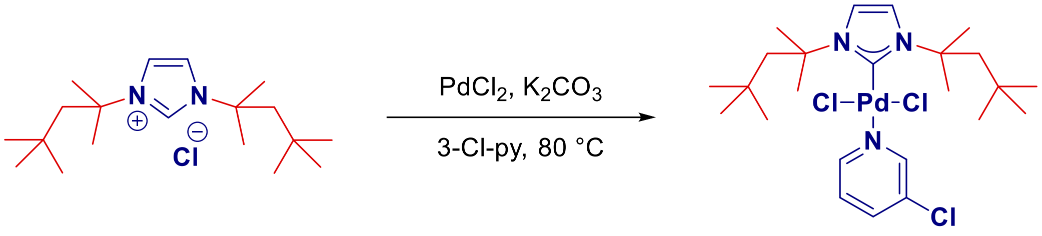

[Pd(I*t*Oct)(3-Cl-py)Cl_2] (14). An oven-dried flask equipped with a stir bar was charged with I*t*Oct·HCl (32.9 mg, 0.1 mmol, 1.0 equiv), $PdCl_2$ (17.7 mg, 0.1 mmol, 1.0 equiv) and K_2CO_3 (41.4 mg, 0.3 mmol, 3.0 equiv), placed under a positive pressure of argon and subjected to three evacuation/backfilling cycles under high vacuum. 3-Chloropyridine (0.5 mL) was added, and the reaction mixture was stirred at 80 °C for 15 h. After the indicated time, the reaction mixture was diluted with CH_2Cl_2 (10 mL) and filtered. The solution was collected and concentrated. The title product was obtained by trituration from hexanes as a yellow solid. Yield 56% (37.9 mg). Mp = 199–200 °C. ^{1}H NMR (500 MHz, $CDCl_3$) δ 9.01 (d, *J* = 2.2 Hz, 1H), 8.94 (dd, *J* = 5.4, 1.0 Hz, 1H), 7.74 (ddd, *J* = 8.2, 2.1, 1.3 Hz, 1H), 7.30 (dd, *J* = 8.2, 5.5 Hz, 1H), 7.22 (s, 2H), 2.46 (s, 12H), 2.11 (s, 4H), 0.97 (s, 18H). ^{13}C NMR (126 MHz, $CDCl_3$) δ 151.04, 150.09, 141.33, 138.12, 132.94, 125.19, 121.04, 63.89, 55.88, 33.22, 32.21, 31.62. IR (KBr, *v*, cm^{-1}): 3136, 2957, 1592, 1461, 1368, 1116, 1048, 797, 744, 688, 647. HRMS (ESI) m/z: $[2M - 2(Cl + NC_5H_4Cl)]^+$ Calcd for $C_{38}H_{72}N_4Cl_2Pd_2$ 868.3194; Found 868.3179.

12. General Procedure for the Synthesis of [Au(SI*t*Oct)Cl].

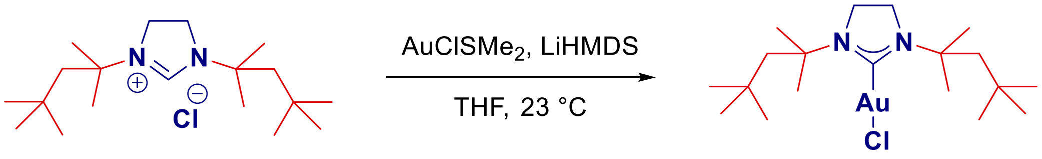

[Au(SI*t*Oct)Cl] (15). An oven-dried flask equipped with a stir bar was charged with SI*t*Oct·HCl (39.7 mg, 0.12 mmol, 1.2 equiv), placed under a positive pressure of argon and subjected to three evacuation/backfilling cycles under high vacuum. THF (0.5 mL) and LiHMDS (1.0 M in THF, 0.11 mL, 0.11 mmol, 1.1 equiv) were added and the reaction mixture was stirred at room temperature for 2 h. After the indicated time, a solution of $AuClSMe_2$ (29.5 mg, 0.1 mmol, 1.0 equiv) in THF (0.5 mL) was added dropwise, and the resulting mixture was stirred at room temperature for 15 h. After the indicated time, the

reaction mixture was diluted with CH_2Cl_2 (10 mL) and filtered. The solution was collected and concentrated. The title product was obtained by trituration from hexanes as a white solid. Yield 81% (42.8 mg). ^{1}H NMR (500 MHz, $CDCl_3$) δ 3.64 (s, 4H), 2.48 (s, 4H), 1.62 (s, 12H), 1.01 (s, 18H). ^{13}C NMR (126 MHz, $CDCl_3$) δ 193.23, 60.39, 55.22, 46.69, 32.33, 32.14, 31.47. Mp = 154–155 °C. IR (KBr, *v*, cm^{-1}): 2948, 2901, 1470, 1436, 1422, 1267, 1212, 1128, 613, 600. HRMS (ESI) m/z: [M + (MeCN) – Cl]$^+$ Calcd for $C_{21}H_{41}N_3Au$ 532.2961; Found 532.2980.

13. General Procedure for the Synthesis of [(SI*t*Oct)Se].

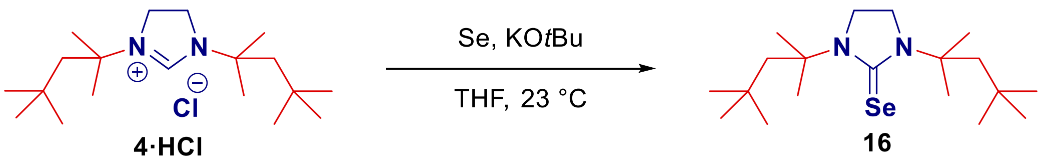

[(SI*t*Oct)Se] (16). An oven-dried flask equipped with a stir bar was charged with SI*t*Oct·HCl (33.1 mg, 0.1 mmol, 1.0 equiv), selenium (15.8 mg, 0.2 mmol, 2.0 equiv), and KO^{t}Bu (33.7 mg, 0.3 mmol, 3.0 equiv), placed under a positive pressure of argon, and subjected to three evacuation/backfilling cycles under high vacuum. THF (1.0 mL) was added, and the reaction mixture was stirred at room temperature for 15 h. After the indicated time, the reaction mixture was diluted with CH_2Cl_2 (10 mL) and filtered. The solution was collected and concentrated. The title product was obtained by trituration from hexanes as a white solid. Yield 69% (25.6 mg). ^{1}H NMR (500 MHz, $CDCl_3$) δ 3.53 (s, 4H), 2.59 (s, 4H), 1.64 (s, 12H), 1.03 (s, 18H). ^{13}C NMR (126 MHz, $CDCl_3$) δ 178.62, 62.24, 48.60, 45.99, 32.00, 31.90, 30.38. ^{77}Se NMR (95 MHz, $CDCl_3$) δ 298.15. Mp = 113–114 °C. IR (KBr, *v*, cm^{-1}): 2950, 2899, 2866, 1471, 1393, 1297, 1265, 1210, 1126. HRMS (ESI) m/z: [2M + Na]$^+$ Calcd for $C_{38}H_{76}N_4Se_2Na$ 771.4300; Found 771.4326.

14. General Procedure for the Synthesis of [Pd(SI*t*Oct)(allyl)Cl].

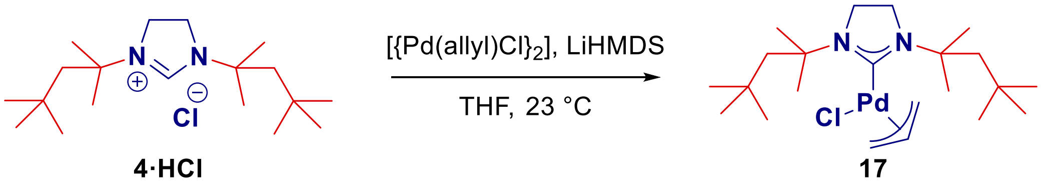

[Pd(SI*t*Oct)(allyl)Cl] (17). An oven-dried flask equipped with a stir bar was charged with SI*t*Oct·HCl (39.7 mg, 0.12 mmol, 1.2 equiv), placed under a positive pressure of argon and subjected to three evacuation/backfilling cycles under high vacuum. THF (0.5 mL) and

LiHMDS (1.0 M in THF, 0.11 mL, 0.11 mmol, 1.1 equiv) were added and the resulting reaction mixture was stirred at room temperature for 2 h. After the indicated time, [{Pd(allyl)Cl}$_2$] (29.5 mg, 0.1 mmol, 1.0 equiv) in THF (0.5 mL) was added and the reaction mixture was stirred at room temperature for 15 h. After the indicated time, the reaction mixture was diluted with CH_2Cl_2 (10 mL) and filtered. The solution was collected and concentrated. The title product was obtained by trituration from hexanes as a yellow solid. Yield 74% (35.5 mg). Mp = 145–146 °C. ^{1}H NMR (500 MHz, $CDCl_3$) δ 5.24 – 5.15 (m, 1H), 4.08 (dd, *J* = 7.1, 1.1 Hz, 1H), 3.65 – 3.52 (m, 4H), 3.40 (d, *J* = 6.3 Hz, 1H), 3.25 (d, *J* = 13.3 Hz, 1H), 2.22 (d, *J* = 11.6 Hz, 1H), 2.01 (s, 3H), 1.83 – 1.71 (m, 9H), 1.58 (s, 4H), 1.01 (d, *J* = 13.7 Hz, 18H). ^{13}C NMR (126 MHz, $CDCl_3$) δ 206.92, 112.74, 69.82, 60.84, 60.68, 53.21, 52.88, 51.12, 46.16, 45.92, 31.97, 31.94, 31.91, 31.55, 31.10, 30.99. IR (KBr, *ν*, cm^{-1}): 2949, 1736, 1421, 1362, 1274, 1209. HRMS (ESI) m/z: $[2M – Cl]^+$ Calcd for $C_{44}H_{86}N_4ClPd_2$ 919.4616; Found 919.4609.

Catalytic Activity of [M–I*t*Oct] Complexes

Catalytic reactivity of [M–I*t*Oct] was benchmarked against [M–I*t*Bu] complexes. **[Au(I*t*Bu)Cl] (18a)**, **[Cu(I*t*Bu)Cl] (18b)**, **[Ag(I*t*Bu)Cl] (18c)**, **[Pd(I*t*Bu)(3-Cl-py)Cl$_2$] (18d)** were prepared according to the known procedures and tested in parallel against the corresponding [M–I*t*Oct] complexes.

15. General Procedure for [Au–NHC]-Catalyzed Hydration of Alkynes.

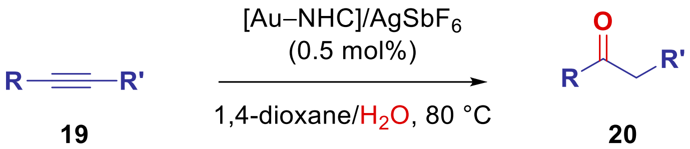

General procedure. An oven-dried vial equipped with a stir bar was charged with an alkyne substrate (1.0 mmol), [Au–NHC] catalyst (0.5 mol%), $AgSbF_6$ (covering the tip of a spatula), 1,4-dioxane (1.0 mL) and stirred for 1 min. Distilled water (0.1 mL) was added and the reaction mixture was stirred at 80 °C for 15 h. After the indicated time, the reaction mixture was diluted with water (5 mL), extracted with CH_2Cl_2 (3 x 5 mL), dried and concentrated. The sample was analyzed by ^{1}HNMR ($CDCl_3$, 500 MHz) and GC-MS to obtain conversion, selectivity and yield using internal standard and comparison with authentic samples. Purification by chromatography on silica gel (EtOAc/hexanes) afforded the title product.

Table SI-1. *Comparative Reactivity of [(ItOct)AuCl] in Hydration of Alkynes.*

Entry	Alkyne	Catalyst	Yield (%)
1	Diphenylacetylene (**19a**)	[(I*t*Oct)AuCl] (**9**)	93
2	Diphenylacetylene (**19a**)	[(I*t*Bu)AuCl] (**18a**)	75
3	Octyne-1 (**19b**)	[(I*t*Oct)AuCl] (**9**)	98
4	Octyne-1 (**19b**)	[(I*t*Bu)AuCl] (**18a**)	72

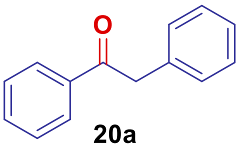

20a

Benzyl phenyl ketone (20a). White solid. ^{1}H NMR (500 MHz, $CDCl_3$) δ 7.97 – 7.89 (m, 2H), 7.49 – 7.44 (m, 1H), 7.37 (t, J = 7.7 Hz, 2H), 7.27 – 7.22 (m, 2H), 7.20 – 7.15 (m, 3H), 4.20 (s, 2H). ^{13}C NMR (126 MHz, $CDCl_3$) δ 197.93, 136.93, 134.86, 133.48, 129.79, 128.99, 128.96, 128.93, 127.21, 45.82.

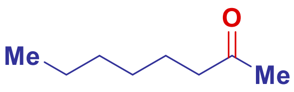

20b

Octanone-2 (20b). Colorless oil. ^{1}H NMR (500 MHz, $CDCl_3$) δ 2.41 (t, *J* = 7.5 Hz, 2H), 2.13 (s, 3H), 1.58 – 1.53 (m, 2H), 1.32 – 1.25 (m, 8H), 0.87 (t, *J* = 6.6 Hz, 3H). ^{13}C NMR (126 MHz, $CDCl_3$) δ 209.73, 44.17, 31.93, 30.18, 29.19, 24.17, 22.83, 14.36.

16. General Procedure for [Cu–NHC]-Catalyzed Hydroboration of Alkynes.

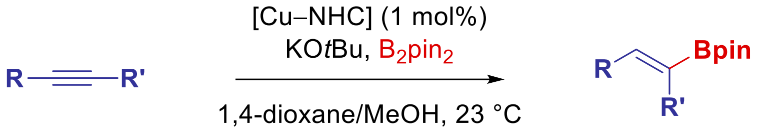

19 21

<u>*General procedure.*</u> An oven-dried vial equipped with a stir bar was charged with an alkyne substrate (1.0 mmol, 1.0 equiv), bis(pinacolato)diboron (304.7 mg, 1.2 mmol, 1.2 equiv), KO*t*Bu (11.2 mg, 0.1 mmol, 0.1 equiv), 1,4-dioxane (2.4 mL), MeOH (0.2 mL), [Cu–NHC]-catalyst (1.0 mol%) and stirred at room temperature for 15 h. After the indicate time, the solvent was removed under reduce pressure. The sample was analyzed by ^{1}HNMR ($CDCl_3$, 500 MHz) and GC-MS to obtain conversion, selectivity and yield using internal standard and comparison with authentic samples. Purification by chromatography on silica gel (EtOAc/hexanes) afforded the title product.

Table SI-2. *Comparative Reactivity of [(ItOct)CuCl] in Hydroboration of Alkynes.*

Entry	Alkyne	Catalyst	Yield (%)
1	Diphenylacetylene (**19a**)	[(I*t*Oct)CuCl] (**10**)	65
2	Diphenylacetylene (**19a**)	[(I*t*Bu)CuCl] (**18b**)	48
3	1-Phenyl-2-trimethylsilylacetylene (**19c**)	[(I*t*Oct)CuCl] (**10**)	63
4	1-Phenyl-2-trimethylsilylacetylene (**19c**)	[(I*t*Bu)CuCl] (**18b**)	34

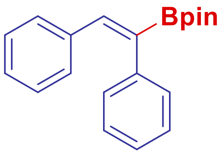

21a

(*Z*)-2-(1,2-Biphenylvinyl)-4,4,5,5-tetramethyl-1,3,2-dioxaborolane (21a). White solid. ^{1}H NMR (500 MHz, $CDCl_3$) δ 7.39 (s, 1H), 7.33 – 7.01 (m, 10H), 1.33 (s, 12H). ^{13}C NMR (126 MHz, $CDCl_3$) δ 143.49, 140.75, 137.31, 130.28, 129.18, 128.56, 128.17, 127.90, 126.59, 84.10, 25.13.

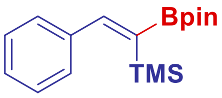

21b

(*Z*)-Trimethyl(2-phenyl-1-(4,4,5,5-tetramethyl-1,3,2-dioxaborolan-2-yl)vinyl)silane (21b). Colorless oil. ^{1}H NMR (500 MHz, $CDCl_3$) δ 7.39 – 7.36 (m, 2H), 7.34 – 7.31 (m, 1H), 7.29 – 7.23 (m, 2H), 6.94 (s, 1H), 1.38 (s, 12H), -0.02 (s, 9H). ^{13}C NMR (126 MHz, $CDCl_3$) δ 150.84, 144.02, 128.41, 127.89, 126.73, 84.14, 25.10, 0.31.

17. General Procedure for [Ag–NHC]-Catalyzed Hydroboration of Alkynes.

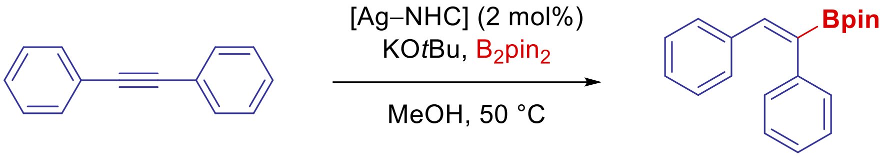

19a 21a

(*Z*)-2-(1,2-Biphenylvinyl)-4,4,5,5-tetramethyl-1,3,2-dioxaborolane (21a). An oven dried vial equipped with a stir bar was charged with diphenylacetelene (178.2 mg, 1.0 mmol, 1.0 equiv), bis(pinacolato)diboron (380.9 mg, 1.5 mmol, 1.5 equiv), KO*t*Bu (11.2 mg, 0.1 mmol, 0.1 equiv), [Ag–NHC] catalyst (2.0 mol%), placed under a positive pressure of argon, and subjected to three evacuation/backfilling cycles under high vacuum. MeOH (2.0 mL) was added with vigorous stirring and the reaction mixture was stirred at 50 °C for 15 h. After the indicate time the solvent was removed under reduce pressure. The sample was analyzed by ^{1}HNMR ($CDCl_3$, 500 MHz) and GC-MS to obtain conversion, selectivity and yield using internal standard and comparison with authentic samples. Purification by chromatography on silica gel (EtOAc/hexanes) afforded the title product. White solid. ^{1}H NMR (500 MHz, $CDCl_3$) δ 7.39 (s, 1H), 7.33 – 7.01 (m, 10H), 1.33 (s, 12H). ^{13}C NMR (126 MHz, $CDCl_3$) δ 143.49, 140.75, 137.31, 130.28, 129.18, 128.56, 128.17, 127.90, 126.59, 84.10, 25.13.

Table SI-3. *Comparative Reactivity of [(ItOct)AgCl] in Hydroboration of Alkynes.*

Entry	Alkyne	Catalyst	Yield (%)
1	Diphenylacetylene (**19a**)	[(I*t*Oct)AgCl] (**11**)	98
2	Diphenylacetylene (**19a**)	[(I*t*Bu)AgCl] (**18c**)	98

18. General Procedure for [Cu–NHC]-Catalyzed C–O Cross-Coupling.

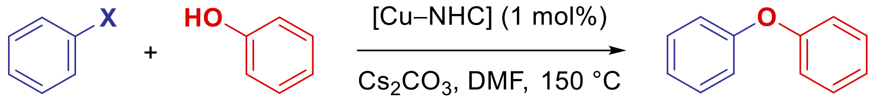

Diphenyl ether (24). An oven dried vial equipped with a stir bar was charged with phenol (47.1 mg, 0.50 mmol, 1.0 equiv), aryl halide (0.50 mmol, 1.0 equiv), Cs_2CO_3 (244.4 mg, 0.75 mmol, 1.5 equiv), [Cu–NHC] catalyst (1.0 mol%), DMF (1.0 mL), and stirred at 150 °C for 15 h. After the indicated time, the reaction mixture was cooled down to room temperature, diluted with CH_2Cl_2 (10 mL), filtered and concentrated. The sample was analyzed by ^{1}HNMR ($CDCl_3$, 500 MHz) and GC-MS to obtain conversion, selectivity and yield using internal standard and comparison with authentic samples. Purification by chromatography on silica gel (EtOAc/hexanes) afforded the title product. White solid. ^{1}H NMR (500 MHz, $CDCl_3$) δ 7.34 (dd, J = 8.3, 7.6 Hz, 4H), 7.10 (t, J = 7.4 Hz, 2H), 7.02 (d, J = 7.8 Hz, 4H). ^{13}C NMR (126 MHz, $CDCl_3$) δ 157.59, 130.07, 123.55, 119.23.

Table SI-4. *Comparative Reactivity of [(ItOct)CuCl] in C–O Cross-Coupling.*

Entry	Aryl Halide	Catalyst	Yield (%)
1	Iodobenzene (**22a**)	[(I*t*Oct)CuCl] (**10**)	83
2	Iodobenzene (**22a**)	[(I*t*Bu)CuCl] (**18b**)	73
3[a]	Iodobenzene (**22a**)	[(I*t*Oct)CuCl] (**10**)	54
4[a]	Iodobenzene (**22a**)	[(I*t*Bu)CuCl] (**18b**)	36
5	Bromobenzene (**22b**)	[(I*t*Oct)CuCl] (**10**)	29
6	Bromobenzene (**22b**)	[(I*t*Bu)CuCl] (**18b**)	21
7[b]	Bromobenzene (**22b**)	[(I*t*Oct)CuCl] (**10**)	81
8[b]	Bromobenzene (**22b**)	[CuCl]	33

[a]Under Argon. [b][Cu] (5 mol%)

19. General Procedure for [Pd–NHC]-Catalyzed Suzuki Cross-Coupling.

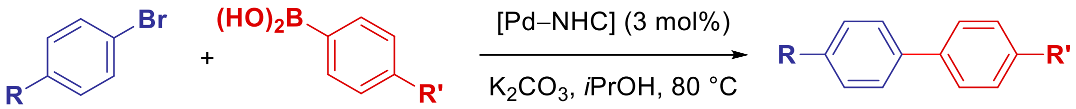

<u>*General procedure.*</u> An oven dried vial equipped with a stir bar was charged with an aryl bromide substrate (0.2 mmol, 1.0 equiv), boronic acid (0.4 mmol, 2.0 equiv), K_2CO_3 (82.8 mg, 0.6 mmol, 3.0 equiv), [Pd–NHC] catalyst (3.0 mol%), placed under a positive pressure of argon, and subjected to three evacuation/backfilling cycles under high vacuum. *i*PrOH (0.8 mL) was added and the reaction mixture was stirred at 80 °C for 15 h. After the indicated time, the reaction mixture was diluted with CH_2Cl_2 (10 mL), filtered, and concentrated. The sample was analyzed by ^{1}HNMR ($CDCl_3$, 500 MHz) and GC-MS to obtain conversion, selectivity and yield using internal standard and comparison with authentic samples. Purification by chromatography on silica gel (EtOAc/hexanes) afforded the title product.

Table SI-5. *Comparative Reactivity of [(ItOct)Pd(3-Cl-py)Cl_2] in Suzuki Cross-Coupling.*

Entry	Aryl Halide	Aryl Boronic Acid	Catalyst	Yield (%)
1	4-MeO-C_6H_4-Br (**25a**)	C_6H_5-B(OH)$_2$ (**26a**)	[(*I*tOct)Pd(3-Cl-py)Cl_2] (**14**)	75
2	4-MeO-C_6H_4-Br (**25a**)	C_6H_5-B(OH)$_2$ (**26a**)	[(*I*tBu)Pd(3-Cl-py)Cl_2] (**18d**)	55
3	4-MeOC-C_6H_4-Br (**25b**)	4-CF_3-C_6H_4-B(OH)$_2$ (**26b**)	[(*I*tOct)Pd(3-Cl-py)Cl_2] (**14**)	98
4	4-MeOC-C_6H_4-Br (**25b**)	4-CF_3-C_6H_4-B(OH)$_2$ (**26b**)	[(*I*tOct)Pd(3-Cl-py)Cl_2] (**18d**)	93

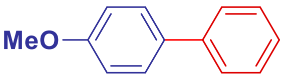

27a

4-Methoxy-1,1'-biphenyl (27a). White solid. ^{1}H NMR (500 MHz, $CDCl_3$) δ 7.59 – 7.53 (m, 4H), 7.43 (t, *J* = 7.7 Hz, 2H), 7.35 – 7.28 (m, 1H), 7.02 – 6.96 (m, 2H), 3.86 (s, 3H). ^{13}C NMR (126 MHz, $CDCl_3$) δ 159.48, 141.17, 134.12, 129.06, 128.49, 127.08, 126.99, 114.54, 55.68.

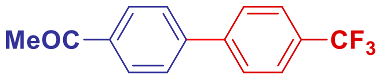

27b

4-Acetyl-4'-(trifluoromethyl)-1,1'-biphenyl (27b). White solid. ^{1}H NMR (500 MHz, $CDCl_3$) δ 8.06 (d, *J* = 8.4 Hz, 2H), 7.73 (s, 4H), 7.70 (d, *J* = 8.4 Hz, 2H), 2.65 (s, 3H). ^{13}C NMR (126 MHz, $CDCl_3$) δ 197.59, 144.20, 143.42, 136.61, 130.26 (q, J^F = 32.7 Hz), 129.06, 127.63, 127.48, 125.92 (q, J^F = 3.7 Hz), 124.13 (q, J^F = 272.1 Hz), 26.73. ^{19}F NMR (471 MHz, $CDCl_3$) δ -62.55.

20. General Procedure for [Pd–NHC]-Catalyzed Buchwald-Hartwig Cross-Coupling.

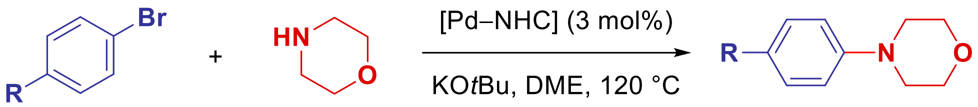

25 **28** **29**

<u>*General procedure.*</u> An oven dried vial equipped with a stir bar was charged with an aryl bromide substrate (0.2 mmol, 1.0 equiv), morpholine (34.8 mg, 0.4 mmol, 2.0 equiv), KO*t*Bu (56.1 mg, 0.5 mmol, 2.5 equiv), [Pd–NHC] catalyst (3 mol%), placed under a positive pressure of argon, and subjected to three evacuation/backfilling cycles under high vacuum. DME (1.0 mL) was added and the reaction mixture was stirred at 120 °C for 15 h. After the indicated time, the reaction mixture was diluted with CH_2Cl_2 (10 mL), washed with water (10 mL), extracted with CH_2Cl_2 (2 x 10 mL), dried and concentrated. The sample was analyzed by ^{1}HNMR ($CDCl_3$, 500 MHz) and GC-MS to obtain conversion, selectivity and yield using internal standard and comparison with authentic samples. Purification by chromatography on silica gel (EtOAc/hexanes) afforded the title product.

Table SI-6. *Comparative Reactivity of [(ItOct)Pd(3-Cl-py)Cl$_2$] in Buchwald-Hartwig Cross-Coupling.*

Entry	Aryl Halide	Catalyst	Yield (%)
1	4-MeO-C_6H_4-Br (**25a**)	[(I*t*Oct)Pd(3-Cl-py)Cl_2] (**14**)	86
2	4-MeO-C_6H_4-Br (**25a**)	[(I*t*Bu)Pd(3-Cl-py)Cl_2] (**18d**)	18
3	4-CF_3-C_6H_4-Br (**25c**)	[(I*t*Oct)Pd(3-Cl-py)Cl_2] (**14**)	98
4	4-CF_3-C_6H_4-Br (**25c**)	[(I*t*Bu)Pd(3-Cl-py)Cl_2] (**18d**)	52

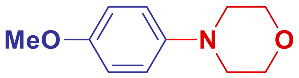

29a

4-(4-Methoxyphenyl)morpholine (29a). White solid. ^{1}H NMR (500 MHz, $CDCl_3$) δ 6.92 – 6.83 (m, 4H), 3.89 – 3.83 (m, 4H), 3.77 (s, 3H), 3.09 – 3.03 (m, 4H). ^{13}C NMR (126 MHz, $CDCl_3$) δ 154.34, 145.96, 118.17, 114.85, 67.37, 55.91, 51.18.

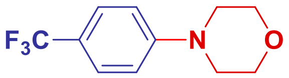

29b

4-(4-(Trifluoromethyl)phenyl)morpholine (29b). White solid. ^{1}H NMR (500 MHz, $CDCl_3$) δ 7.50 (d, *J* = 8.7 Hz, 2H), 6.92 (d, *J* = 8.7 Hz, 2H), 3.89 – 3.84 (m, 4H), 3.27 – 3.21 (m, 4H). ^{13}C NMR (126 MHz, $CDCl_3$) δ 153.67, 126.78 (q, J^F = 3.7 Hz), 125.01 (q, J^F = 270.7 Hz), 121.33 (q, J^F = 32.8 Hz), 114.65, 66.97, 48.50. ^{19}F NMR (471 MHz, $CDCl_3$) δ -61.40.

Crystallographic Studies

Chart SI-1. *Crystal Data and Structure Refinement Summary for* ***9****.*

Compound	**9**
Crystal Data	
Chemical formula	$C_{19}H_{36}AuClN_2$
M_r	524.91
Crystal system, space group	Monoclinic, $P2_1/n$
Temperature (K)	100
a, *b*, *c* (Å)	12.5347 (16), 13.5209 (17), 13.0674 (17)
β (°)	93.446 (3)
V (Å^3)	2210.7 (5)
Z	4
Radiation type	Cu *K*α
μ (mm^{-1})	13.61
Crystal size (mm)	0.32 × 0.26 × 0.23
Data collection	
Diffractometer	Bruker *SMART* CCD Apex-II area-detector
Absorption correction	Numerical<br>*SADABS* 2016/2: Krause, L., Herbst-Irmer, R., Sheldrick G.M. & Stalke D., J. Appl. Cryst. 48 (2015) 3-10
T_{min}, T_{max}	0.064, 0.267
No. of measured, independent and observed [$I > 2\sigma(I)$] reflections	24461, 3890, 3864
R_{int}	0.034
$(\sin \theta/\lambda)_{max}$ (Å^{-1})	0.595
Refinement	
$R[F^2 > 2\sigma(F^2)]$, $wR(F^2)$, S	0.021, 0.049, 1.21
No. of reflections	3890
No. of parameters	219
H-atom treatment	H-atom parameters constrained
$\Delta\rho_{max}$, $\Delta\rho_{min}$ (e Å^{-3})	0.59, −0.53

Computer programs: *APEX* 2 (Bruker, 2006), *APEX* 2, *SAINT* (Bruker, 2005), *SHELXL2016*/6 (Sheldrick, 2016), *SHELXTL*.

Chart SI-2. *Crystal Data and Structure Refinement Summary for* ***10****.*

Compound	**10**
Crystal Data	
Chemical formula	$C_{19}H_{36}ClCuN_2$
M_r	391.49
Crystal system, space group	Monoclinic, $P2_1/n$
Temperature (K)	293
a, *b*, *c* (Å)	12.6916 (6), 13.5030 (7), 13.0909 (6)
β (°)	95.351 (4)
V (Å^3)	2233.67 (19)
Z	4
Radiation type	Mo *K*α
μ (mm^{-1})	1.10
Crystal size (mm)	0.4 × 0.25 × 0.1
Data collection	
Diffractometer	Xcalibur
Absorption correction	Multi-scan
T_{min}, T_{max}	0.974, 1.000
No. of measured, independent and observed [$I > 2\sigma(I)$] reflections	14984, 4387, 1951
R_{int}	0.069
$(\sin \theta/\lambda)_{max}$ (Å^{-1})	0.617
Refinement	
$R[F^2 > 2\sigma(F^2)]$, $wR(F^2)$, S	0.046, 0.106, 0.82
No. of reflections	4387
No. of parameters	218
H-atom treatment	H-atom parameters constrained
$\Delta\rho_{max}$, $\Delta\rho_{min}$ (e Å^{-3})	0.35, −0.22

Computer programs: *CrysAlis CCD* (Oxford Diffraction Ltd., 2008), *SHELXS2014*/7 (Sheldrick, 2014), *SHELXL2014*/7 (Sheldrick, 2014), *SHELXTL* (Sheldrick, 2008).

Chart SI-3. *Crystal Data and Structure Refinement Summary for **11**.*

Compound	**11**
Crystal Data	
Chemical formula	$C_{19}H_{36}AgClN_2$
M_r	435.82
Crystal system, space group	Monoclinic, $P2_1/n$
Temperature (K)	293
a, *b*, *c* (Å)	12.9804 (3), 13.4131 (3), 13.1899 (3)
β (°)	90.467 (2)
V (Å^3)	2296.38 (9)
Z	4
Radiation type	Mo $K\alpha$
μ (mm^{-1})	1.00
Crystal size (mm)	0.4 × 0.3 × 0.2
Data collection	
Diffractometer	Xcalibur
Absorption correction	Multi-scan
T_{min}, T_{max}	0.979, 1.000
No. of measured, independent and observed [$I > 2\sigma(I)$] reflections	15291, 4501, 3372
R_{int}	0.022
$(\sin \theta/\lambda)_{max}$ (Å^{-1})	0.617
Refinement	
$R[F^2 > 2\sigma(F^2)]$, $wR(F^2)$, S	0.028, 0.071, 0.99
No. of reflections	4501
No. of parameters	218
H-atom treatment	H-atom parameters constrained
$\Delta\rho_{max}$, $\Delta\rho_{min}$ (e Å^{-3})	0.49, −0.47

Computer programs: *CrysAlis CCD* (Oxford Diffraction Ltd., 2008), *SHELXS2014*/7 (Sheldrick, 2014), *SHELXL2014*/7 (Sheldrick, 2014), *SHELXTL* (Sheldrick, 2008).

Chart SI-4. *Crystal Data and Structure Refinement Summary for* ***12****.*

Compound	**12**
Crystal Data	
Chemical formula	$C_{19}H_{36}N_2Se$
M_r	371.46
Crystal system, space group	Orthorhombic, $Pca2_1$
Temperature (K)	293
a, *b*, *c* (Å)	32.8739 (10), 10.3807 (3), 12.1090 (3)
V (Å^3)	4132.2 (2)
Z	8
Radiation type	Mo $K\alpha$
μ (mm^{-1})	1.82
Crystal size (mm)	0.3 × 0.2 × 0.1
Data collection	
Diffractometer	Xcalibur
Absorption correction	Multi-scan
T_{min}, T_{max}	0.924, 1.000
No. of measured, independent and observed [$I > 2\sigma(I)$] reflections	26934, 5729, 3327
R_{int}	0.047
$(\sin \theta/\lambda)_{max}$ (Å^{-1})	0.617
Refinement	
$R[F^2 > 2\sigma(F^2)]$, $wR(F^2)$, S	0.039, 0.084, 0.84
No. of reflections	5729
No. of parameters	417
No. of restraints	1
H-atom treatment	H-atom parameters constrained
$\Delta\rho_{max}$, $\Delta\rho_{min}$ (e Å^{-3})	1.04, −0.53
Absolute structure	Flack x determined using 559 quotients [(I+)-(I-)]/[(I+)+(I-)] (Parsons, Flack and Wagner, Acta Cryst. B69 (2013) 249-259).
Absolute structure parameter	0.039 (10)

Computer programs: *CrysAlis CCD* (Oxford Diffraction Ltd., 2008), *SHELXS2014*/7 (Sheldrick, 2014), *SHELXL2014*/7 (Sheldrick, 2014), *SHELXTL* (Sheldrick, 2008).

Chart SI-5. *Crystal Data and Structure Refinement Summary for **14**.*

Compound	**14**
Crystal Data	
Chemical formula	$C_{24}H_{40}Cl_3N_3Pd$
M_r	583.34
Crystal system, space group	Orthorhombic, $P2_12_12_1$
Temperature (K)	293
a, *b*, *c* (Å)	12.6940 (3), 14.2272 (3), 15.3472 (4)
V (Å^3)	2771.71 (11)
Z	4
Radiation type	Mo *K*α
μ (mm^{-1})	0.98
Crystal size (mm)	0.3 × 0.2 × 0.1
Data collection	
Diffractometer	Xcalibur
Absorption correction	Multi-scan
T_{min}, T_{max}	0.932, 1.000
No. of measured, independent and observed [$I > 2\sigma(I)$] reflections	19014, 5434, 4778
R_{int}	0.021
$(\sin \theta/\lambda)_{max}$ (Å^{-1})	0.617
Refinement	
$R[F^2 > 2\sigma(F^2)]$, $wR(F^2)$, S	0.019, 0.039, 0.94
No. of reflections	5434
No. of parameters	290
H-atom treatment	H-atom parameters constrained
$\Delta\rho_{max}$, $\Delta\rho_{min}$ (e Å^{-3})	0.23, −0.29
Absolute structure	Flack x determined using 1977 quotients [(I+)-(I-)]/[(I+)+(I-)] (Parsons, Flack and Wagner, Acta Cryst. B69 (2013) 249-259).
Absolute structure parameter	−0.015 (8)

Computer programs: *CrysAlis CCD* (Oxford Diffraction Ltd., 2008), *SHELXS2014*/7 (Sheldrick, 2014), *SHELXL2014*/7 (Sheldrick, 2014), *SHELXTL* (Sheldrick, 2008).

ORTEP Structures of 9-12 and 14

Chart SI-6 – Front View. *ORTEP Structure of* ***9*** *(CCDC 2239373).*

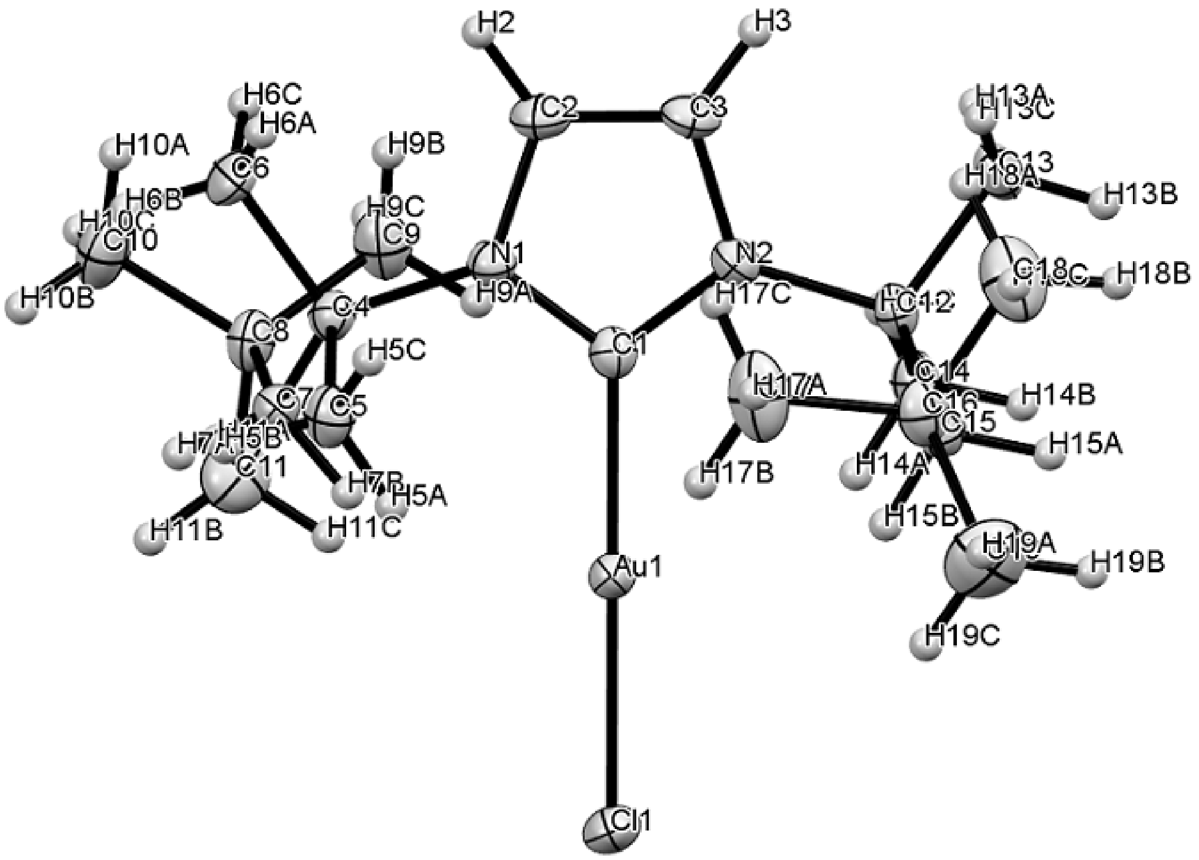

50% ellipsoids. Selected bond lengths (Å), **9**, see Table SI-7.

Chart SI-7 – Side View. *ORTEP Structure of **9** (CCDC 2239373).*

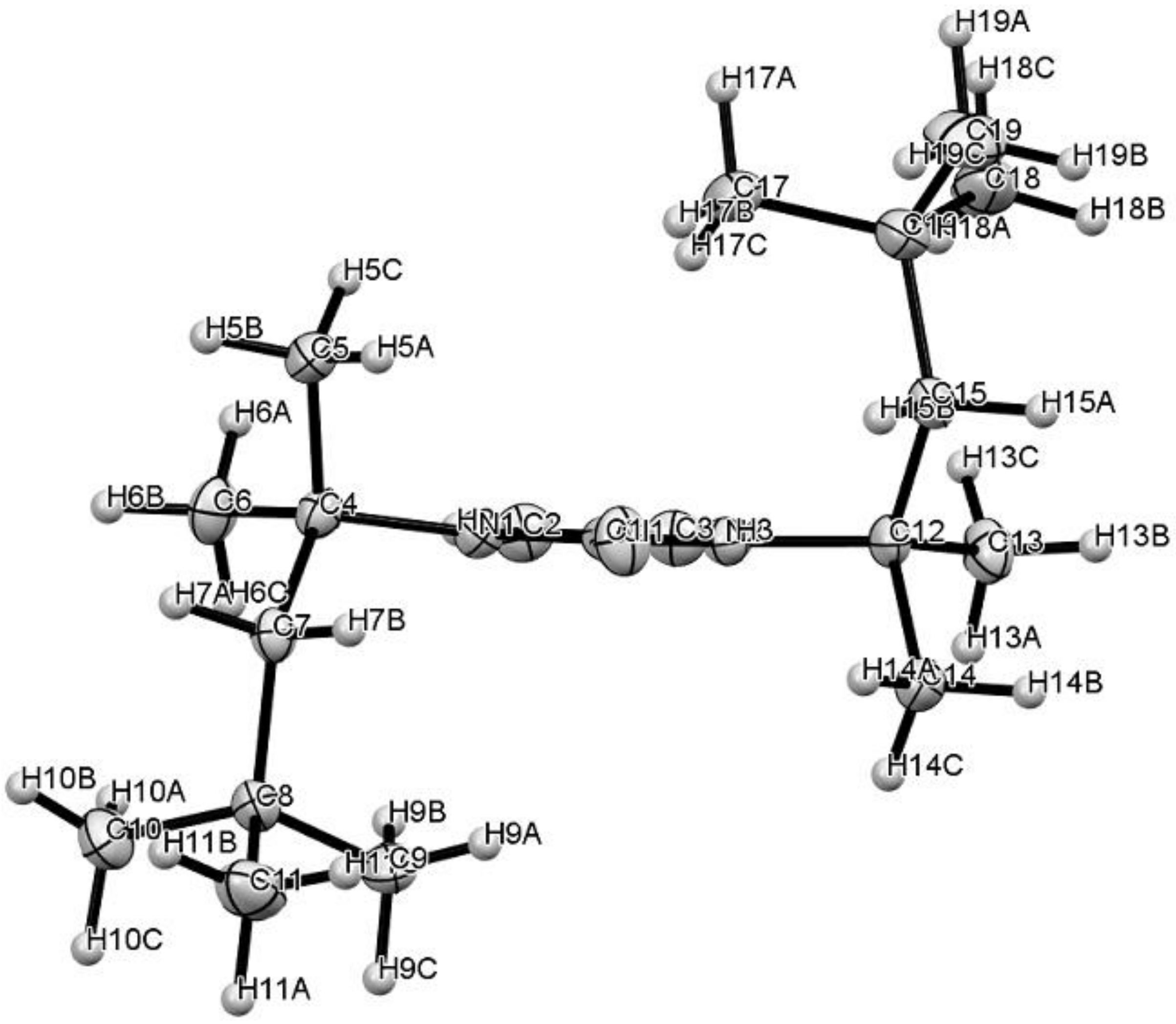

50% ellipsoids. Selected bond lengths (Å), **9**, see Table SI-7.

Chart SI-8 – Front View. *ORTEP Structure of **10** (CCDC 2239374).*

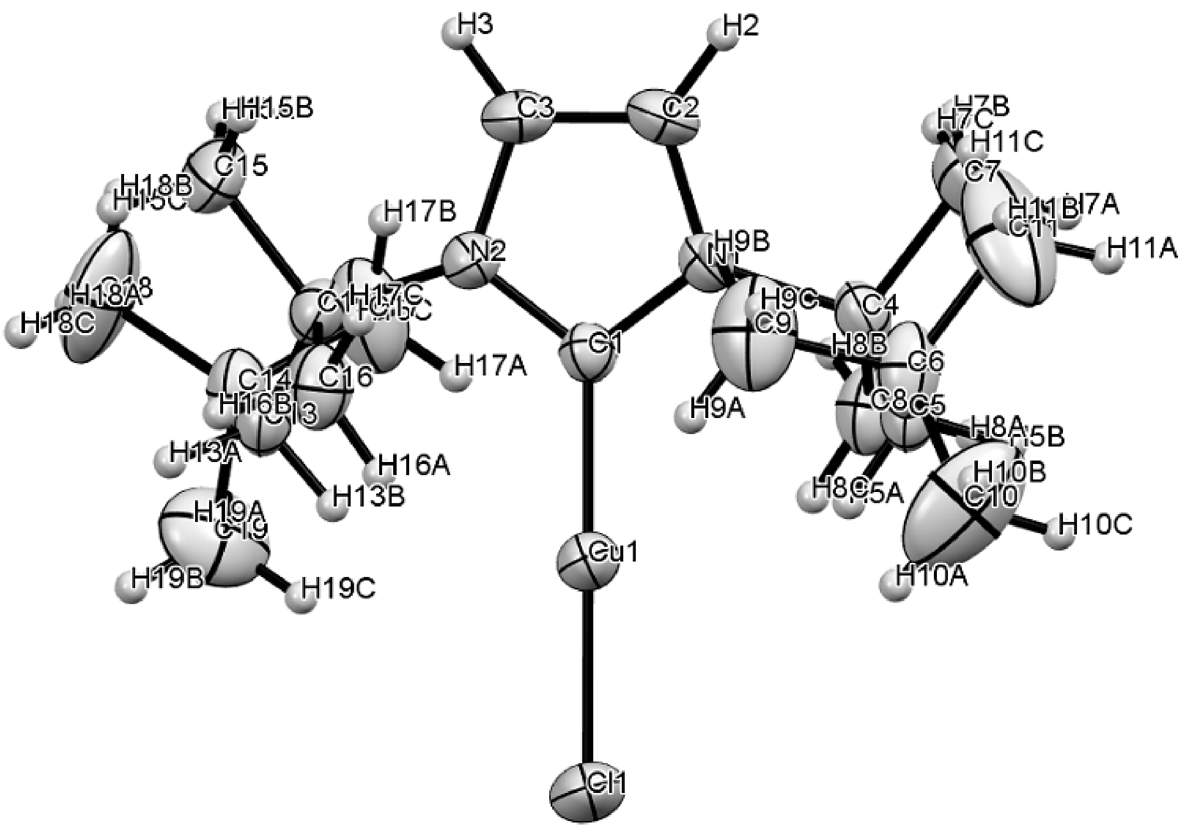

30% ellipsoids. Selected bond lengths (Å), **10**, see Table SI-7.

Chart SI-9 – Side View. *ORTEP Structure of* ***10*** *(CCDC 2239374).*

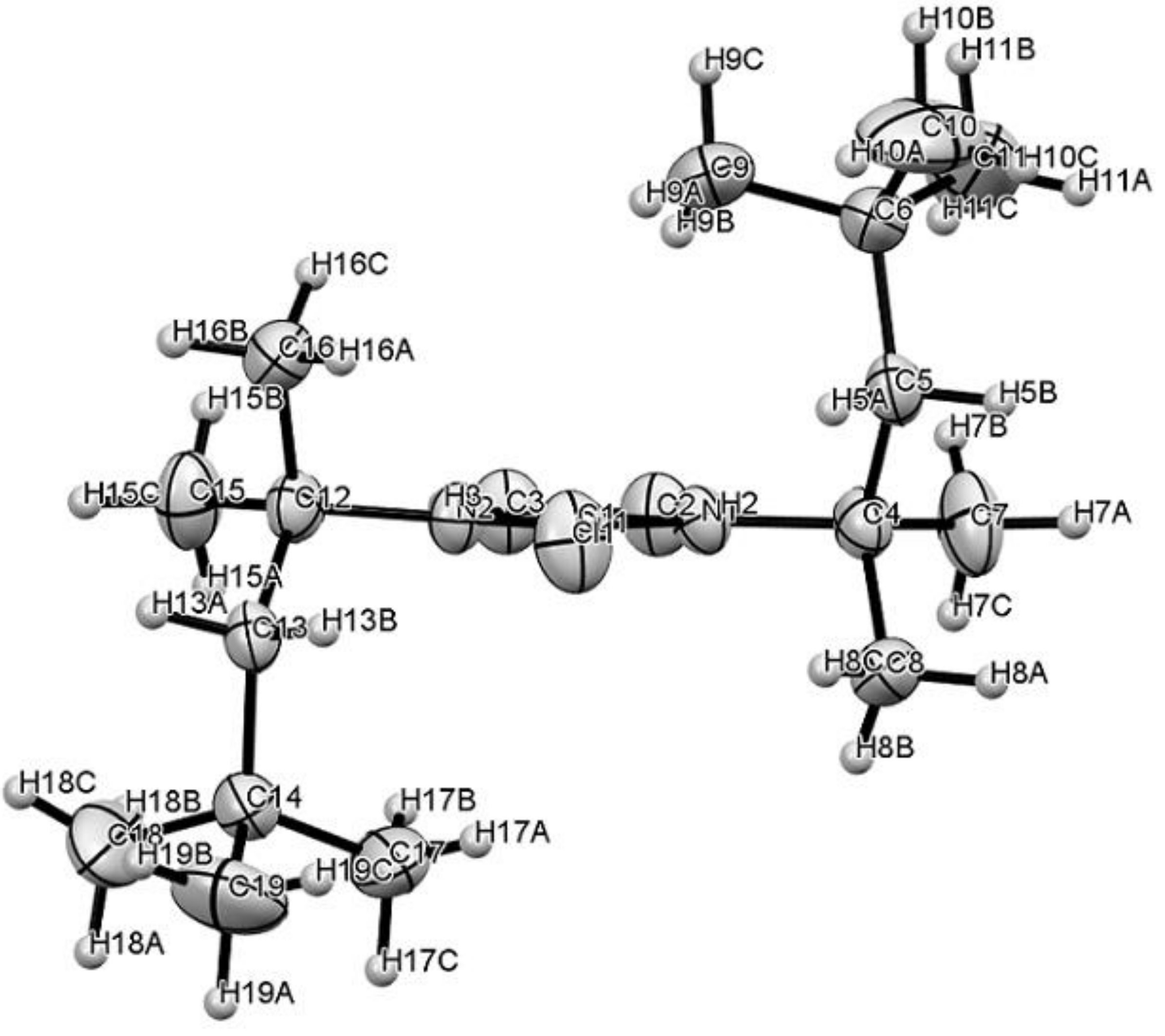

30% ellipsoids. Selected bond lengths (Å), **10**, see Table SI-7.

Chart SI-10 – Front View. *ORTEP Structure of* ***11*** *(CCDC 2239375).*

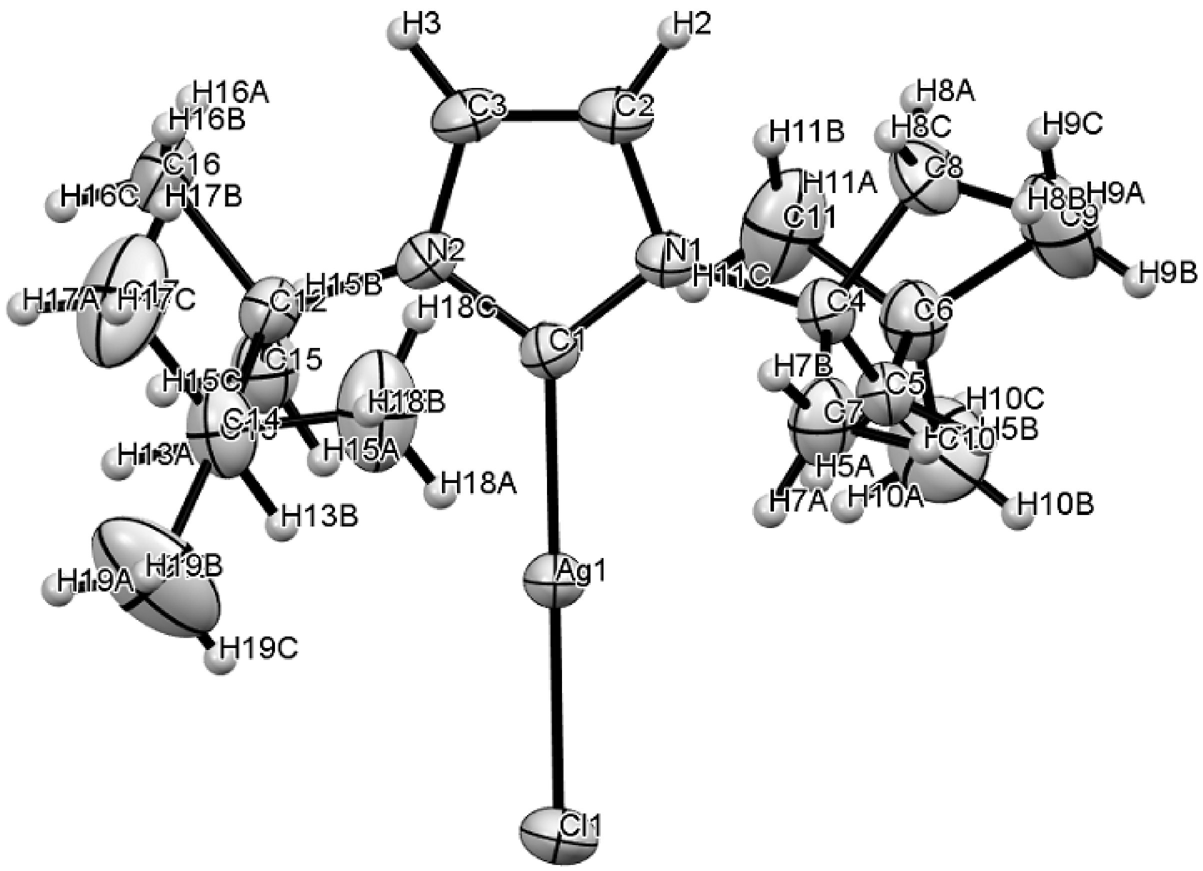

30% ellipsoids. Selected bond lengths (Å), **11**, see Table SI-7.

Chart SI-11 – Side View. *ORTEP Structure of* ***11*** *(CCDC 2239375).*

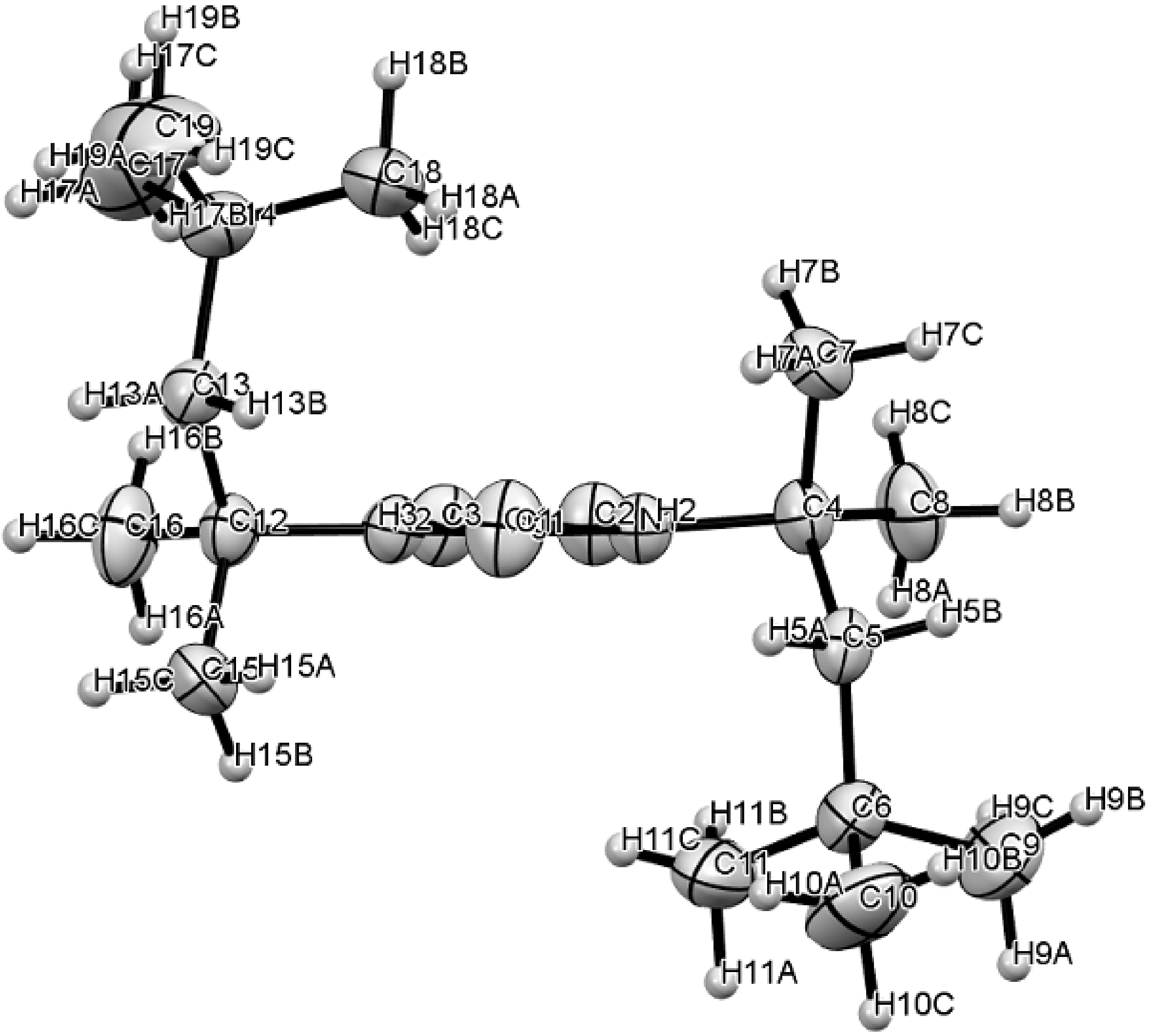

30% ellipsoids. Selected bond lengths (Å), **11**, see Table SI-7.

Chart SI-12 – Front View. *ORTEP Structure of* ***12*** *(CCDC 2239376).*

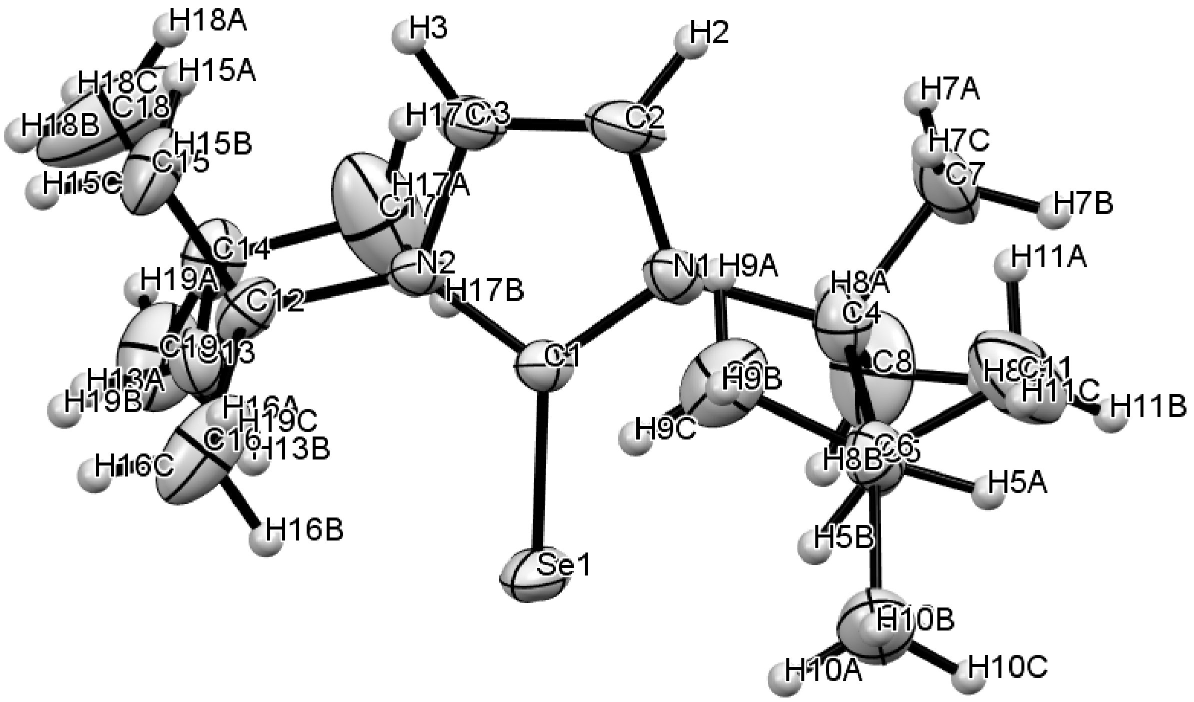

30% ellipsoids. Selected bond lengths (Å), **12**, see Table SI-7. Two independent molecules were observed in the unit cell. One independent molecule shown for clarity.

Chart SI-13 – Side View. *ORTEP Structure of **12** (CCDC 2239376).*

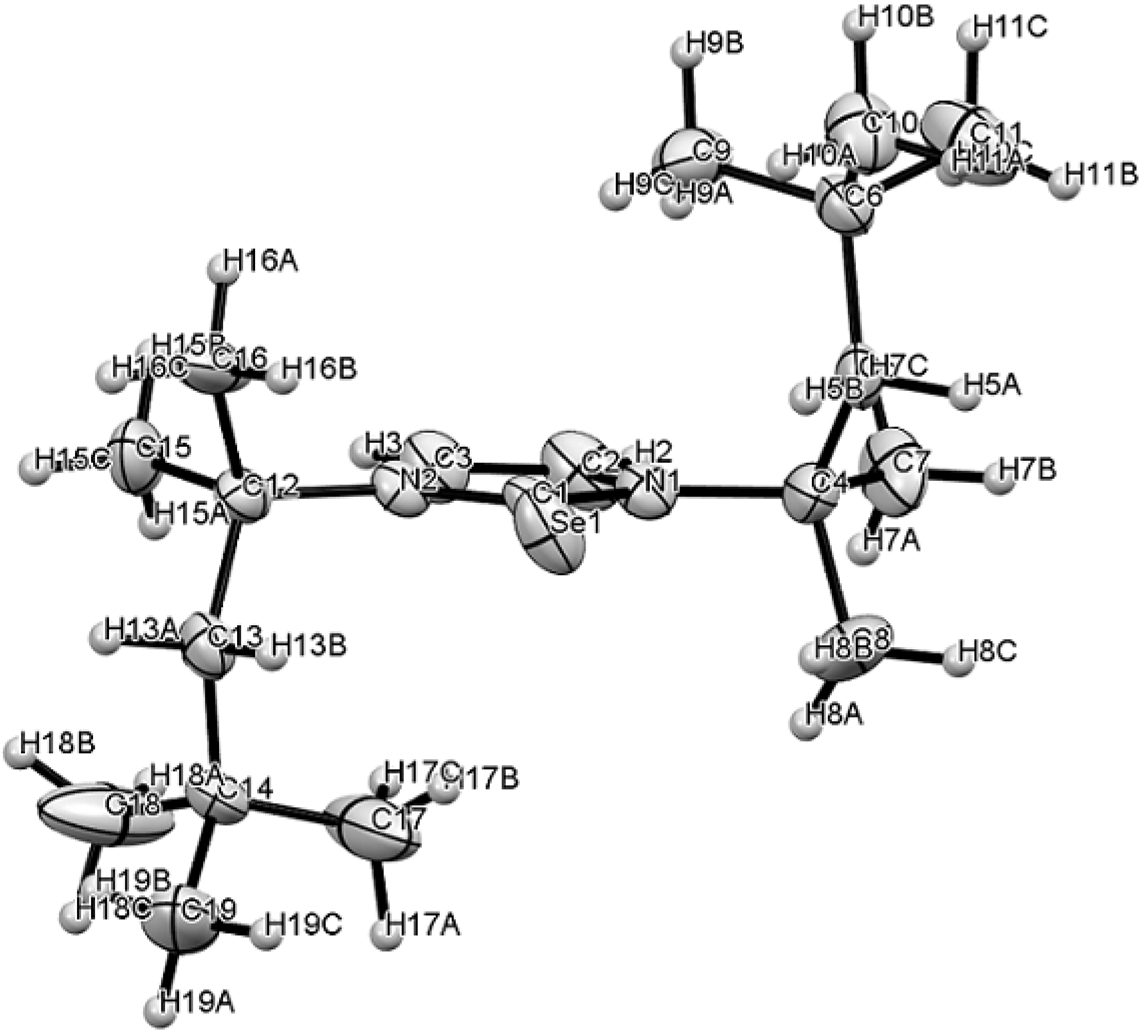

30% ellipsoids. Selected bond lengths (Å), **12**, see Table SI-7. Two independent molecules were observed in the unit cell. One independent molecule shown for clarity.

Chart SI-14 – Front View. *ORTEP Structure of **14** (CCDC 2239377).*

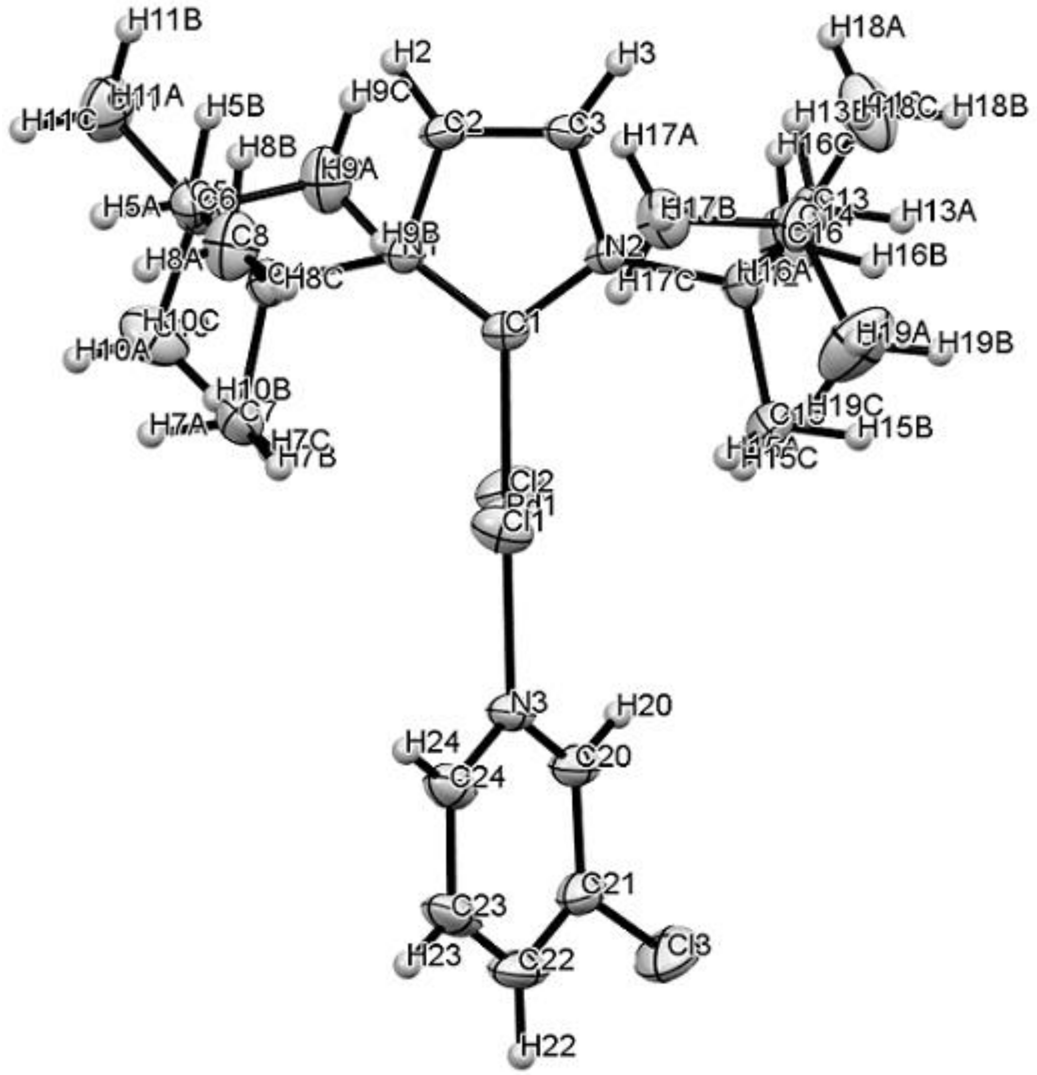

30% ellipsoids. Selected bond lengths (Å), **14**, see Table SI-7.

Chart SI-15 – Side View. *ORTEP Structure of* ***14*** *(CCDC 2239377).*

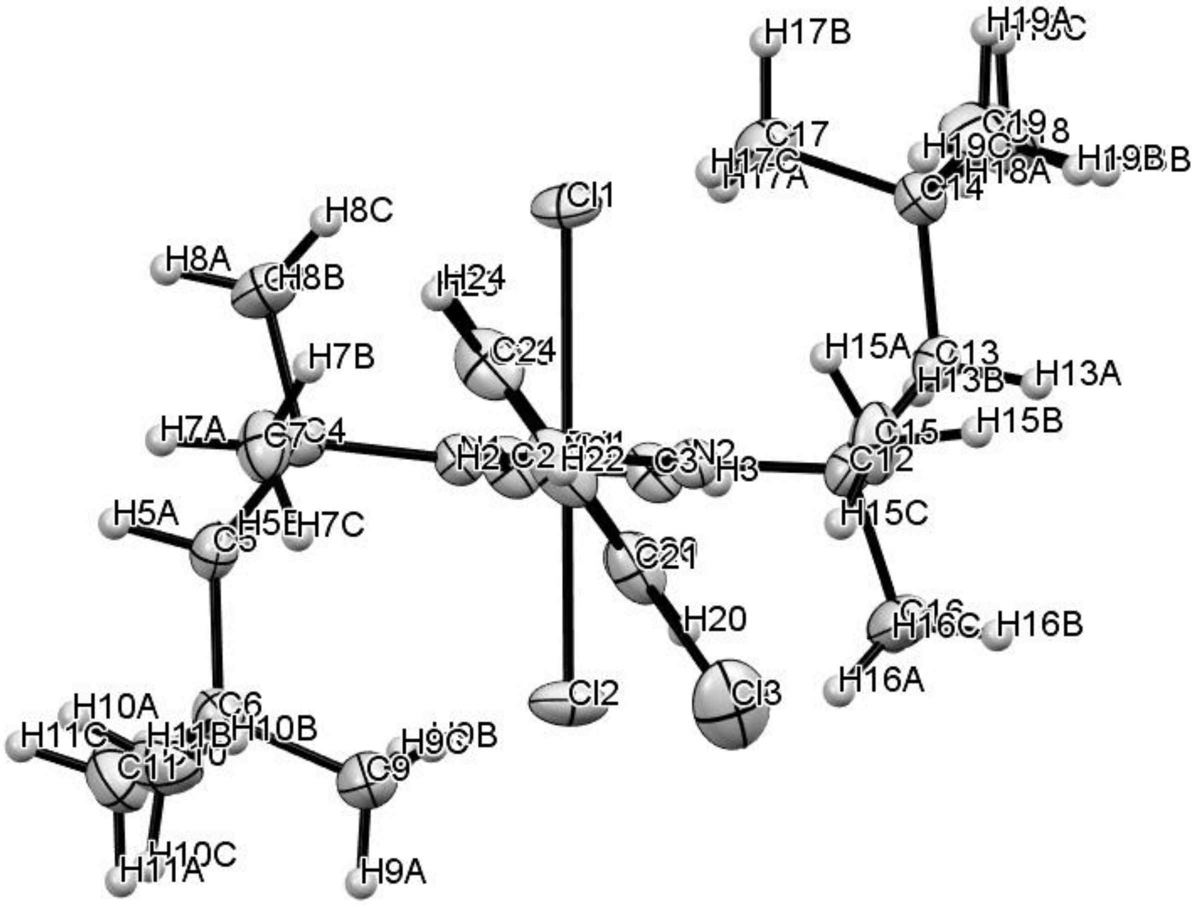

30% ellipsoids. Selected bond lengths (Å), **14**, see Table SI-7.

Table SI-7. Selected Structural Parameters of 9-12 and 14 Determined by X-ray Crystallography

entry	compound	M–C [Å]	M–X [Å]	$C_{(c)}$–N [Å]	C–N [Å]	$C_{(c)}$–N–C [°]	C–N–C [°]	C–N–$C_{(c)}$–M [°]
1	[Au(I*t*Oct)Cl]	2.007	2.282	1.358	1.500	125.95	124.65	3.59
2	[Cu(I*t*Oct)Cl]	2.080	2.304	1.355	1.501	124.80	125.05	2.59
3	[Ag(I*t*Oct)Cl]	1.886	2.084	1.353	1.488	124.37	125.84	2.55
4[a]	[Se(I*t*Oct)]	1.845	-	1.366	1.495	129.01	122.62	1.76
5[a]	[Se(I*t*Oct)]	1.869	-	1.351	1.511	128.65	122.96	2.66
6	[Se(I*t*Oct)] avg.	1.857	-	1.358	1.503	128.83	122.79	2.21
7[b]	[Pd(I*t*Oct)(3-py)Cl_2]	1.969	2.129	1.358	1.499	131.24	119.74	5.70

[a]Two independent molecules were observed in the unit cell. [b]Pd-3-Cl-py bond length. Pd–Cl1, 2.317 Å; Pd–Cl2, 2.309 Å. $C_{(c)}$ indicates the carbene carbon atom. The values listed for bond lengts and angles are an average for unsymmetrical bond lengths and angles.

Additional Discussion. Interestingly, we found that there are good linear correlations between the structural parameters in the studied series of [M–I*t*Oct] complexes. As shown in Chart SI-16, there is an excellent inverse linear correlation between the $C_{(c)}$–N–C and the C–N–C angle in the series of [Au(I*t*Oct)Cl], [Cu(I*t*Oct)Cl], [Ag(I*t*Oct)Cl], [Se(I*t*Oct)] (two independent molecules), [Pd(I*t*Oct)(3-py)Cl_2] as determined by the x-ray crystallography (Chart SI-16, Y = -0.794X + 224.6, R^2 = 0.954).

Furthermore, there is an excellent linear correlation between the C–N–$C_{(c)}$–M dihedral angle and the $C_{(c)}$–N–C angle in the series of [Au(I*t*Oct)Cl], [Cu(I*t*Oct)Cl], [Ag(I*t*Oct)Cl], [Pd(I*t*Oct)(3-py)Cl_2] (Chart SI-17, Y = 0.461X – 54.72, R^2 = 0.982).

Moreover, there is a good linear correlation between the M–$X_{(trans)}$ bond length and the M–$C_{(c)}$ bond length (not shown, Y = 1.242X – 0.267, R^2 = 0.838) and between the N–$C_{(alkyl)}$ bond length and the M–$C_{(c)}$ bond length (not shown, Y = 0.067X + 1.365, R^2 = 0.8239) in the series of [Au(I*t*Oct)Cl], [Cu(I*t*Oct)Cl], [Ag(I*t*Oct)Cl], [Pd(I*t*Oct)(3-py)Cl_2].

Overall, these features indicate that complexation of the I*t*Oct ligand with different metals (Au(I), Cu(I), Ag(I), Pd(II)) is reflected by the structural changes of the NHC ligand that increase the steric environment around the metal center.

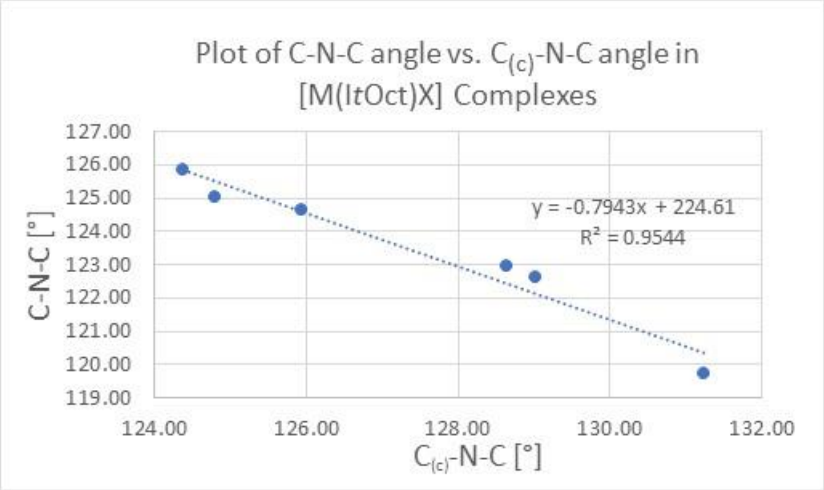

Chart SI-16. *Plot of $C_{(c)}$-N-C angle vs. C-N-C angle in [M(ItOct)X] complexes*

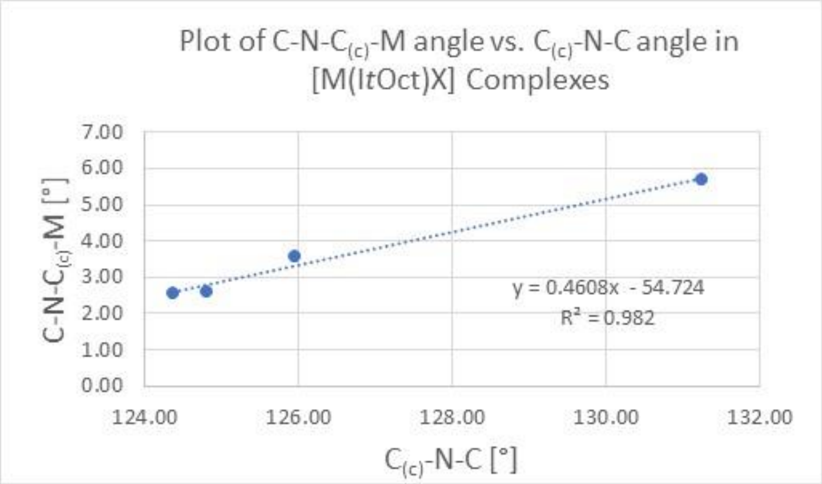

Chart SI-17. *Plot of C-N-C$_{(c)}$-M angle vs. C$_{(c)}$-N-C angle in [M(ItOct)X] complexes*

Furthermore, there is a linear relationship between the structural and energetic parameters in the series of [M–I*t*Oct] complexes as evaluated by using NHC–Cu(I)–Cl complexes (B3LYP 6-311++g(d,p) level, *vide infra*). As shown, there is an inverse linear correlation between the Cu–C$_{(c)}$ bond length and the HOMO energy levels in the series of [Cu(I*t*Oct)Cl], [Cu(SI*t*Oct)Cl], [Cu(I*t*Bu)Cl], [Cu(SI*t*Bu)] (Chart SI-18, Y = -0.075X + 0.941, R^2 = 0.964), which is consistent with the stronger σ-donation leading to shortening of the M–C$_{(c)}$ bond. Further studies on the properties of NHC ligands are underway in our laboratory and these findings will be reported in due course.

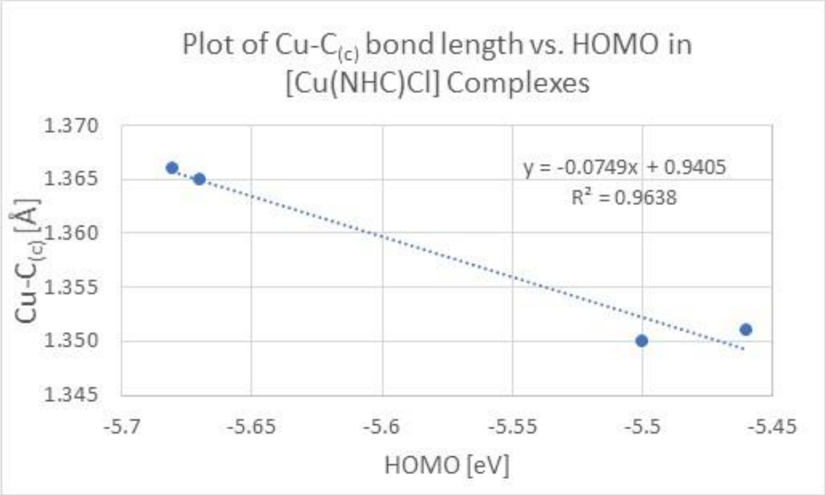

Chart SI-18. *Plot of Cu-C$_{(c)}$ bond length vs. HOMO in [Cu(NHC)Cl] complexes*

Computational Methods

Computational Methods. All of the calculations were performed using Gaussian 09 suite of programs. All of the geometry optimizations were performed at the B3LYP level of theory in the gas phase with the 6-311++G(d,p) basis set. This level has been shown to be accurate in predicting structures and electronic properties of NHCs (*Angew. Chem. Int. Ed.* **2018**, *57*, 8603). For geometry optimizations, we employed the X-ray structures of the corresponding metal complexes as the starting geometry and performed full optimization. The absence of imaginary frequencies was used to characterize the structures as minima on the potential energy surface. All of the optimized geometries were verified as minima (no imaginary frequencies). Energetic parameters were calculated under standard conditions (298.15 K and 1 atm). Structural representations were generated using CYLview software (Legault, C. Y. CYLview version 1.0 BETA, University of Sherbrooke). All other representations were generated using GaussView (GaussView, version 5, Dennington, R.; Keith, T.; Millam, J. Semichem Inc., Shawnee Mission, KS, 2009) or ChemCraft software (Andrienko, G. L. ChemCraft version b562a, https://www.chemcraftprog.com).

Full Reference for Gaussian 09

Gaussian 09, Revision D.01, Frisch, M. J.; Trucks, G. W.; Schlegel, H. B.; Scuseria, G. E.; Robb, M. A.; Cheeseman, J. R.; Scalmani, G.; Barone, V.; Mennucci, B.; Petersson, G. A.; Nakatsuji, H.; Caricato, M.; Li, X.; Hratchian, H. P.; Izmaylov, A. F.; Bloino, J.; Zheng, G.; Sonnenberg, J. L.; Hada, M.; Ehara, M.; Toyota, K.; Fukuda, R.; Hasegawa, J.; Ishida, M.; Nakajima, T.; Honda, Y.; Kitao, O.; Nakai, H.; Vreven, T.; Montgomery, J. A., Jr.; Peralta, J. E.; Ogliaro, F.; Bearpark, M.; Heyd, J. J.; Brothers, E.; Kudin, K. N.; Staroverov, V. N.; Kobayashi, R.; Normand, J.; Raghavachari, K.; Rendell, A.; Burant, J. C.; Iyengar, S. S.; Tomasi, J.; Cossi, M.; Rega, N.; Millam, M. J.; Klene, M.; Knox, J. E.; Cross, J. B.; Bakken, V.; Adamo, C.; Jaramillo, J.; Gomperts, R.; Stratmann, R. E.; Yazyev, O.; Austin, A. J.; Cammi, R.; Pomelli, C.; Ochterski, J. W.; Martin, R. L.; Morokuma, K.; Zakrzewski, V. G.; Voth, G. A.; Salvador, P.; Dannenberg, J. J.; Dapprich, S.; Daniels, A. D.; Farkas, Ö.; Foresman, J. B.; Ortiz, J. V.; Cioslowski, J.; Fox, D. J. Gaussian, Inc., Wallingford CT, 2009.

Chart SI-19. Optimized Geometries of [Cu(NHC)Cl] Complexes Referred to from the Main Manuscript at the B3LYP 6-311++g(d,p) Level (Front and Side View)[a]

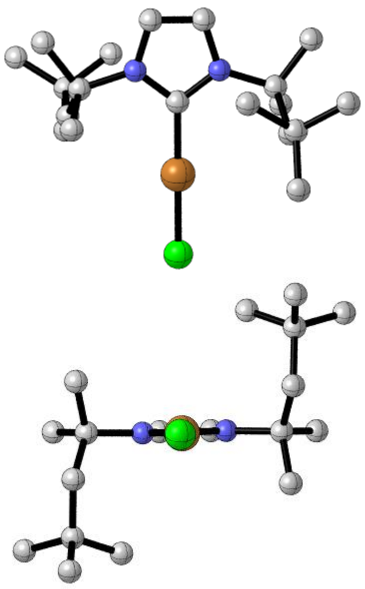

(I*t*Oct)CuCl

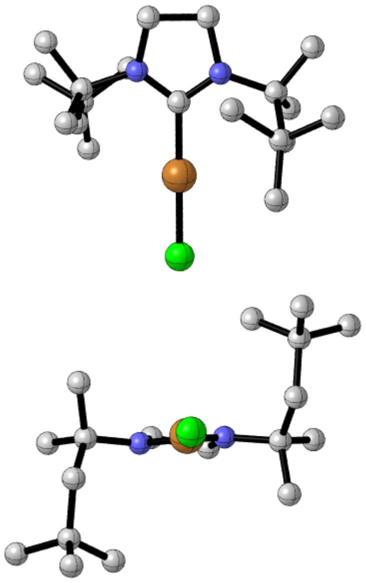

(SI*t*Oct)CuCl

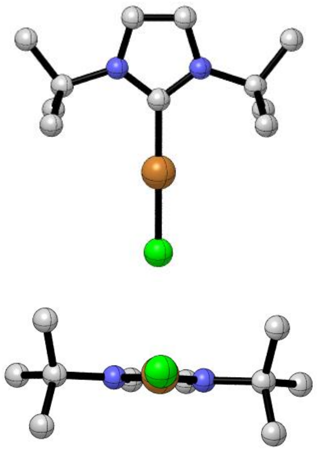

(I*t*Bu)CuCl

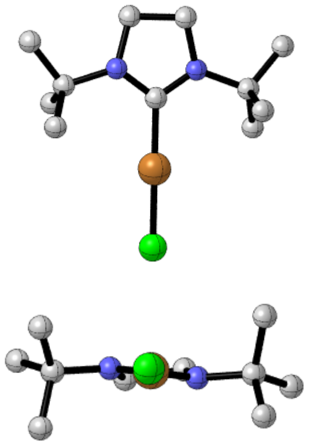

(SI*t*Bu)CuCl

[a]Hydrogens atoms omitted for clarity. Note the striking difference is the steric environment between the I*t*Oct and I*t*Bu ligands (also SI*t*Oct vs. SI*t*Bu), and non-planarity of the imidazolinylidene ring (SI*t*Oct, SI*t*Bu vs. I*t*Oct, I*t*Bu).

Table SI-8. Selected Structural Parameters of [Cu(NHC)Cl] Complexes and Carbenes Referred to from the Main Manuscript at the B3LYP 6-311++g(d,p) Level

entry	compound	Cu–C [Å]	Cu–Cl [Å]	$C_{(c)}$–N [Å]	C–N [Å]	$C_{(c)}$–N–C [°]	C–N–C [°]	C–N–$C_{(c)}$–Cu [°]
1	(I*t*Oct)CuCl	1.921	2.135	1.366	1.511	124.81	124.81	2.40
2	(SI*t*Oct)CuCl	1.927	2.137	1.350	1.506	125.05	121.54	10.22
3	(I*t*Bu)CuCl	1.919	2.133	1.365	1.507	124.43	125.11	0.00
4	(SI*t*Bu)CuCl	1.924	2.136	1.351	1.501	124.76	121.37	11.28
5	I*t*Oct	-	-	1.365	1.498	121.99	126.04	-
6	SI*t*Oct	-	-	1.348	1.493	122.34	121.51	-
7	I*t*Bu	-	-	1.365	1.495	121.63	126.39	-
8	SI*t*Bu	-	-	1.348	1.486	122.77	121.20	-

Table SI-9. HOMO and LUMO Energy Levels of I*t*Oct NHCs Calculated at the B3LYP 6-311++g(d,p) Level

entry	compound	orbital	E [au]	E [eV]	E [kcal/mol]	ΔE [eV]
1	I*t*Oct	HOMO	-0.2088	-5.68	-131.02	
2[a]	I*t*Oct	LUMO	0.0022	0.06	1.38	-5.74
3	SI*t*Oct	HOMO	-0.2023	-5.50	-126.94	
4[a]	SI*t*Oct	LUMO	-0.0014	-0.04	-0.88	-5.47
5	I*t*Bu	HOMO	-0.2082	-5.67	-130.65	
6[b]	I*t*Bu	LUMO	0.0134	0.36	8.41	-6.03
7	SI*t*Bu	HOMO	-0.2008	-5.46	-126.00	
8[a]	SI*t*Bu	LUMO	0.0053	0.14	3.33	-5.61

[a]LUMO+2 due to required π-symmetry. [b]LUMO+3 due to required π-symmetry.

Chart SI-20. Graphical Representation of Frontier Orbitals of Representative I*t*Oct NHCs and Orbital Energies (eV) Referred to from the Main Manuscript at the B3LYP 6-311++g(d,p) Level

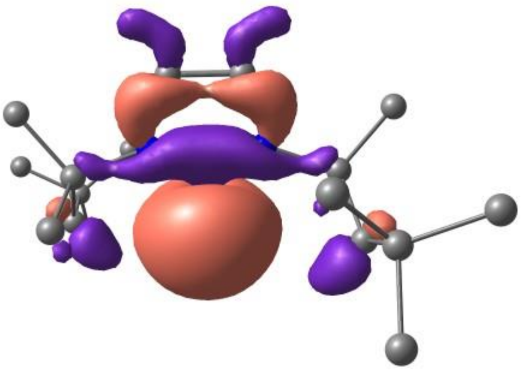

HOMO$_{\mathrm{I}t\mathrm{Oct}}$

-5.68

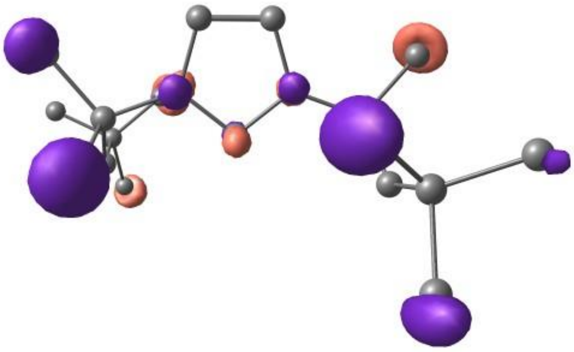

LUMO+2$_{\mathrm{I}t\mathrm{Oct}}$

0.06

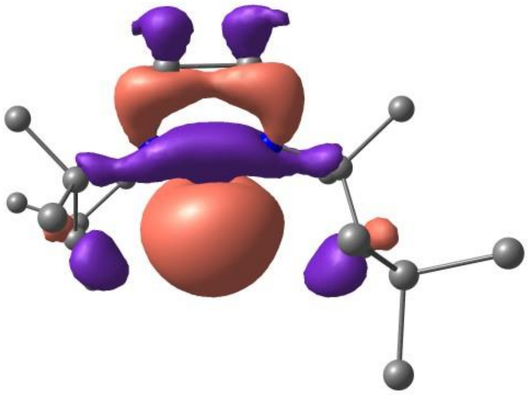

HOMO$_{\mathrm{SItOct}}$

-5.50

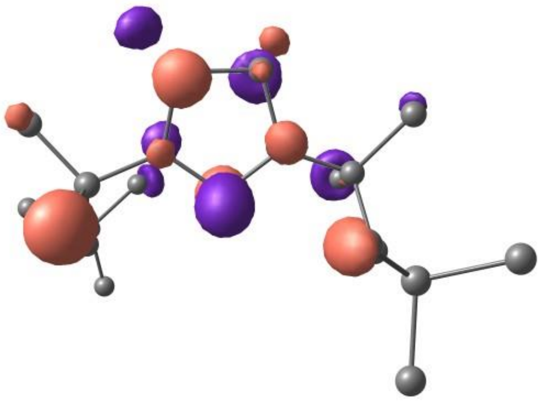

LUMO+2$_{\mathrm{SI}t\mathrm{Oct}}$

-0.04

Chart SI-20 cont. Graphical Representation of Frontier Orbitals of Representative I*t*Bu NHCs and Orbital Energies (eV) Referred to from the Main Manuscript at the B3LYP 6-311++g(d,p) Level

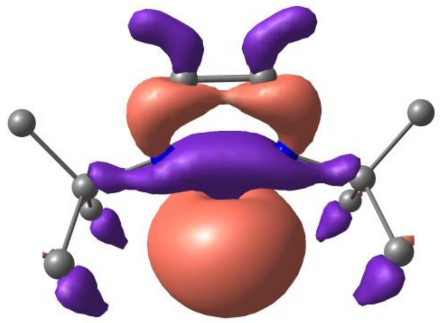

HOMO$_{ItBu}$

-5.67

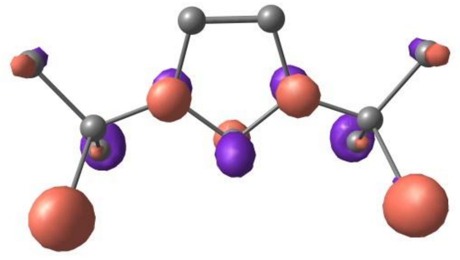

LUMO+3$_{ItBu}$

0.36

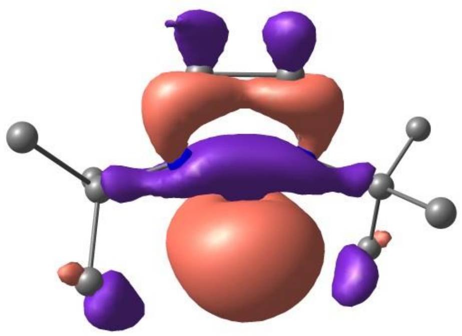

HOMO$_{SItBu}$

-5.46

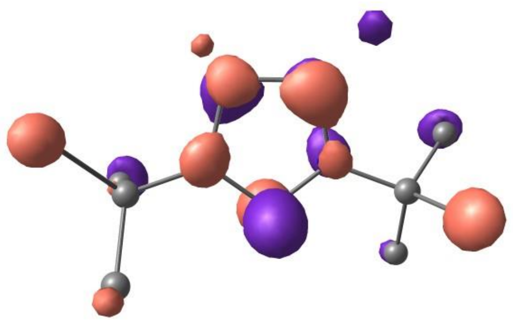

LUMO+2$_{SItBu}$

0.14

Table SI-10. %V_{bur} of M–NHCs Determined for M–(I*t*Oct) Complexes (Falivene, L. et al. *Nat. Chem.* 2019, *11*, 872)[a]

entry	compound	no	geometry	%V_{bur}
1	[Au(I*t*Oct)Cl]	**9**	Linear	44.7
2	[Cu(I*t*Oct)Cl]	**10**	Linear	48.2
3	[Ag(I*t*Oct)Cl]	**11**	Linear	43.7
4[b]	[Se(I*t*Oct)]	**12**	Linear	44.8
5[b]	[Se(I*t*Oct)]	**12**	Linear	44.1
6	[Pd(I*t*Oct)(3-py)Cl_2]	**14**	Square planar	38.4

[a]%V_{bur} determined from x-ray crystallography. [b]Two independent molecules were observed in the unit cell.

Additional Discussion. As expected, %V_{bur} determined for non-linear M–I*t*Oct complexes, such as square planar [Pd(I*t*Oct)(3-py)Cl_2] gives decreased %V_{bur} due to steric crowding around the metal center (*Chem. Commun.* **2017**, *53*, 2650). While square planar complexes are not suitable for accurate determination of %V_{bur}, the results presented in Table SI-4 indicate a major degree of flexibility of the N-wingtip substituents in M–I*t*Oct complexes, varying between %V_{bur} of 48.2% for [Cu(I*t*Oct)Cl] to %V_{bur} of 38.4% for [Pd(I*t*Oct)(3-py)Cl_2]. Optimal accommodation of steric demand is crucial to the catalytic performance of M–NHC complexes.

Table SI-11. Quadrant Distribution (%V_{bur}) of M–NHCs Determined for M–(I*t*Oct) Complexes (Falivene, L. et al. *Nat. Chem.* 2019, *11*, 872)[a]

entry	compound	SW	NW	NE	SE
1	[Au(I*t*Oct)Cl]	47.8	42.7	48.8	39.6
2	[Cu(I*t*Oct)Cl]	54.1	44.1	53.7	40.8
3	[Ag(I*t*Oct)Cl]	50.0	35.1	49.6	40.2
4[b]	[Se(I*t*Oct)]	51.8	38.7	49.8	38.7
5[b]	[Se(I*t*Oct)]	46.8	42.8	46.6	40.3
6	[Pd(I*t*Oct)(3-py)Cl_2]	34.0	41.8	36.7	41.0

[a]%V_{bur} determined from x-ray crystallography. [b]Two independent molecules were observed in the unit cell.

Chart SI-21. Topographical Steric Maps of M–(I*t*Oct) Complexes (Falivene, L. et al. *Nat. Chem.* 2019, *11*, 872)

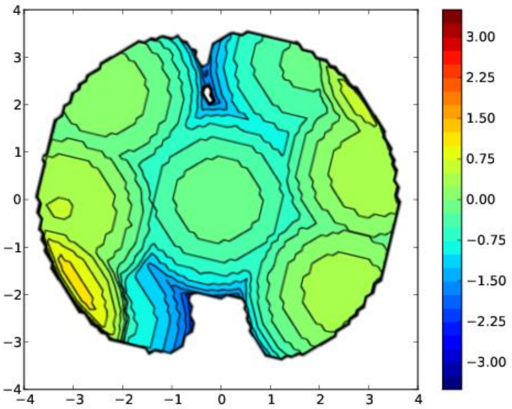

Au(I*t*Oct)Cl
%V*bur* = 44.7%

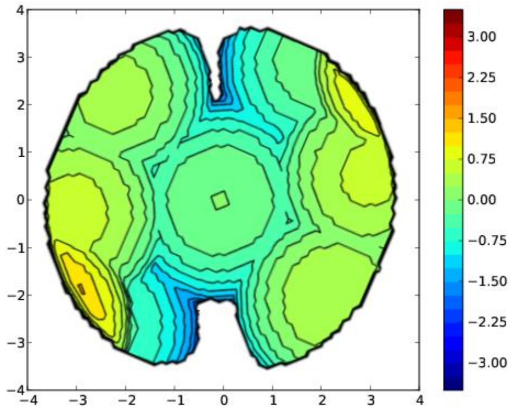

Cu(I*t*Oct)Cl
%V*bur* = 48.2%

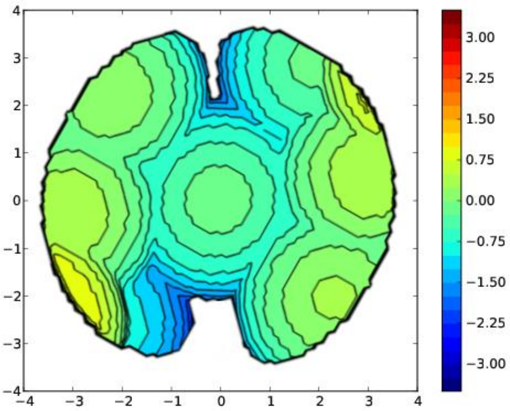

Ag(I*t*Oct)Cl
%V*bur* = 43.7%

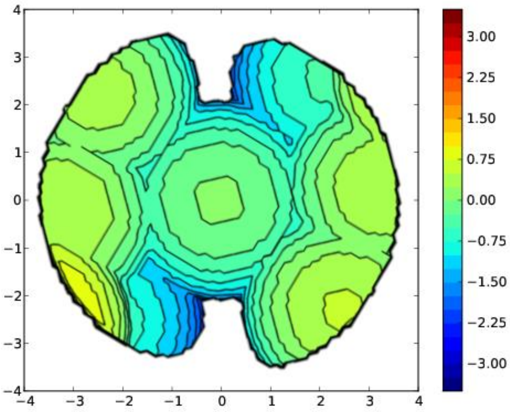

Se(I*t*Oct)-1
%V*bur* = 44.8%

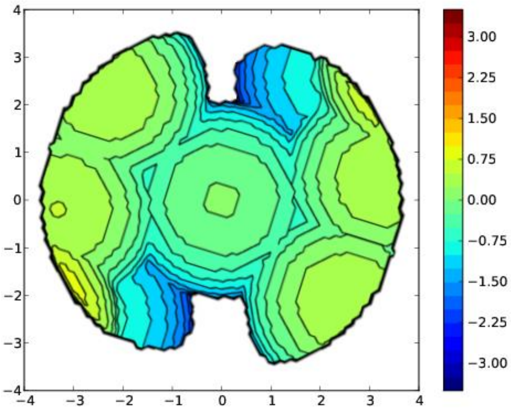

Se(I*t*Oct)-2
%V*bur* = 44.1%

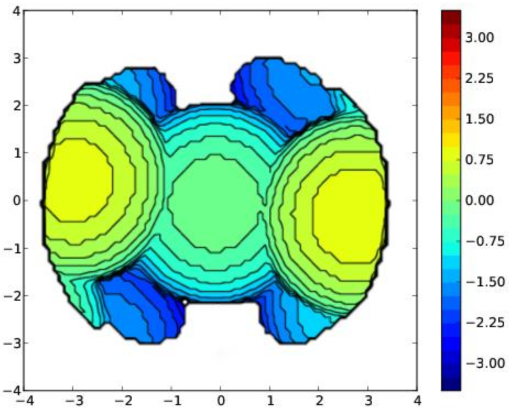

Pd(I*t*Oct)(3-py)Cl_2
%V*bur* = 38.4%

Se(ItOct) – two independent molecules in the unit cell.

Table SI-12. %V*bur* of M–NHCs Determined for Linear [Cu(NHC)Cl] Complexes at the B3LYP 6-311++g(d,p) Level (Falivene, L. et al. *Nat. Chem.* 2019, *11*, 872)[a]

entry	compound	no	geometry	%V*bur*
1	[Cu(I*t*Oct)Cl]	**9**	Linear	45.1
2	[Cu(SI*t*Oct)Cl]	**9-SI**	Linear	47.1
3	[Cu(I*t*Bu)Cl]	**18b**	Linear	41.0
4	[Cu(SI*t*Bu)]	**18b-SI**	Linear	41.7

[a]See Table SI-8 for bond lengths and angles.

Additional Discussion. We have determined the percent buried volume for the linear [Cu(NHC)Cl] complexes (NHC = I*t*Oct, I*t*Bu, SI*t*Oct, SI*t*Bu) calculated at the B3LYP 6-311++g(d,p) level. The accurate determination of the computed linear geometry obviates any effects from crystal packing. This approach is particularly useful in comparing a series of compounds. Linear Cu(I)–NHC complexes were selected to facilitate computations. This method permits to readily compare I*t*Oct and SI*t*Oct with I*t*Bu and SI*t*Bu ligands (Tables SI-12, SI-13 and Chart SI-22)

The striking difference in the steric environment between I*t*Oct and I*t*Bu should be noted. There are two major steric effects of the I*t*Bu to I*t*Oct replacement: (1) increased percent buried volume, resulting in a more sterically-demanding ligand; (2) non-equivalent quadrant distribution in steric maps. The same differnce is observed in the comparison between SI*t*Oct and SI*t*Bu. Overall, these studies strongly support I*t*Oct as a unique class of N-alkyl ligands with sterically-defined C_2-symmetric environment and strong σ-donation enabled by N-alkyl wingtip substitution. Further studies on the development of NHC ligands are underway in our laboratory and these findings will be reported in due course.

Table SI-13. Quadrant Distribution (%V$_{bur}$) of M–NHCs Determined for Linear [Cu(NHC)Cl] Complexes at the B3LYP 6-311++g(d,p) Level (Falivene, L. et al. *Nat. Chem.* 2019, *11*, 872)[a]

entry	compound	SW	NW	NE	SE
1	[Cu(I*t*Oct)Cl]	49.1	41.2	49.1	41.2
2	[Cu(SI*t*Oct)Cl]	50.9	43.4	50.9	43.4
3	[Cu(I*t*Bu)Cl]	41.0	41.0	41.0	41.0
4	[Cu(SI*t*Bu)]	41.5	42.0	41.5	42.0

[a]See Table SI-8 for bond lengths and angles.

Chart SI-22. Topographical Steric Maps of [Cu(NHC)Cl] Complexes at the B3LYP 6-311++g(d,p) Level (Falivene, L. et al. *Nat. Chem.* 2019, *11*, 872)

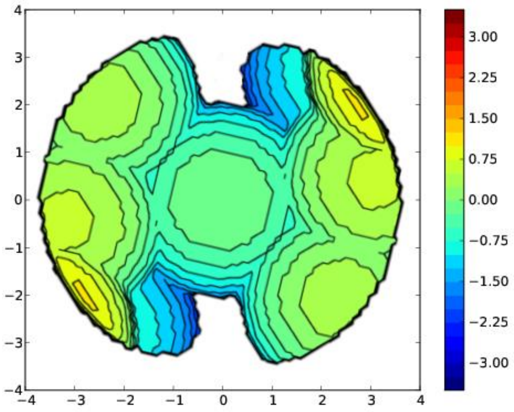

Cu(I*t*Oct)Cl
%V*bur* = 45.1%

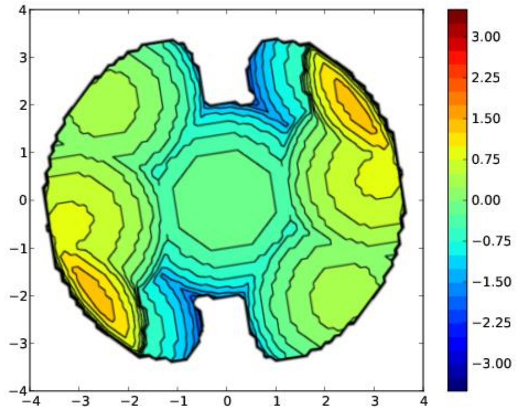

Cu(SI*t*Oct)Cl
%V*bur* = 47.1%

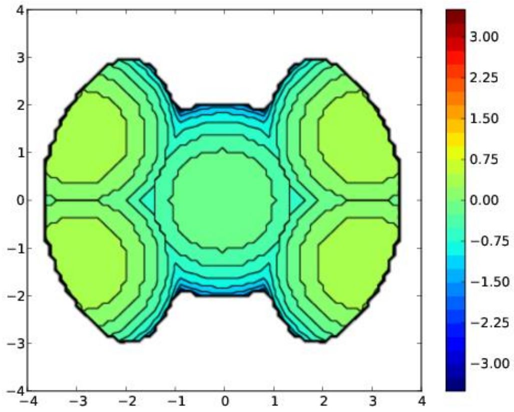

Cu(I*t*Bu)Cl
%V*bur* = 41.0%

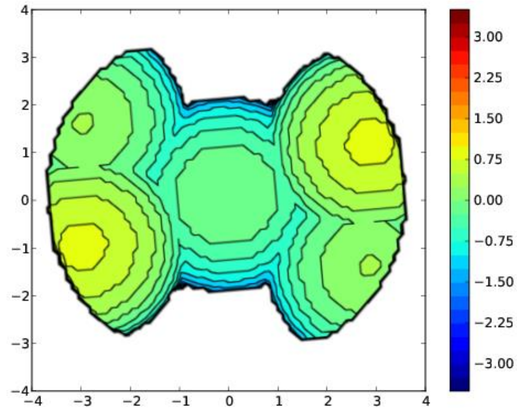

Cu(SI*t*Bu)
%V*bur* = 41.7%

Note the striking difference is the steric environment between the I*t*Oct and I*t*Bu ligands (see also Tables SI-12 and SI-13). Sterically-demanding I*t*OCt leads to a much higher %V*bur* and non-equivalent quadrant distribution (see also SI*t*Oct vs. SI*t*Bu).

Plot of %V_{bur} vs. Charton parameter in linear [Au(NHC)Cl] complexes

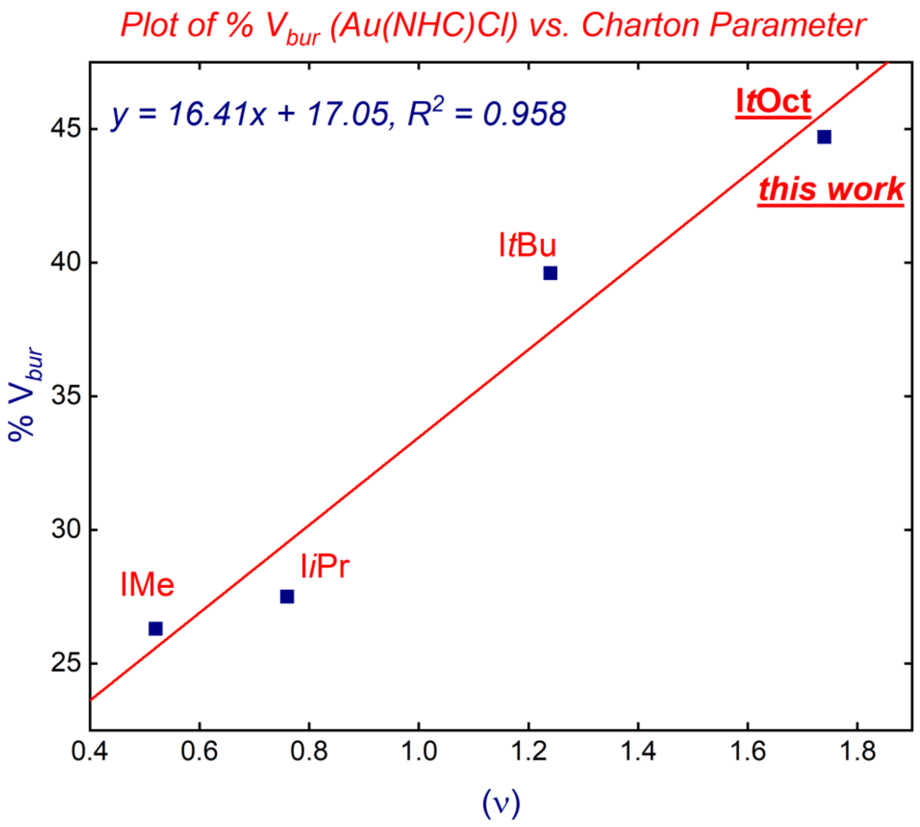

Chart SI-23. Plot of %V_{bur} vs. Charton parameter in linear [Au(NHC)Cl] complexes. %V_{bur} values for IMe (IDM), I*i*Pr, I*t*Bu have been reported (*Chem. Commun.* **2017**, *53*, 2650). Note that IAd is sterically comparable to I*t*Bu (IAd: %V_{bur} = 39.8%, ν = 1.33; ItBu: %V_{bur} = 39.6%, ν = 1.24).

References

1. Scattolin, T.; Tzouras, N. V.; Falivene, L.; Cavallo, L.; Nolan, S. P. Using sodium acetate for the synthesis of [Au(NHC)X] complexes. *Dalton Trans.* **2020**, *49*, 9694–9700.
2. Diez-Gonzalez, S.; Escudero-Adan, E. C.; Benet-Buchholz, J.; Stevens, E. D.; Slawin, A. M. Z.; Nolan, S. P. [(NHC)CuX] complexes: Synthesis, characterization and catalytic activities in reduction reactions and click chemistry. On the advantage of using well-defined catalytic systems. *Dalton Trans.* **2010**, *39*, 7595–7606.
3. Sgro, M. J.; Piers, W. E.; Romero, P. E. Synthesis, structural characterization and thermal properties of copper and silver silyl complexes. *Dalton Trans.* **2015**, *44*, 3817–3828.
4. Rahman, M. M.; Buchspies, J.; Szostak, M. N-Acylphthalimides: Efficient acyl coupling reagents in Suzuki–Miyaura cross-coupling by N–C cleavage catalyzed by Pd–PEPPSI precatalysts. *Catalysts* **2019**, *9*, 129–139.
5. Marion, N.; Ramon, R. S.; Nolan, S. P. [(NHC)Au^{I}]-Catalyzed acid-free alkyne hydration at part-per-million catalyst loadings. *J. Am. Chem. Soc.* **2009**, *131*, 448–449.
6. Brzozowska, A.; Zubar, V.; Ganardi, R. C.; Rueping, M. Chemoselective hydroboration of propargylic alcohols and amines using a manganese(II) catalyst. *Org. Lett.* **2020**, *22*, 3765−3769.
7. Bidal, Y. D.; Lazreg, F.; Cazin, C. S. J. Copper-catalyzed regioselective formation of tri- and tetrasubstituted vinylboronates in air. *ACS Catal.* **2014**, *4*, 1564−1569.
8. Paul, S.; Joy, B. P.; Rajendran, R.; Gudimetla, V. B. Cost efficient synthesis of diaryl ethers catalysed by CuI, imidazolium chloride and Cs_2CO_3. *ChemistrySelect* **2019**, *4*, 7181–7186.
9. Zhou, T.; Xie, P. P.; Ji, C. L.; Hong, X.; Szostak. M. Decarbonylative Suzuki−Miyaura Cross-Coupling of Aroyl Chlorides. *Org. Lett.* **2020**, *22*, 6434−6440.
10. Shi, S; Meng, G.; Szostak, M. Synthesis of cbiaryls through nickel-catalyzed Suzuki–Miyaura coupling of amides by carbon–nitrogen bond cleavage. *Angew. Chem. Int. Ed.* **2016**, *55*, 6959–6963.
11. Viciu, M. S.; Kissling, R. M.; Stevens, E. D.; Nolan, S. P. An air-stable palladium/*N*-heterocyclic carbene complex and its reactivity in aryl amination. *Org. Lett.* **2002**, *4*, 2229–2231.

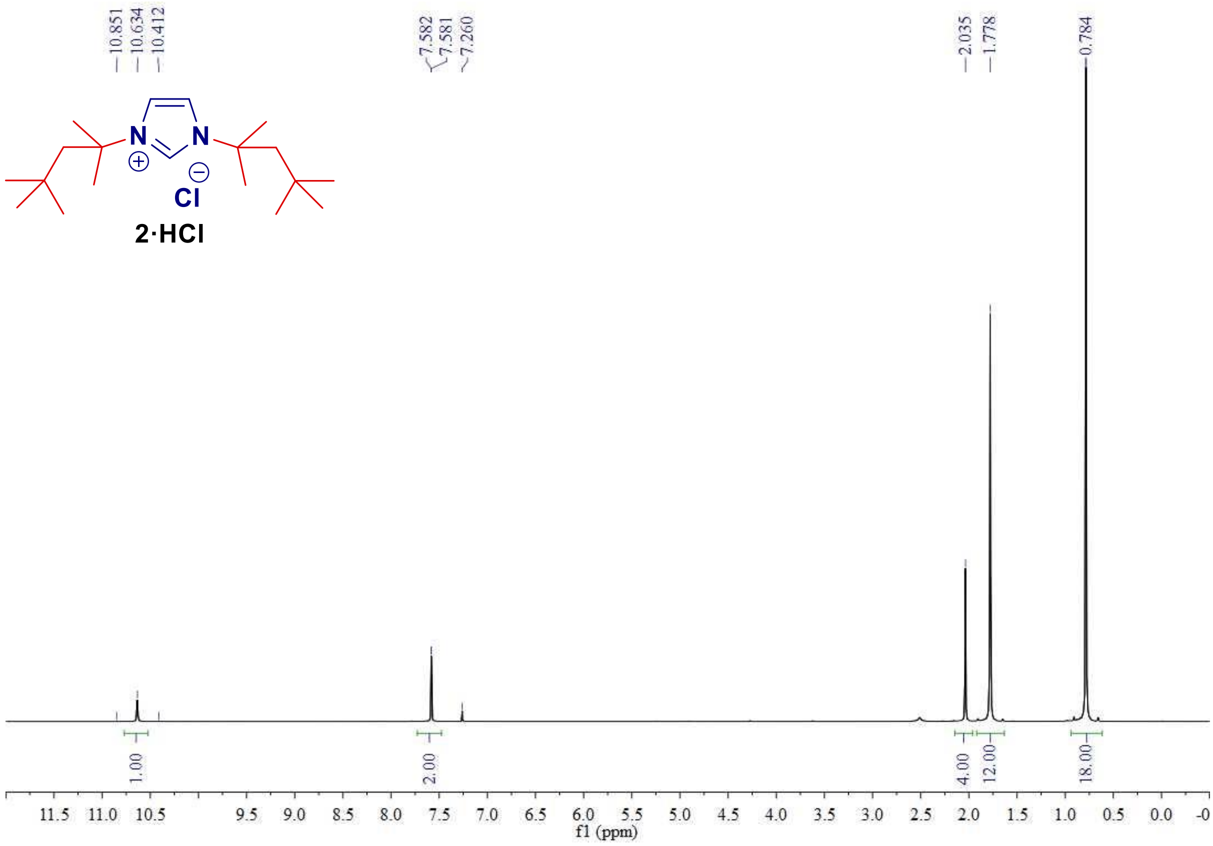

^{1}H NMR (500 MHz, $CDCl_3$) Spectrum of Compound **2·HCl**

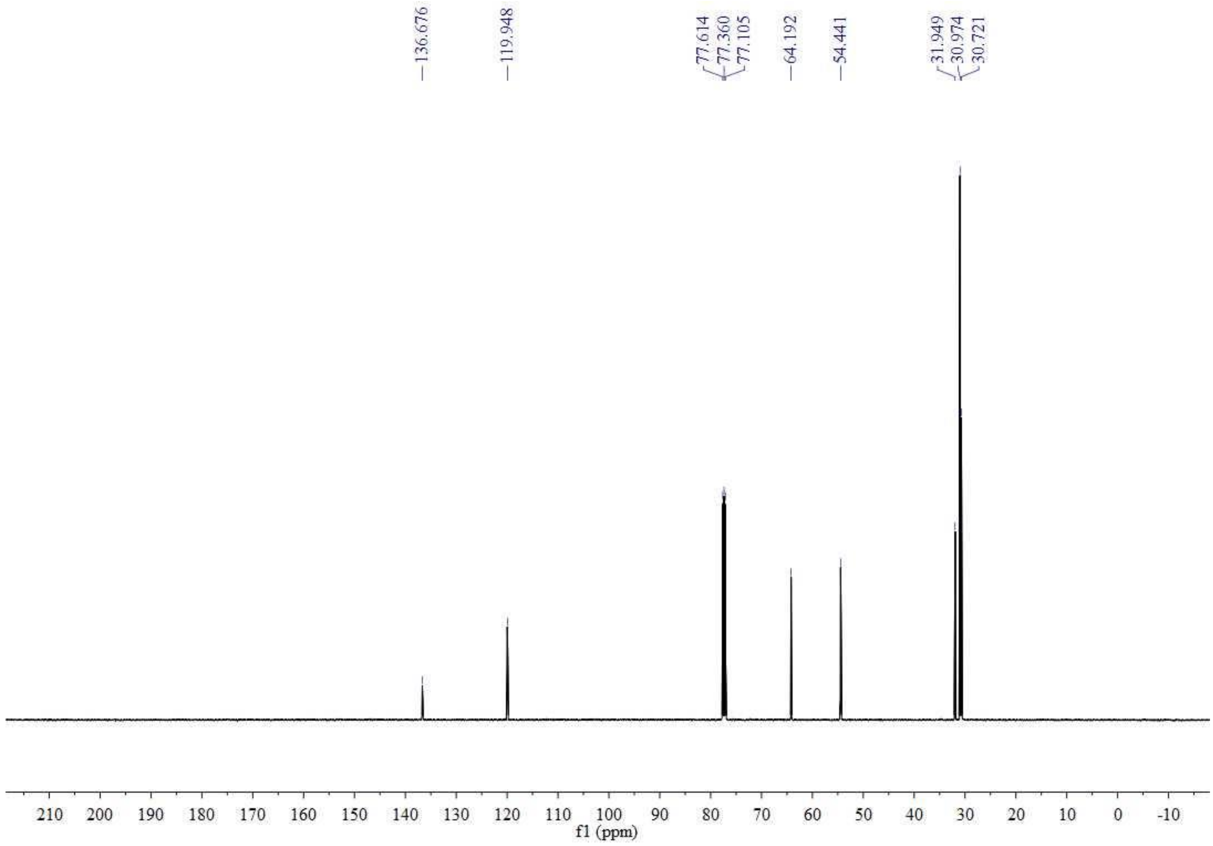

^{13}C NMR (126 MHz, $CDCl_3$) Spectrum of Compound **2·HCl**

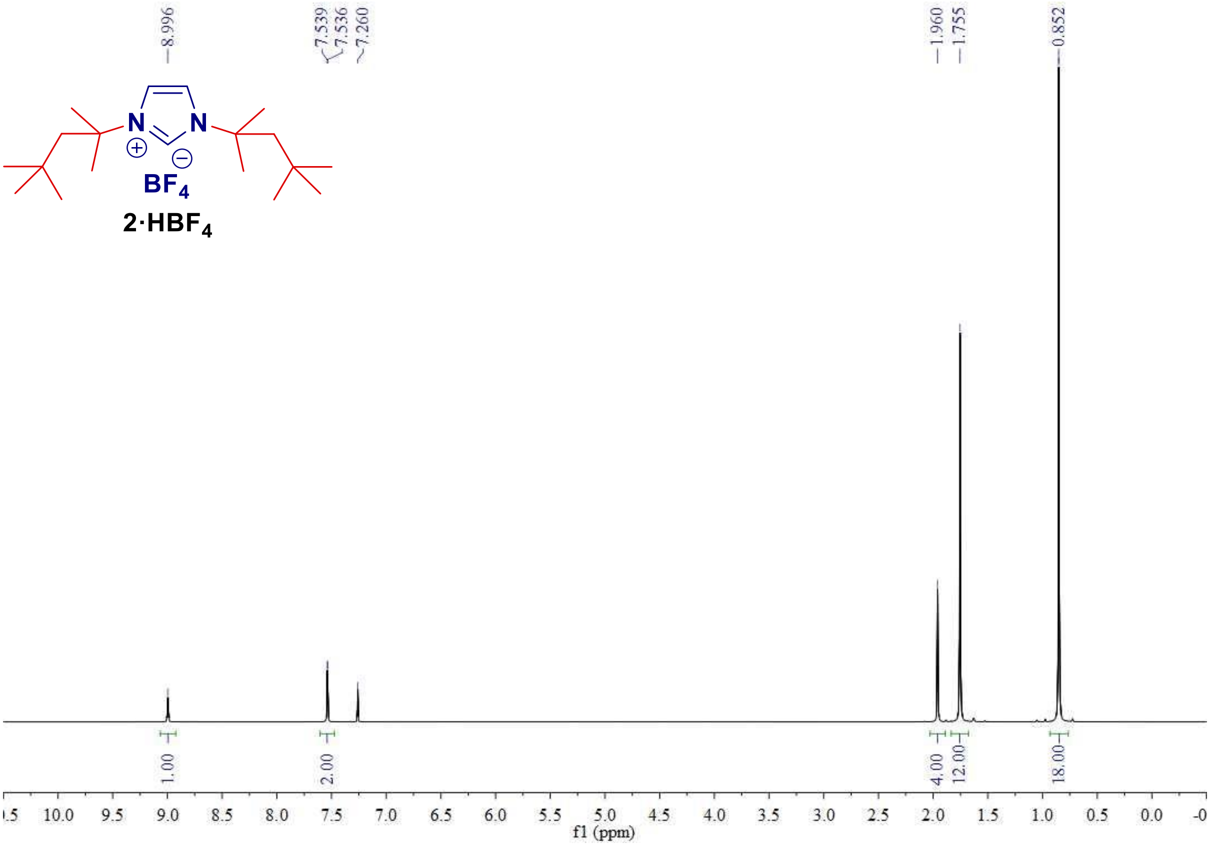

^{1}H NMR (500 MHz, $CDCl_3$) Spectrum of Compound **2·HBF₄**

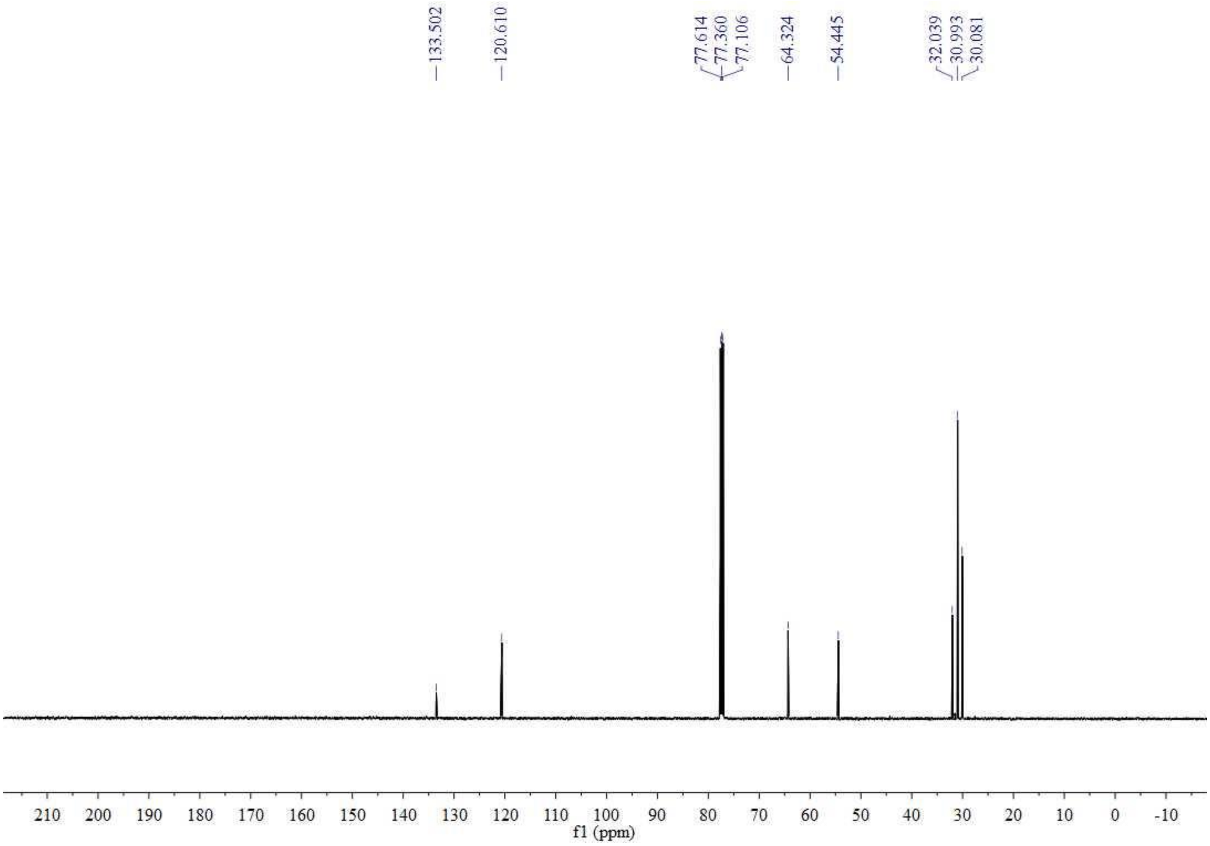

^{13}C NMR (126 MHz, $CDCl_3$) Spectrum of Compound **2·HBF₄**

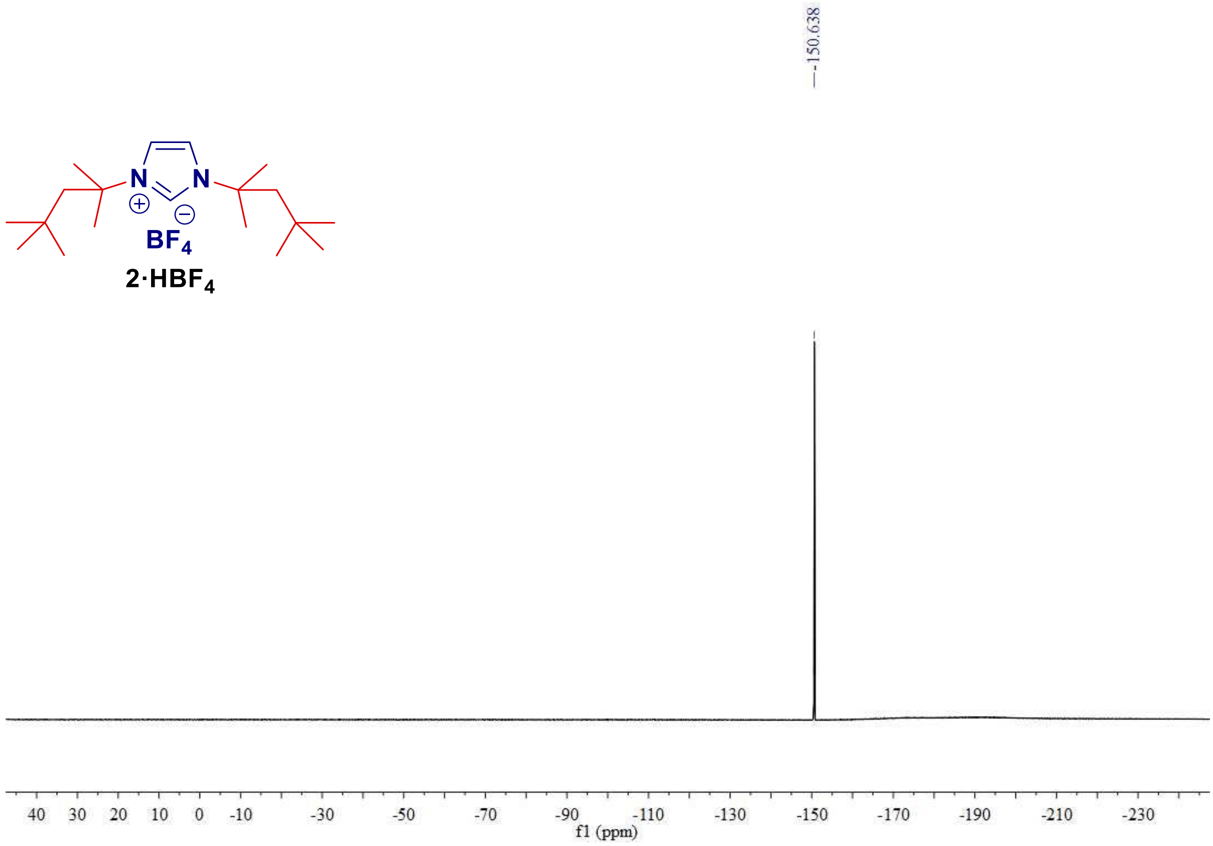

^{19}F NMR (471 MHz, $CDCl_3$) Spectrum of Compound **2·HBF4**

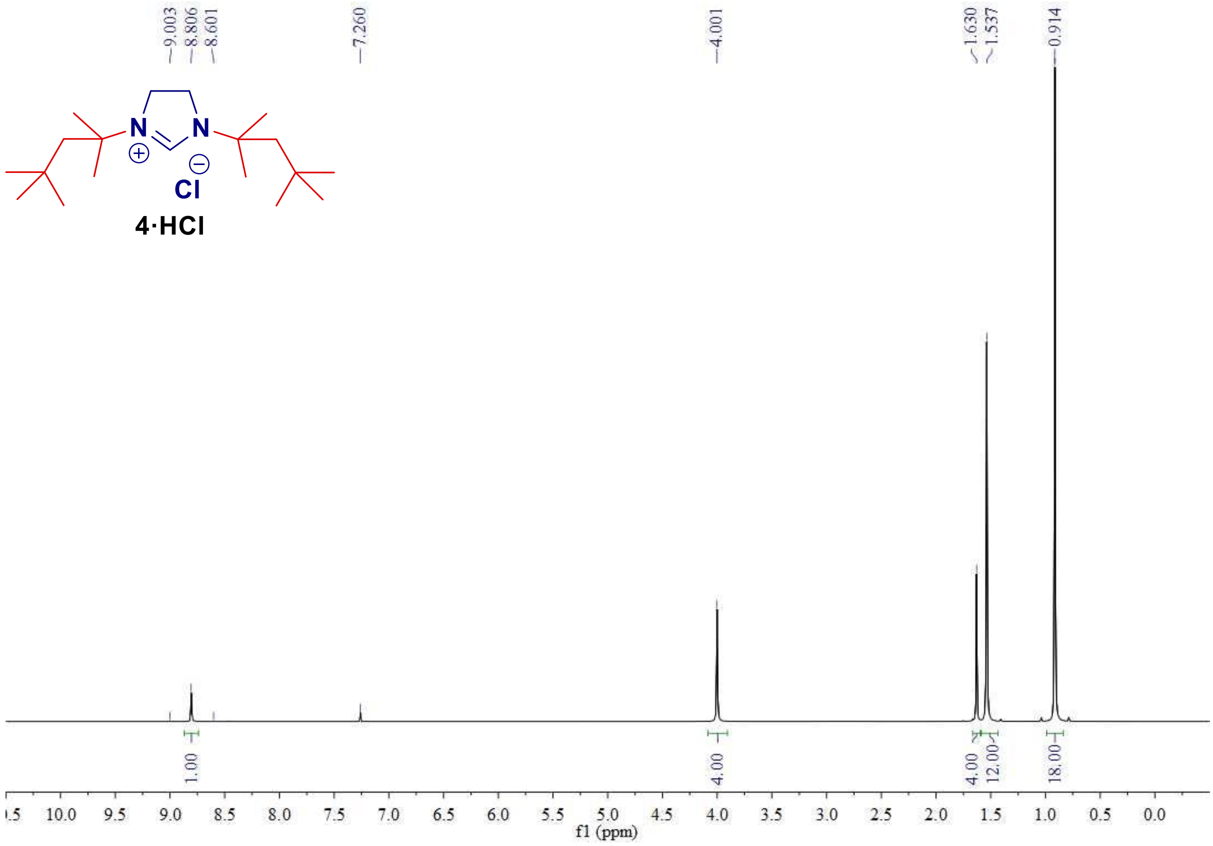

^{1}H NMR (500 MHz, $CDCl_3$) Spectrum of Compound **4·HCl**

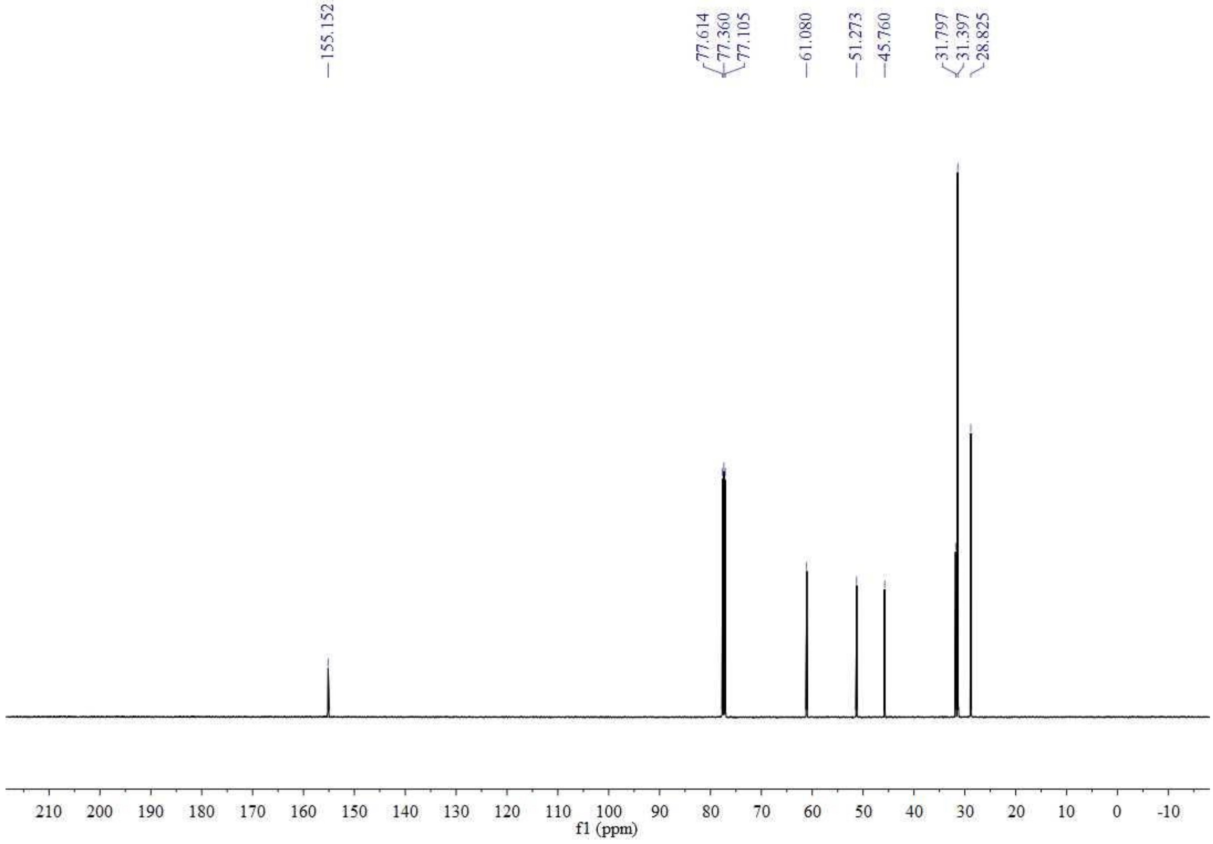

^{13}C NMR (126 MHz, $CDCl_3$) Spectrum of Compound **4·HCl**

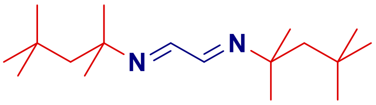

7

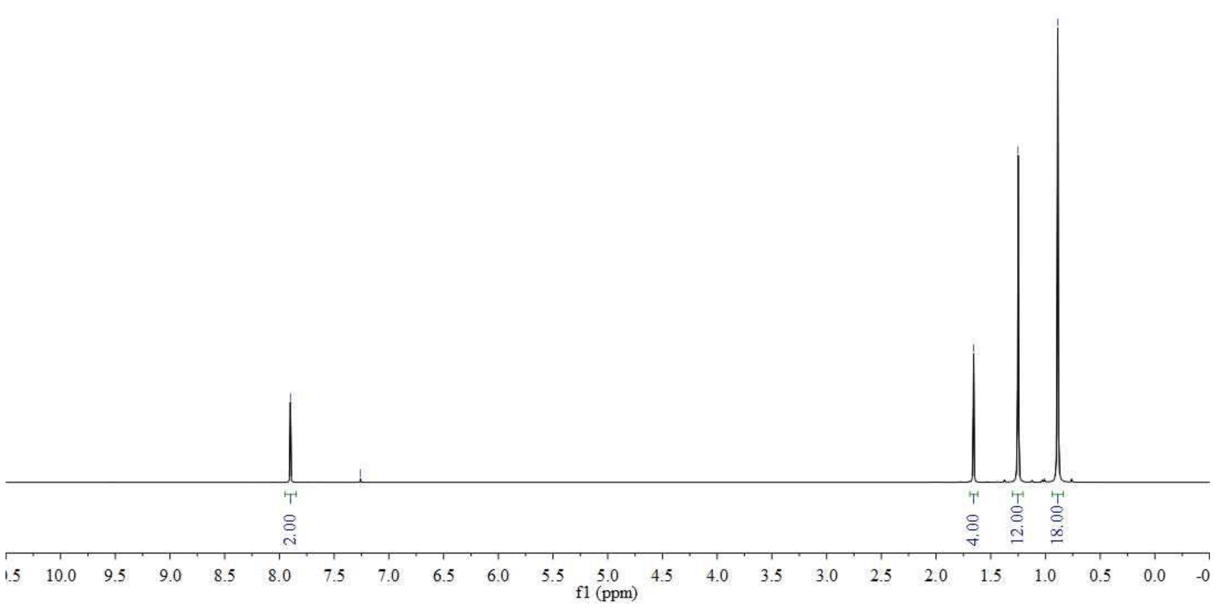

^{1}H NMR (500 MHz, CDCl$_3$) Spectrum of Compound **7**

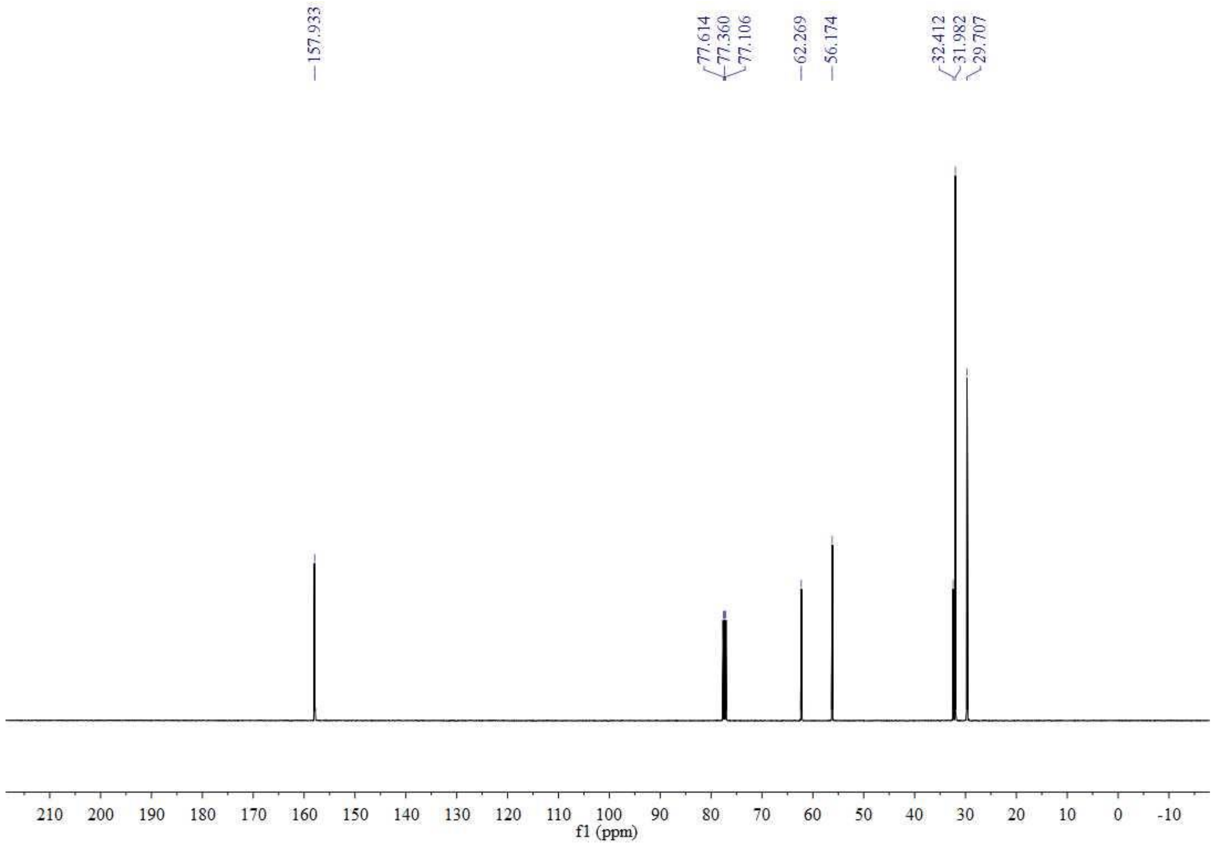

^{13}C NMR (126 MHz, CDCl$_3$) Spectrum of Compound **7**

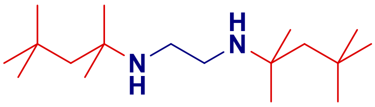

8

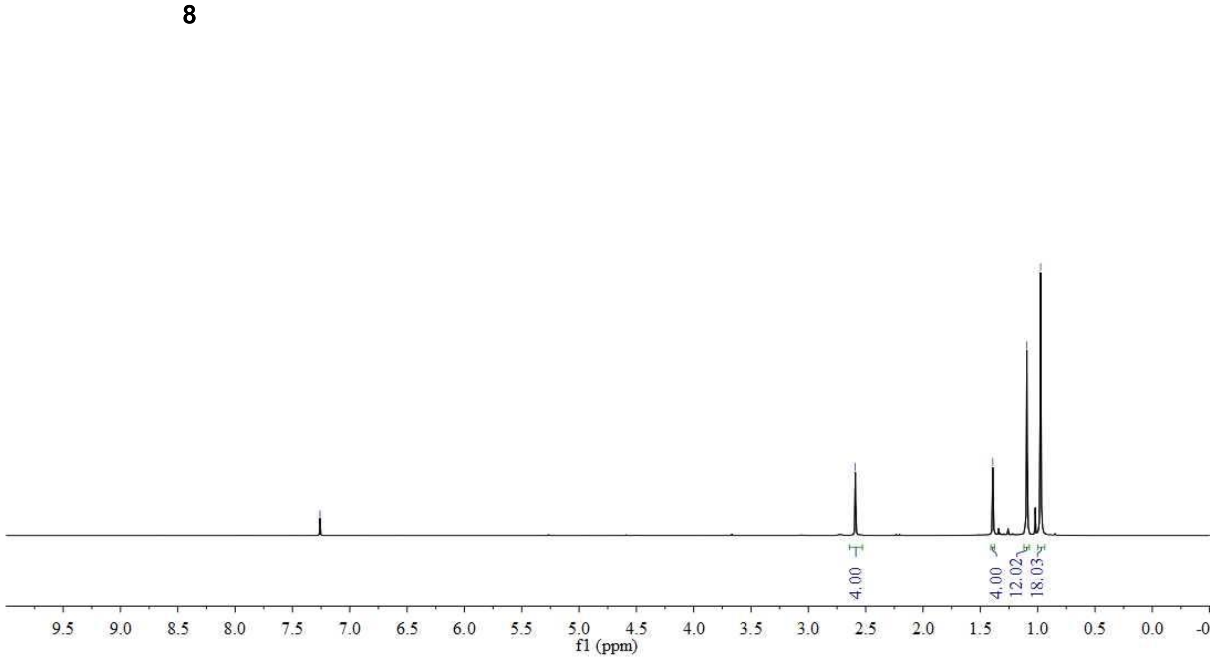

^{1}H NMR (500 MHz, $CDCl_3$) Spectrum of Compound **8**

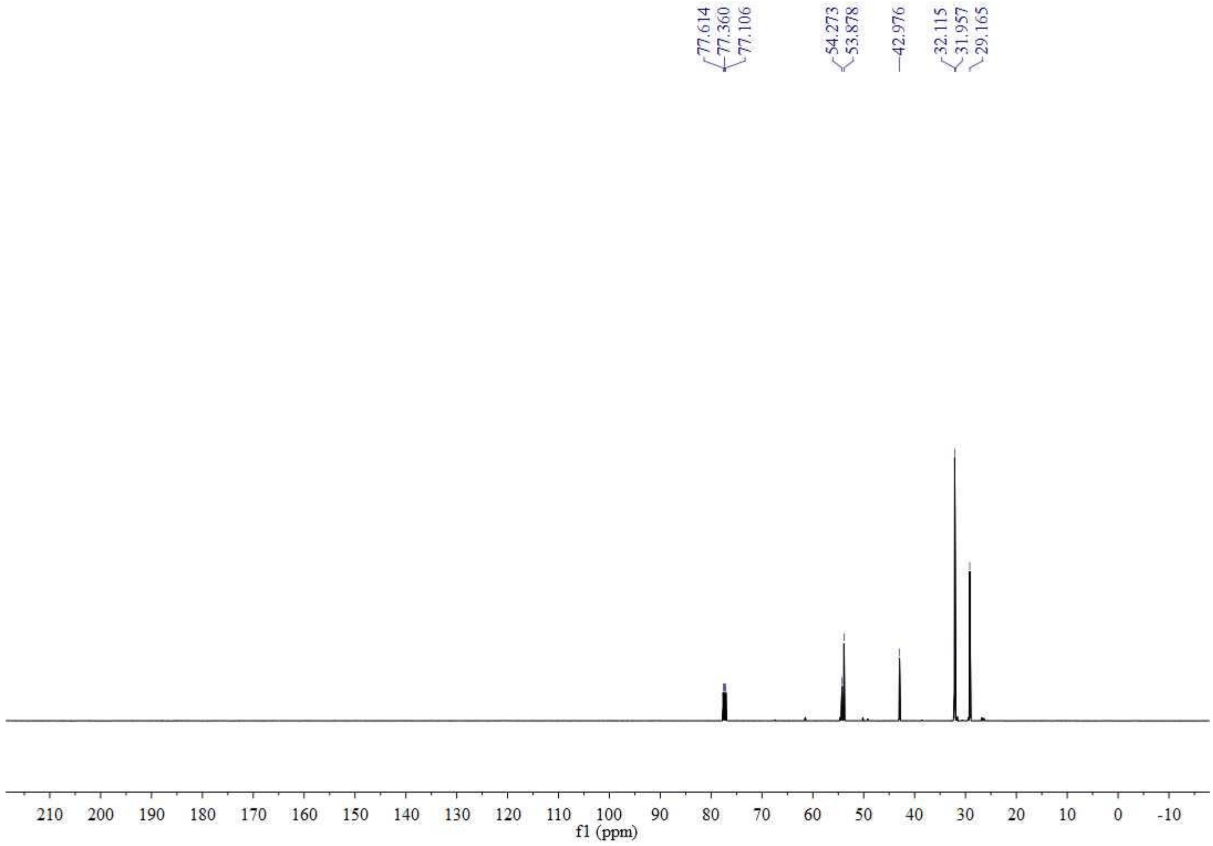

^{13}C NMR (126 MHz, $CDCl_3$) Spectrum of Compound **8**

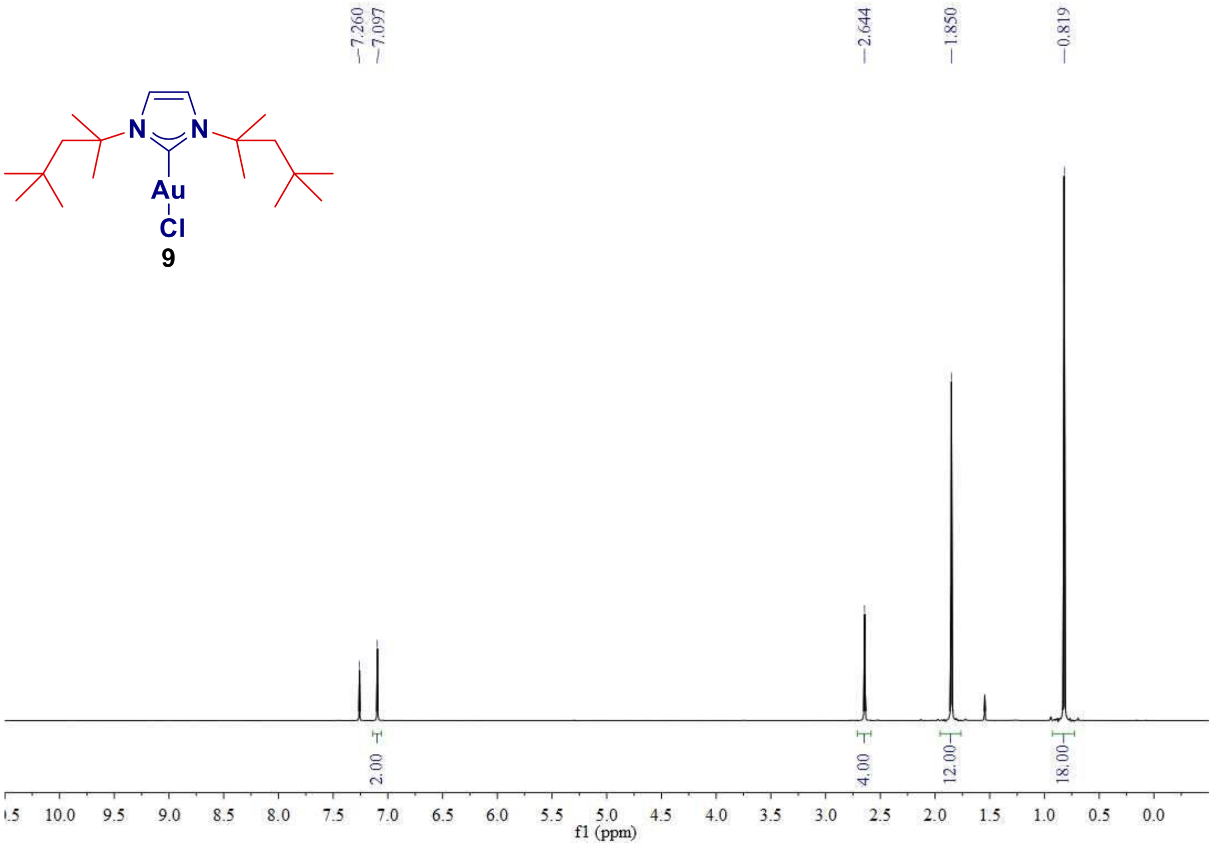

^{1}H NMR (500 MHz, $CDCl_3$) Spectrum of Compound **9**

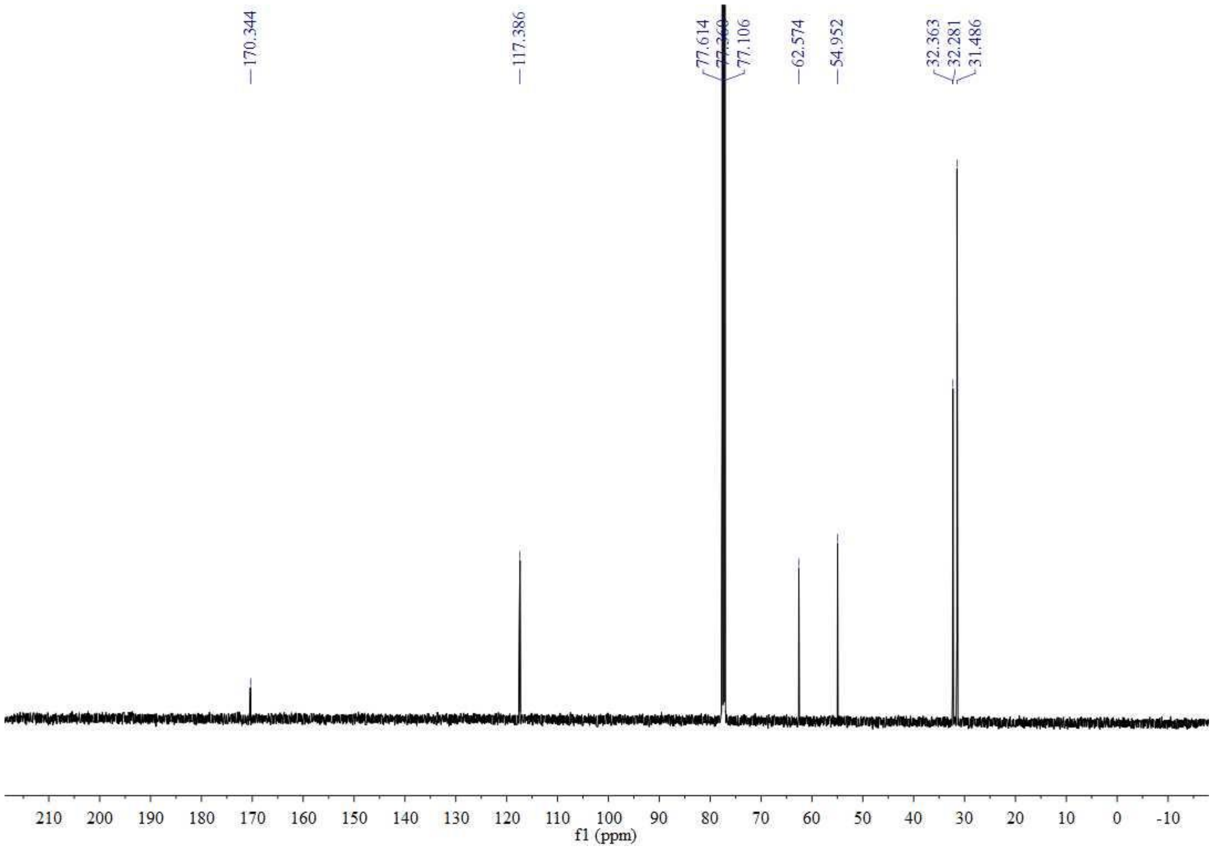

^{13}C NMR (126 MHz, $CDCl_3$) Spectrum of Compound **9**

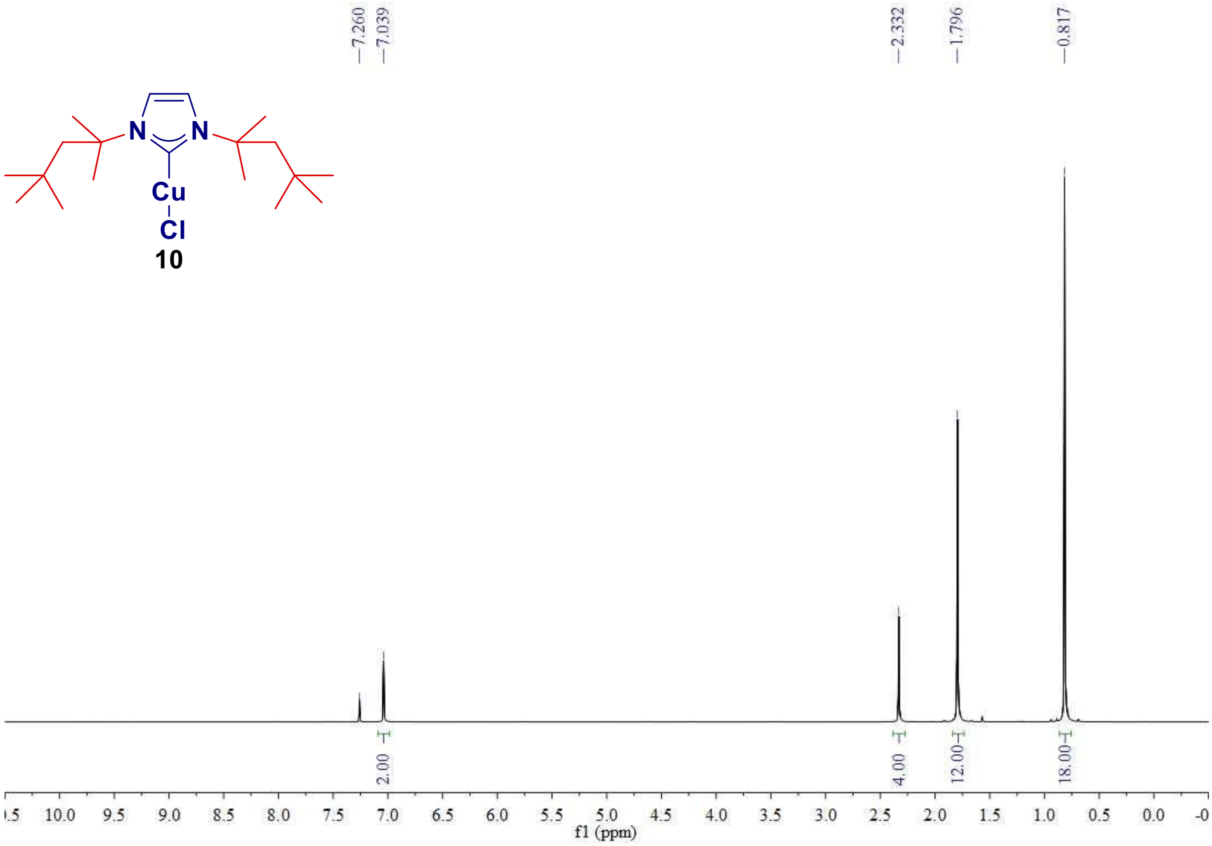

^{1}H NMR (500 MHz, $CDCl_3$) Spectrum of Compound **10**

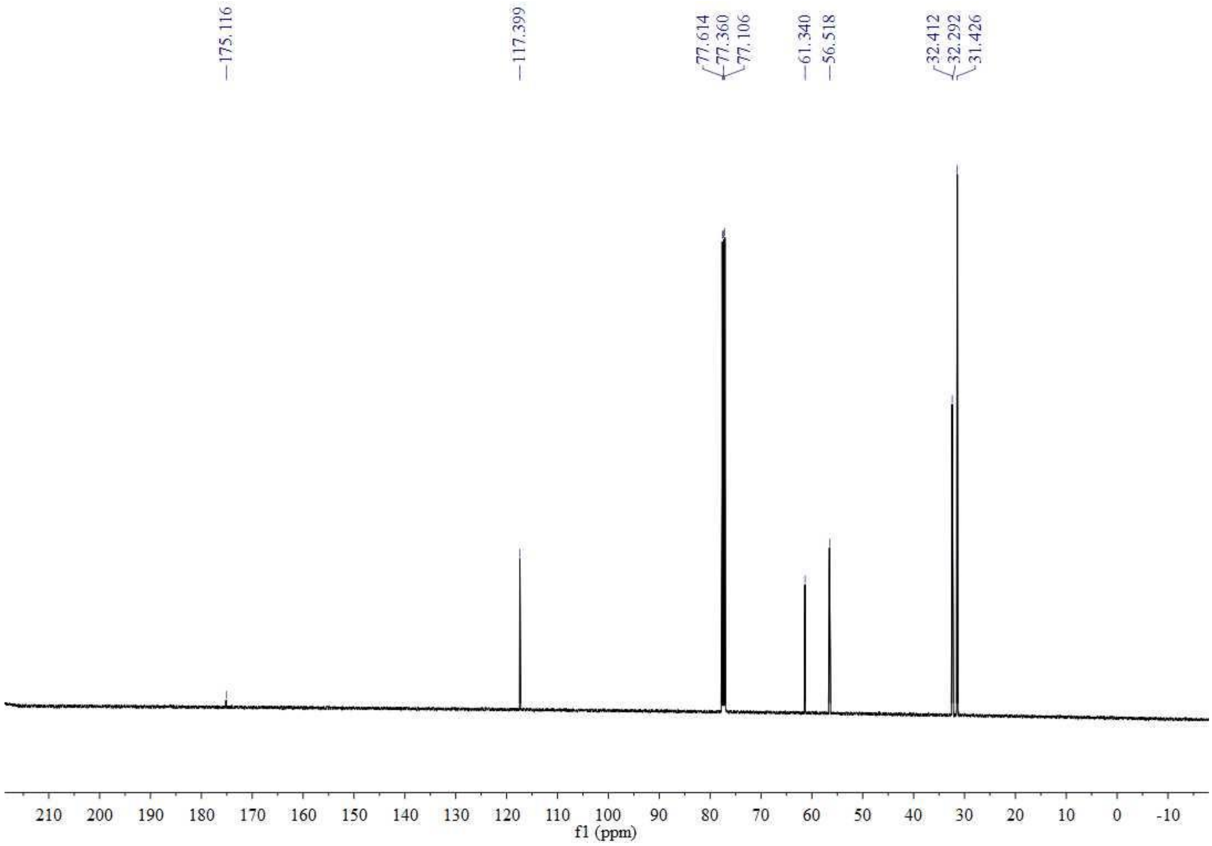

^{13}C NMR (126 MHz, $CDCl_3$) Spectrum of Compound **10**

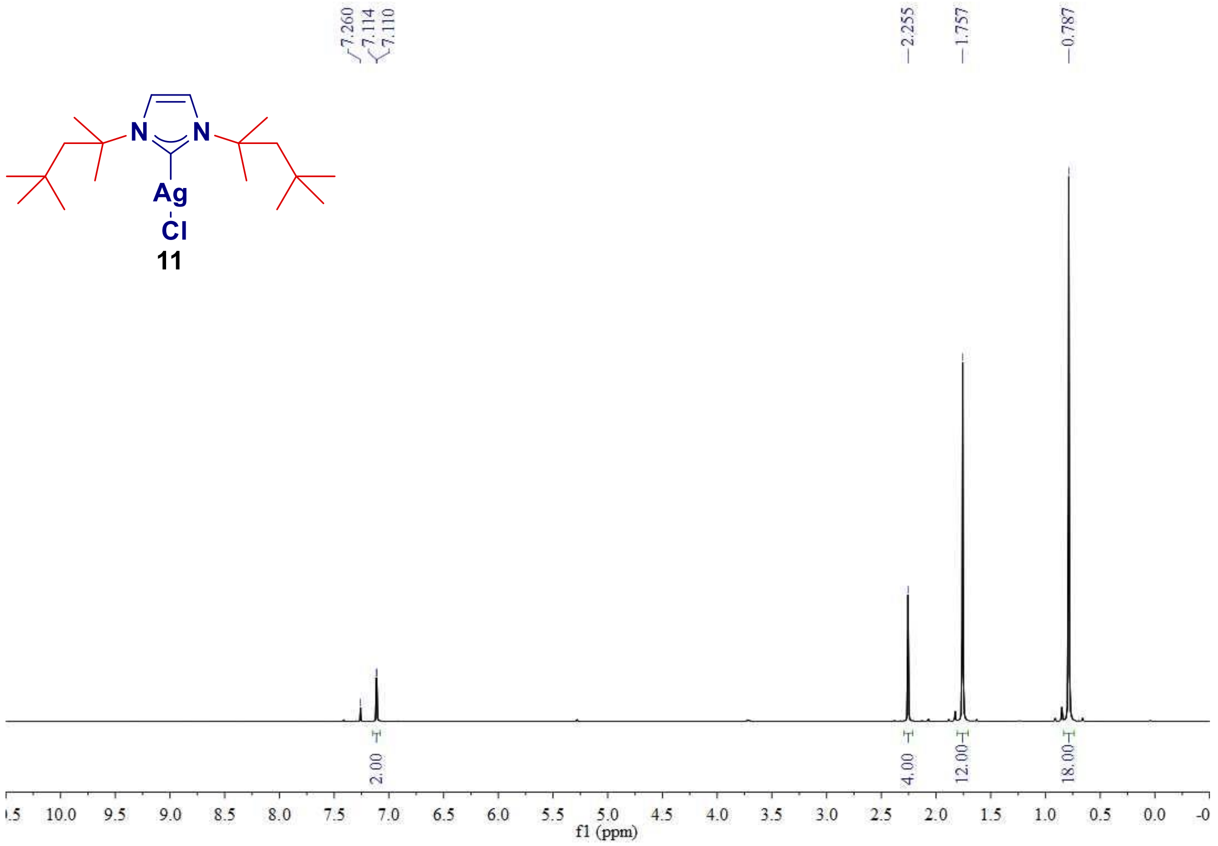

^{1}H NMR (500 MHz, $CDCl_3$) Spectrum of Compound **11**

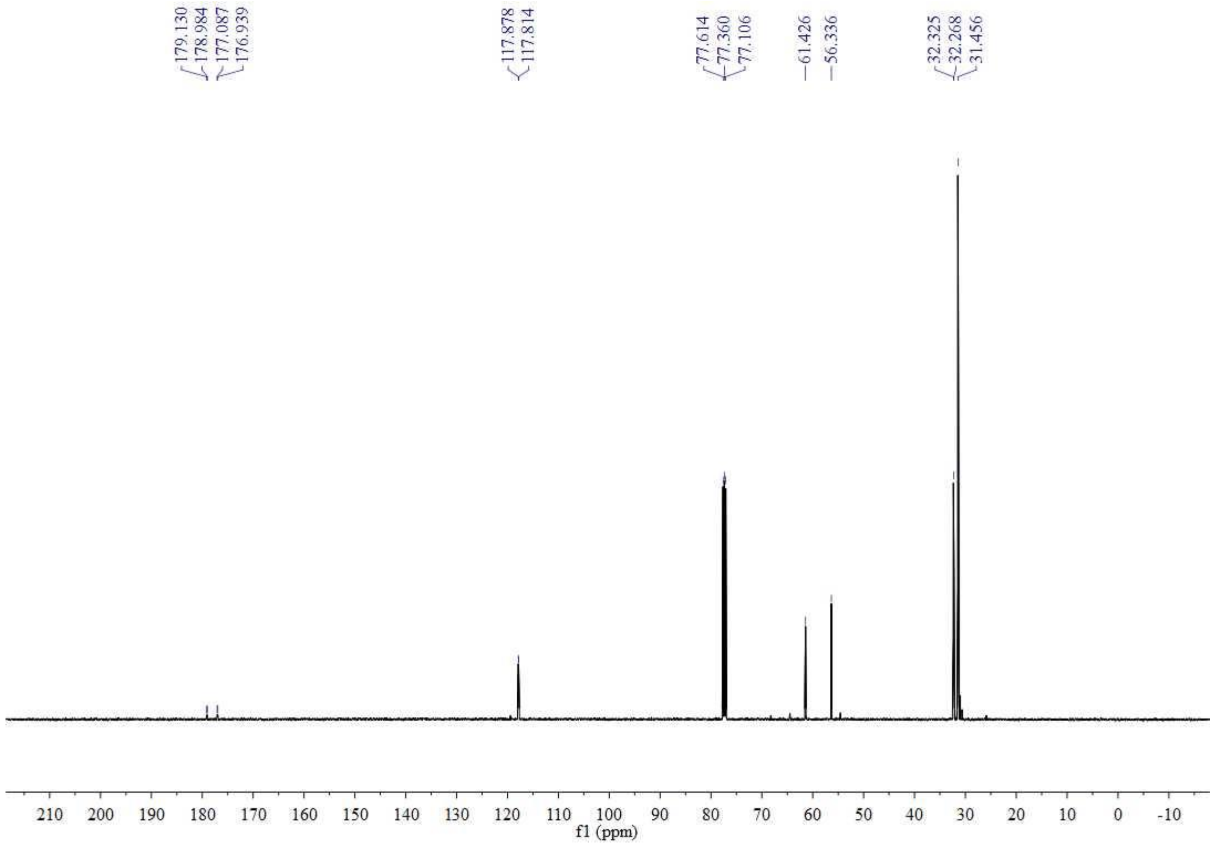

^{13}C NMR (126 MHz, $CDCl_3$) Spectrum of Compound **11**

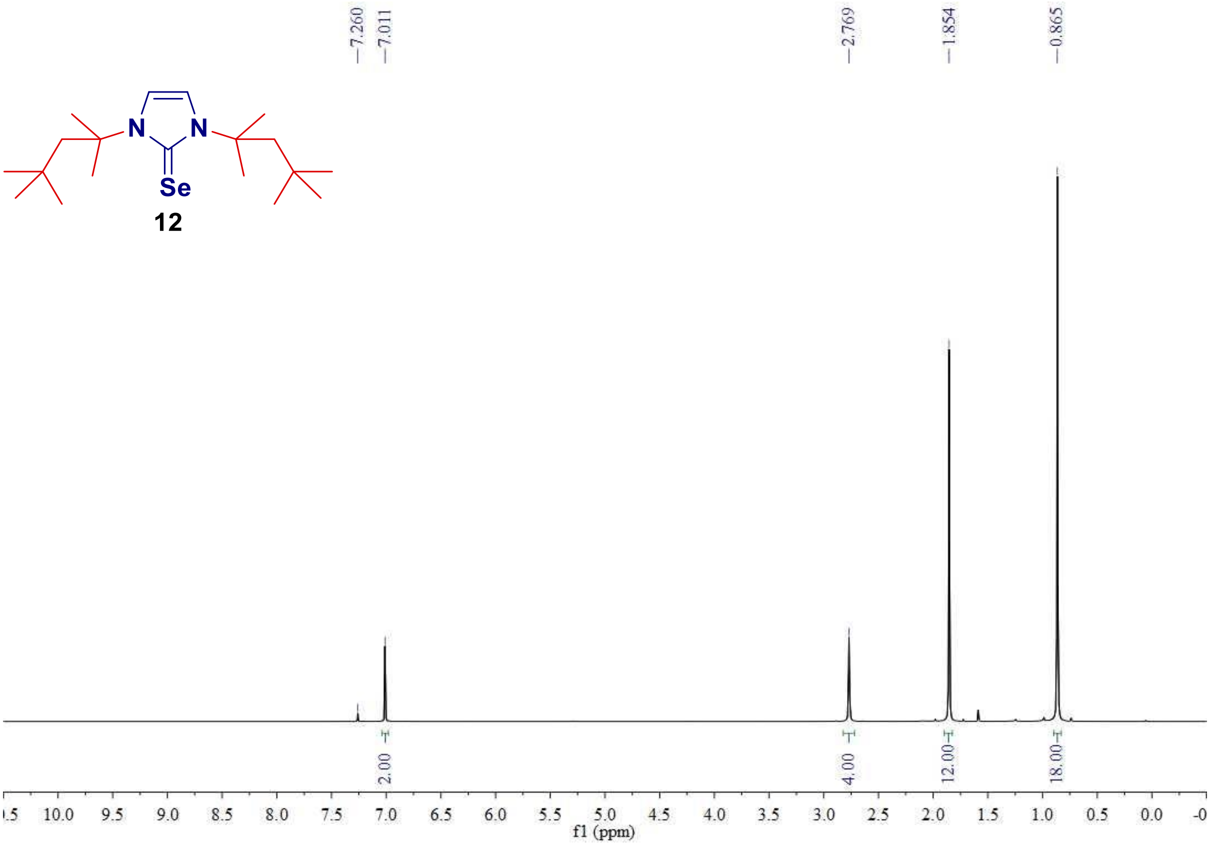

^{1}H NMR (500 MHz, $CDCl_3$) Spectrum of Compound **12**

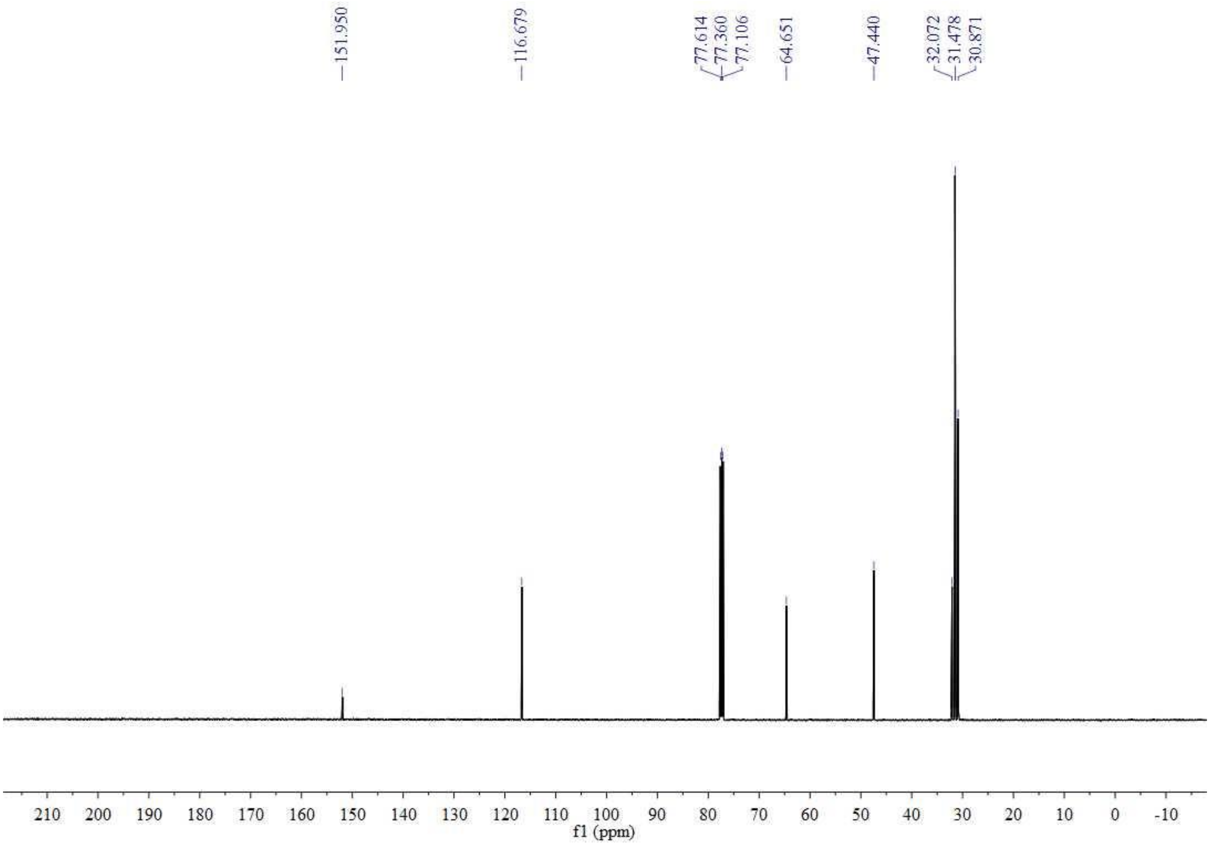

^{13}C NMR (126 MHz, $CDCl_3$) Spectrum of Compound **12**

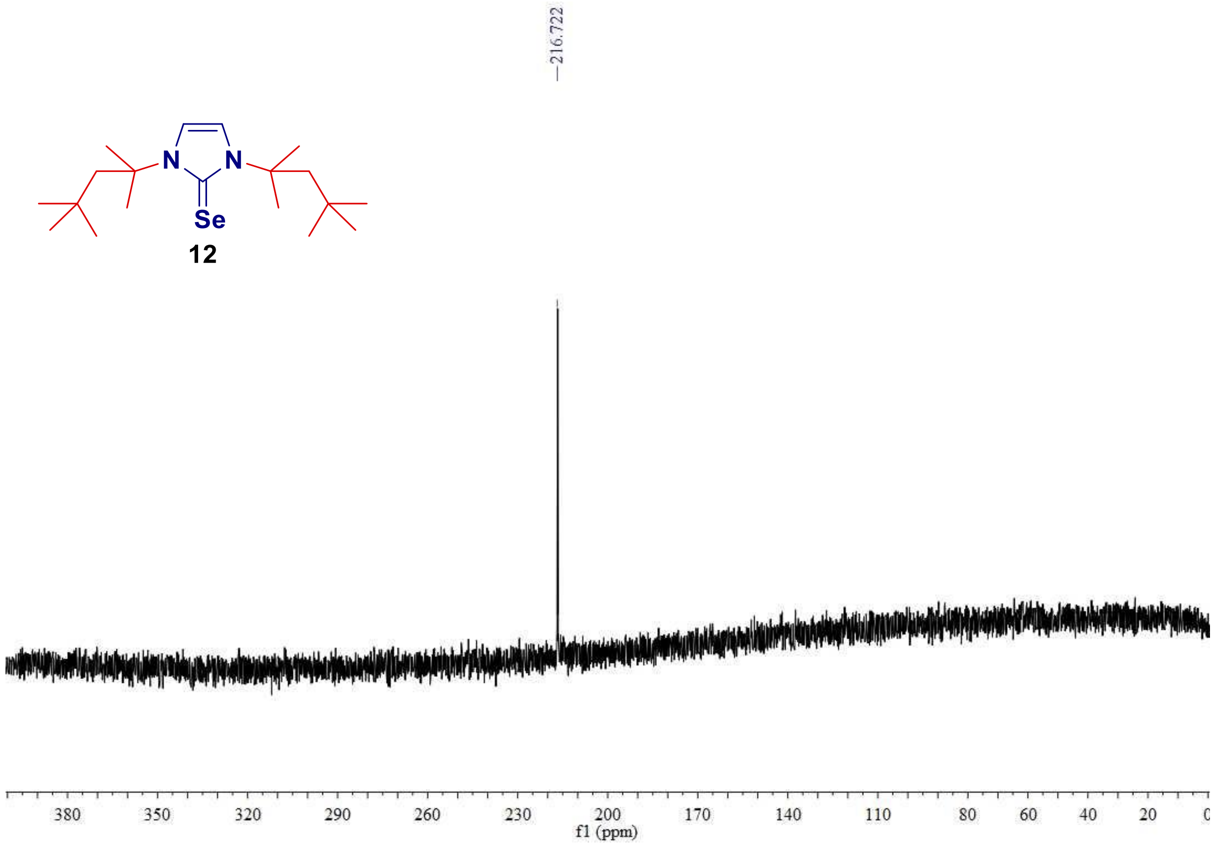

^{77}Se NMR (95 MHz, $CDCl_3$) Spectrum of Compound **12**

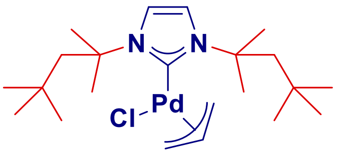

13

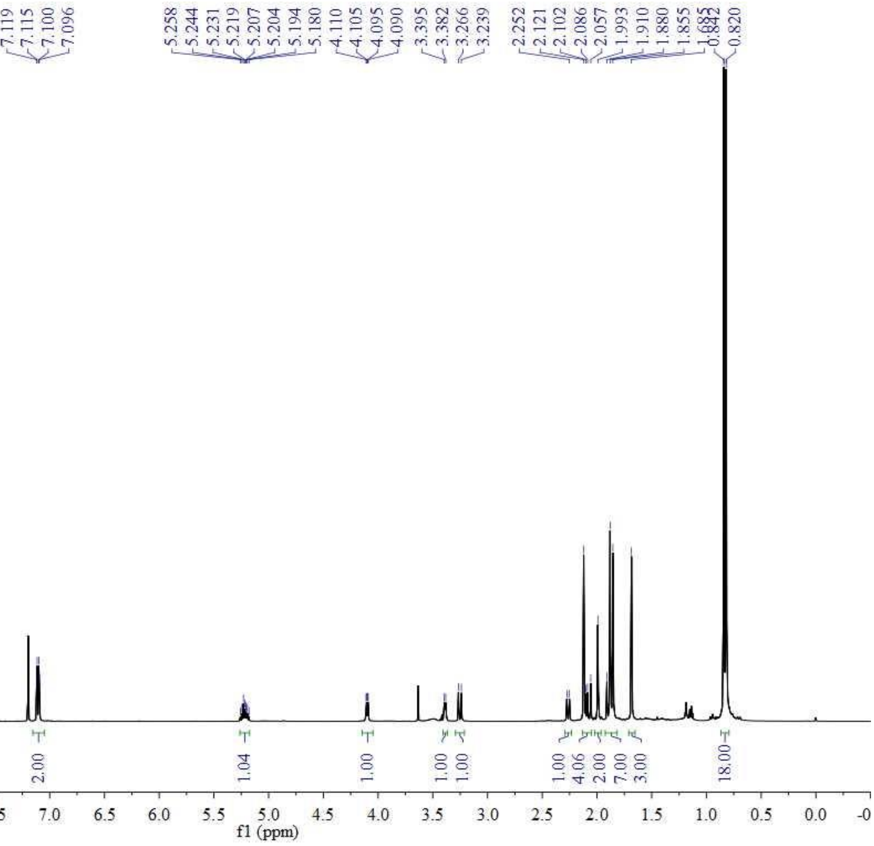

^{1}H NMR (500 MHz, $CDCl_3$) Spectrum of Compound **13**

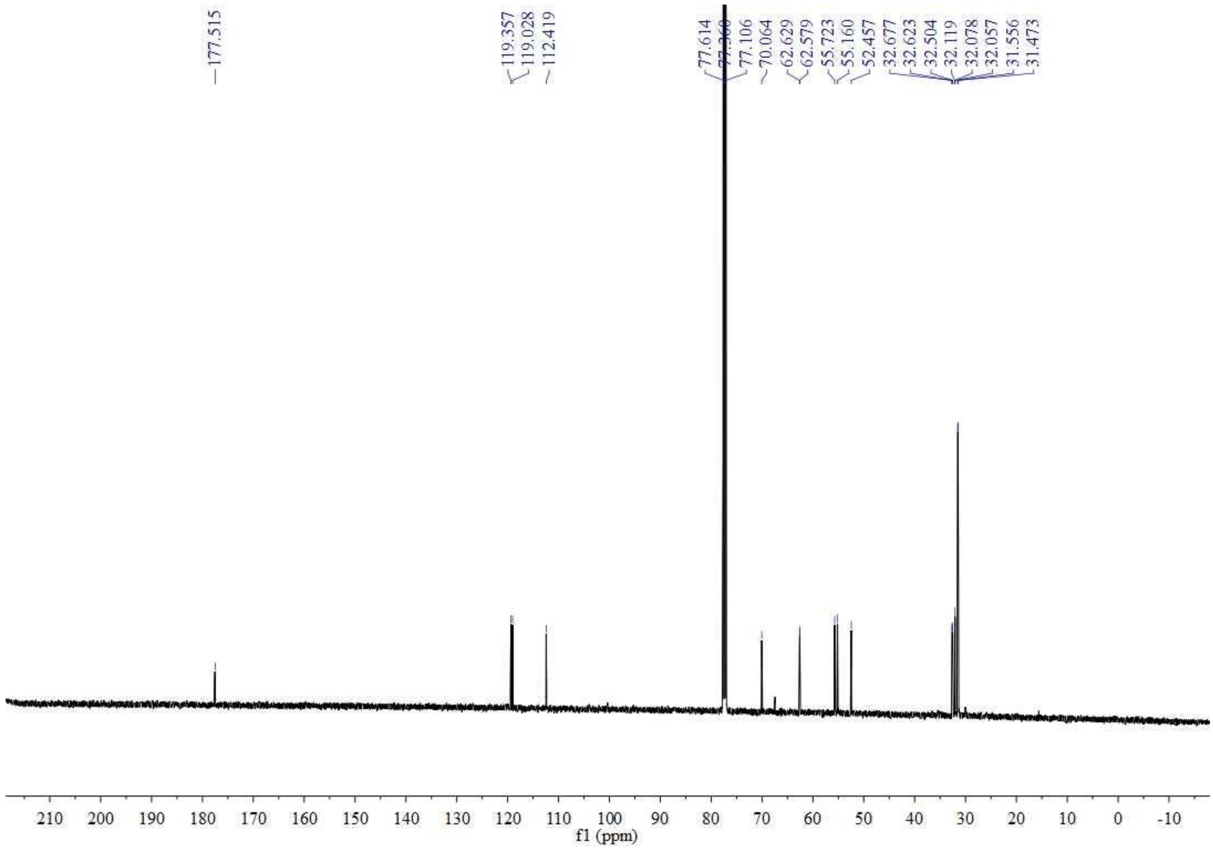

^{13}C NMR (126 MHz, $CDCl_3$) Spectrum of Compound **13**

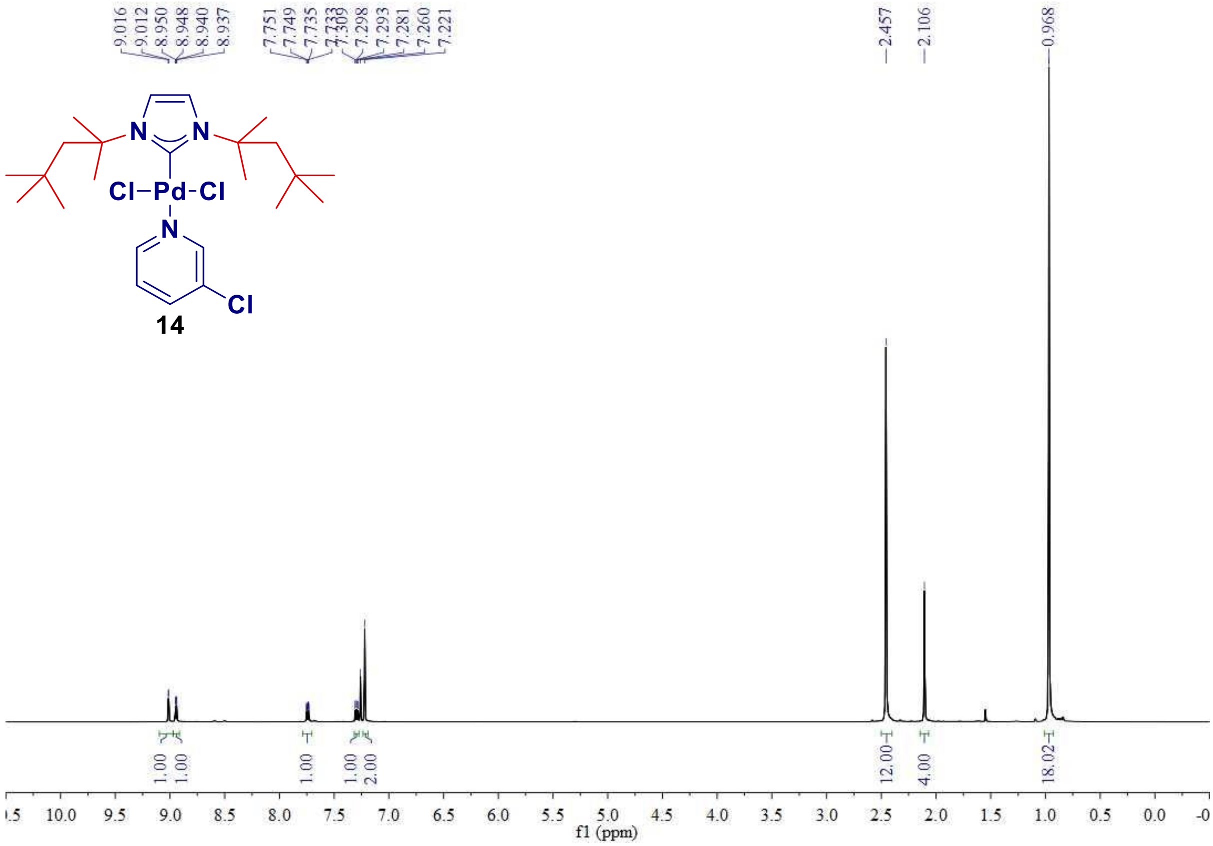

^{1}H NMR (500 MHz, $CDCl_3$) Spectrum of Compound **14**

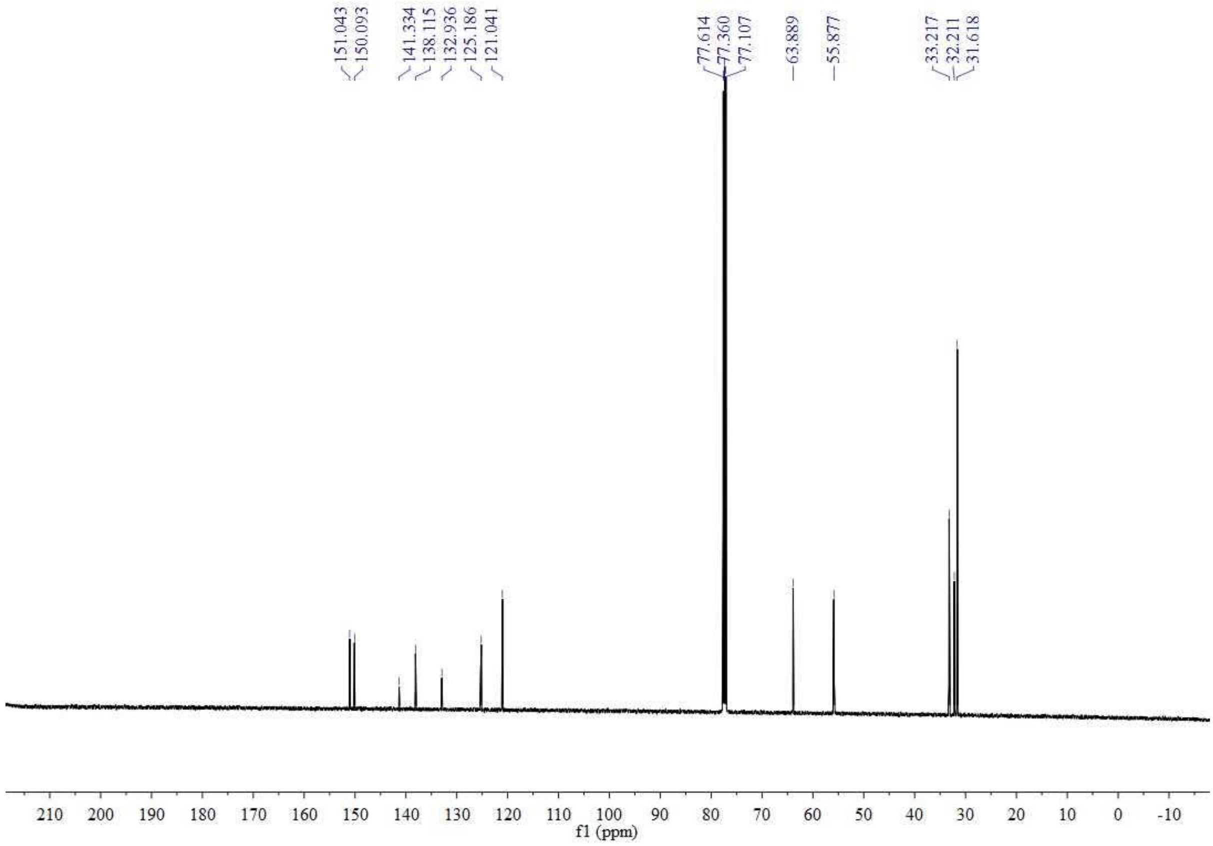

^{13}C NMR (126 MHz, $CDCl_3$) Spectrum of Compound **14**

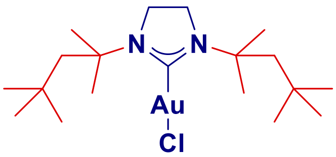

15

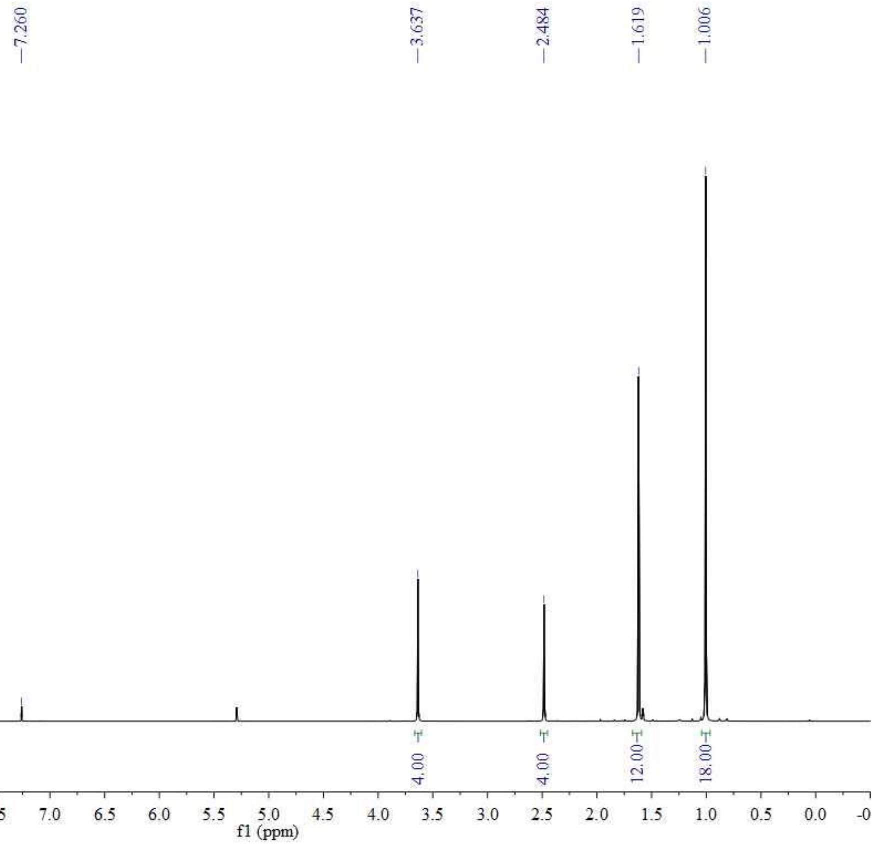

^{1}H NMR (500 MHz, $CDCl_3$) Spectrum of Compound **15**

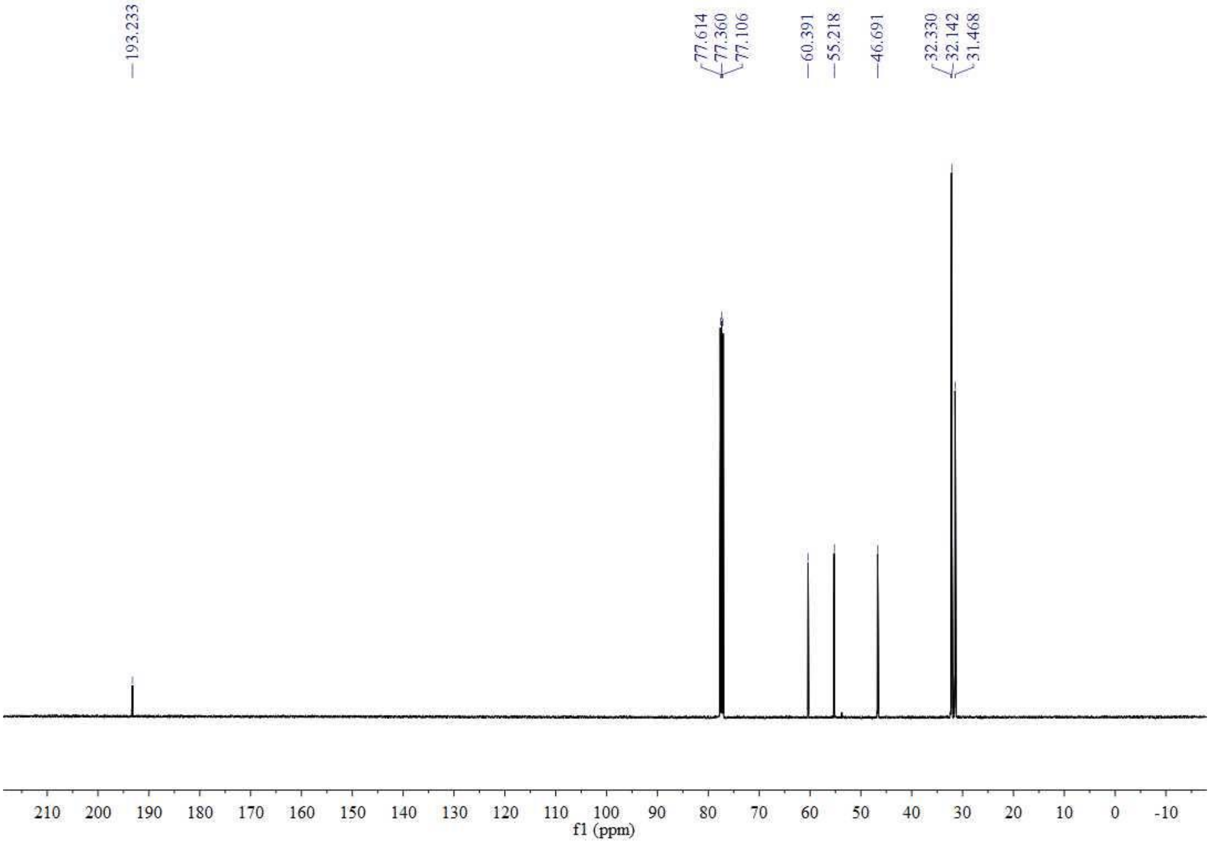

^{13}C NMR (126 MHz, $CDCl_3$) Spectrum of Compound **15**

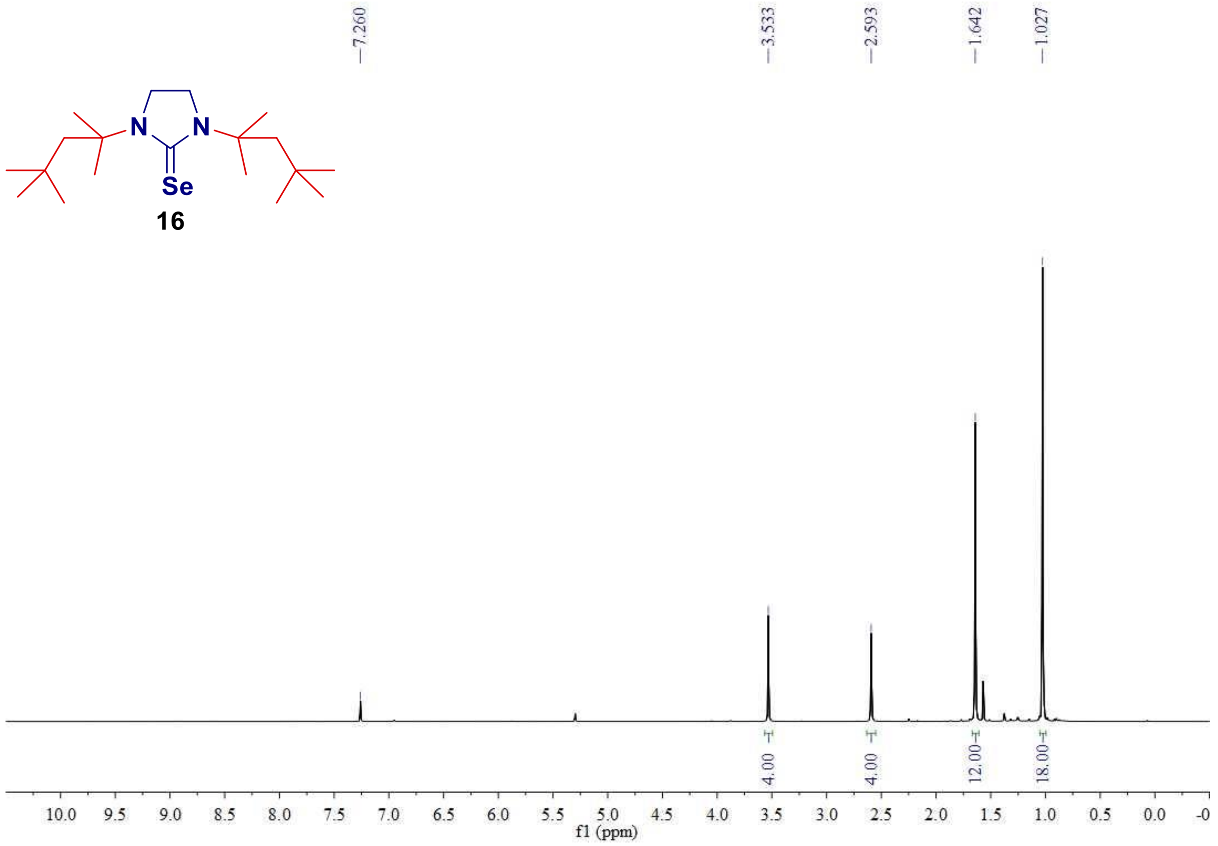

^{1}H NMR (500 MHz, $CDCl_3$) Spectrum of Compound **16**

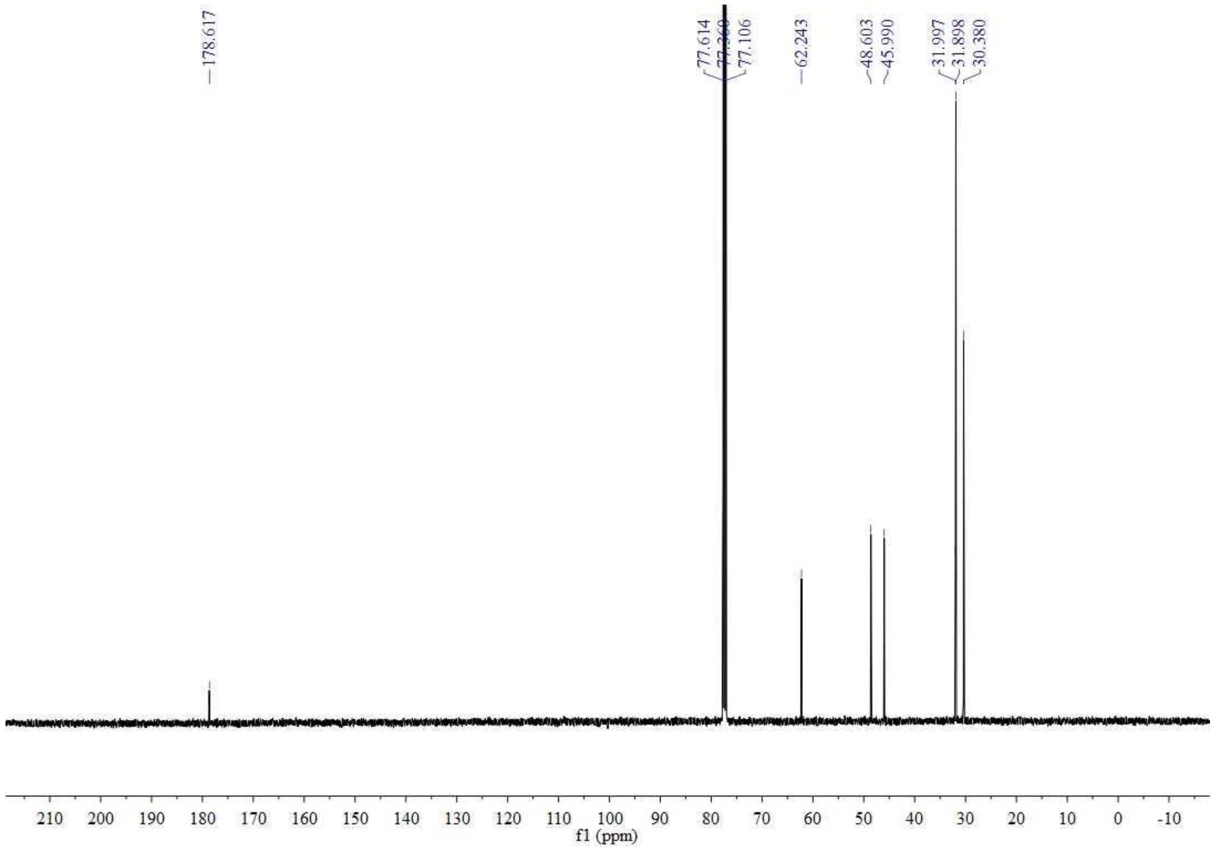

^{13}C NMR (126 MHz, $CDCl_3$) Spectrum of Compound **16**

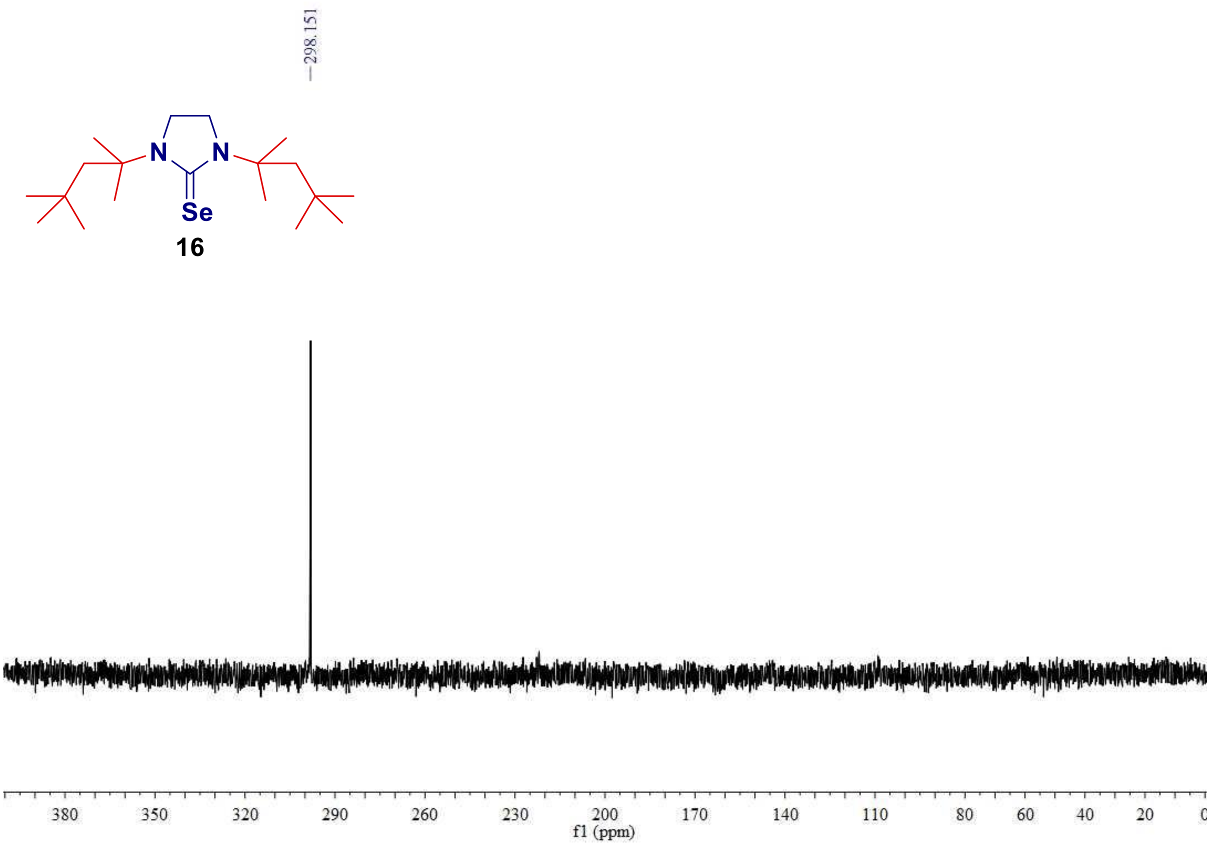

^{77}Se NMR (95 MHz, $CDCl_3$) Spectrum of Compound **16**

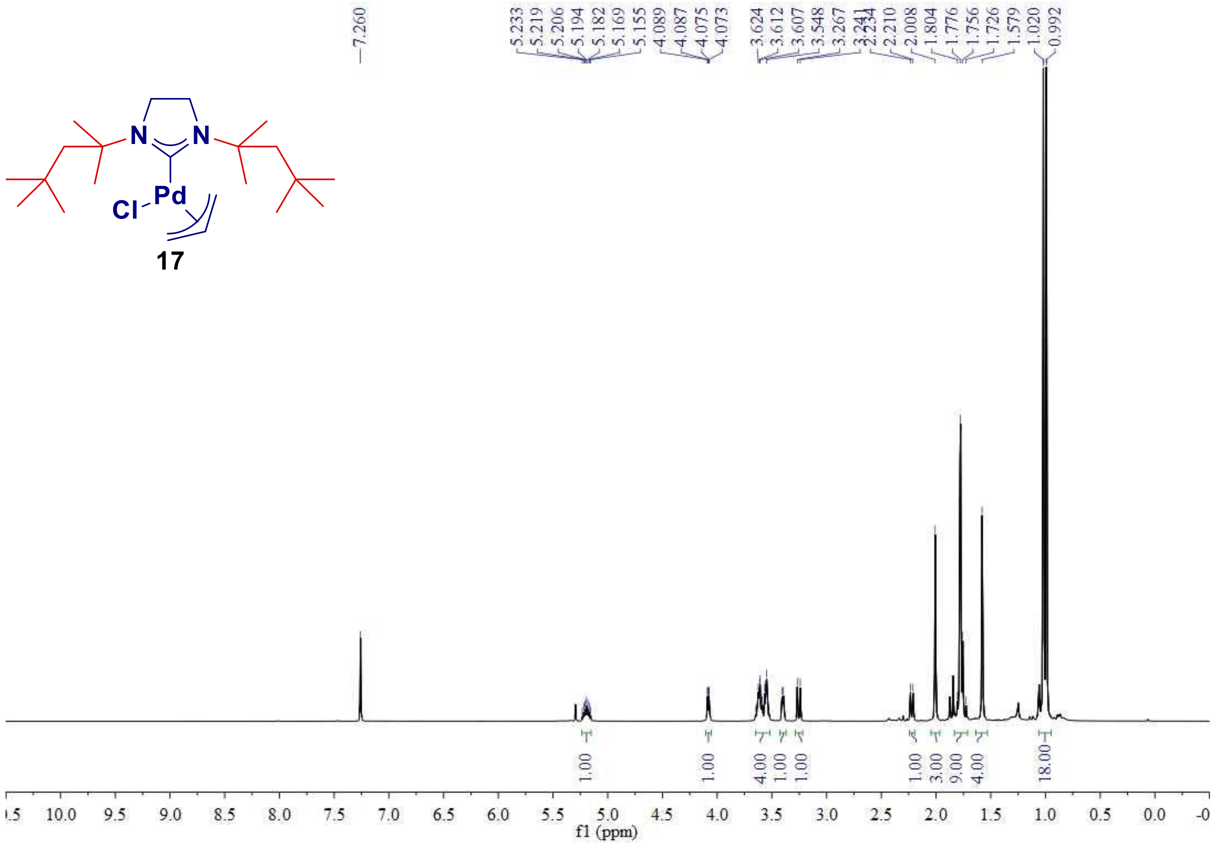

^{1}H NMR (500 MHz, $CDCl_3$) Spectrum of Compound **17**

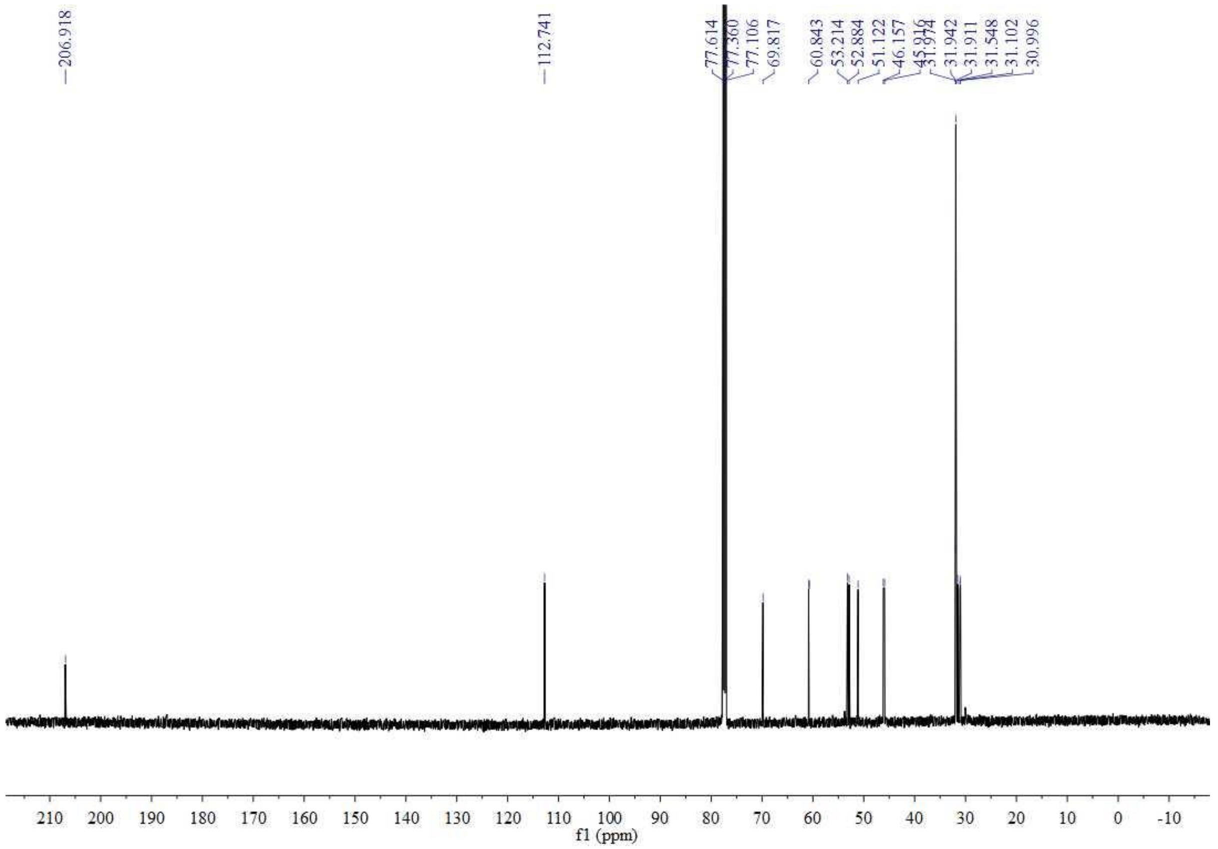

^{13}C NMR (126 MHz, $CDCl_3$) Spectrum of Compound **17**

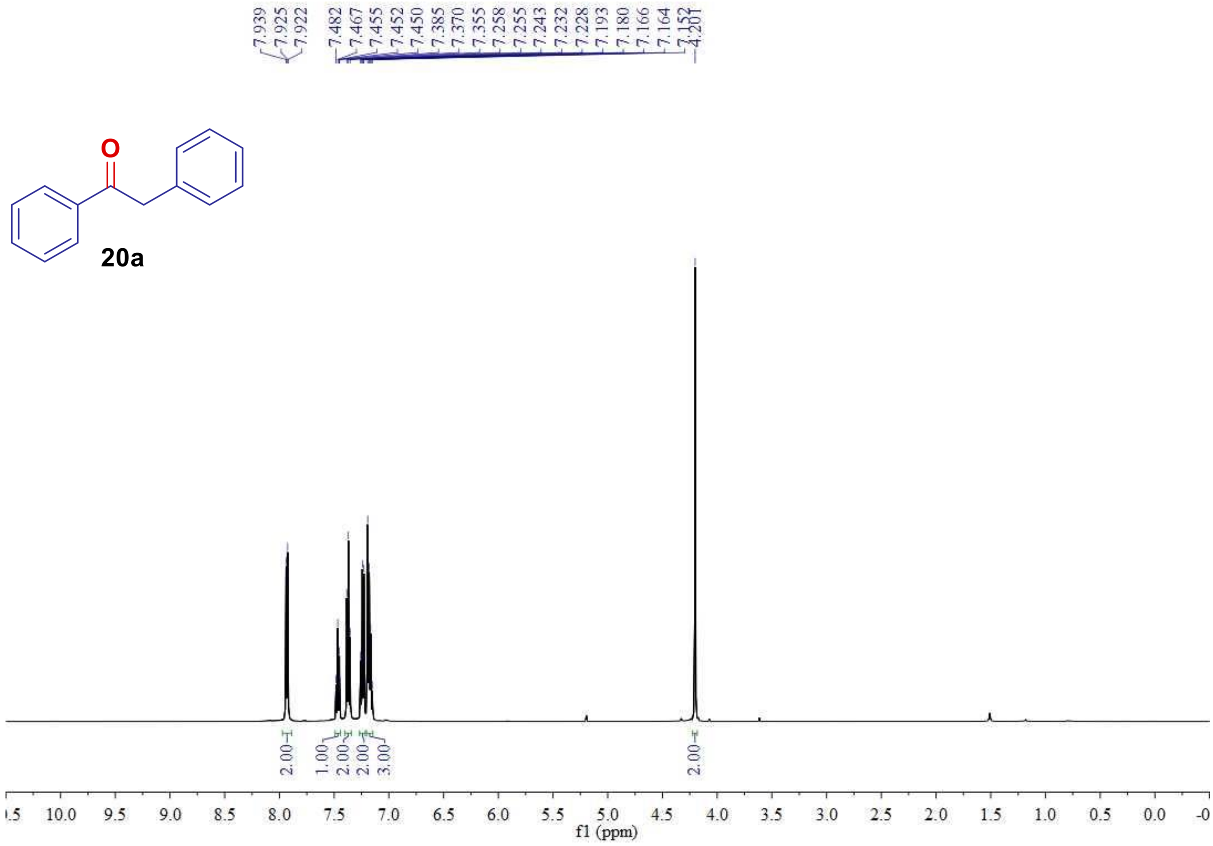

20a

^{1}H NMR (500 MHz, $CDCl_3$) Spectrum of Compound **20a**

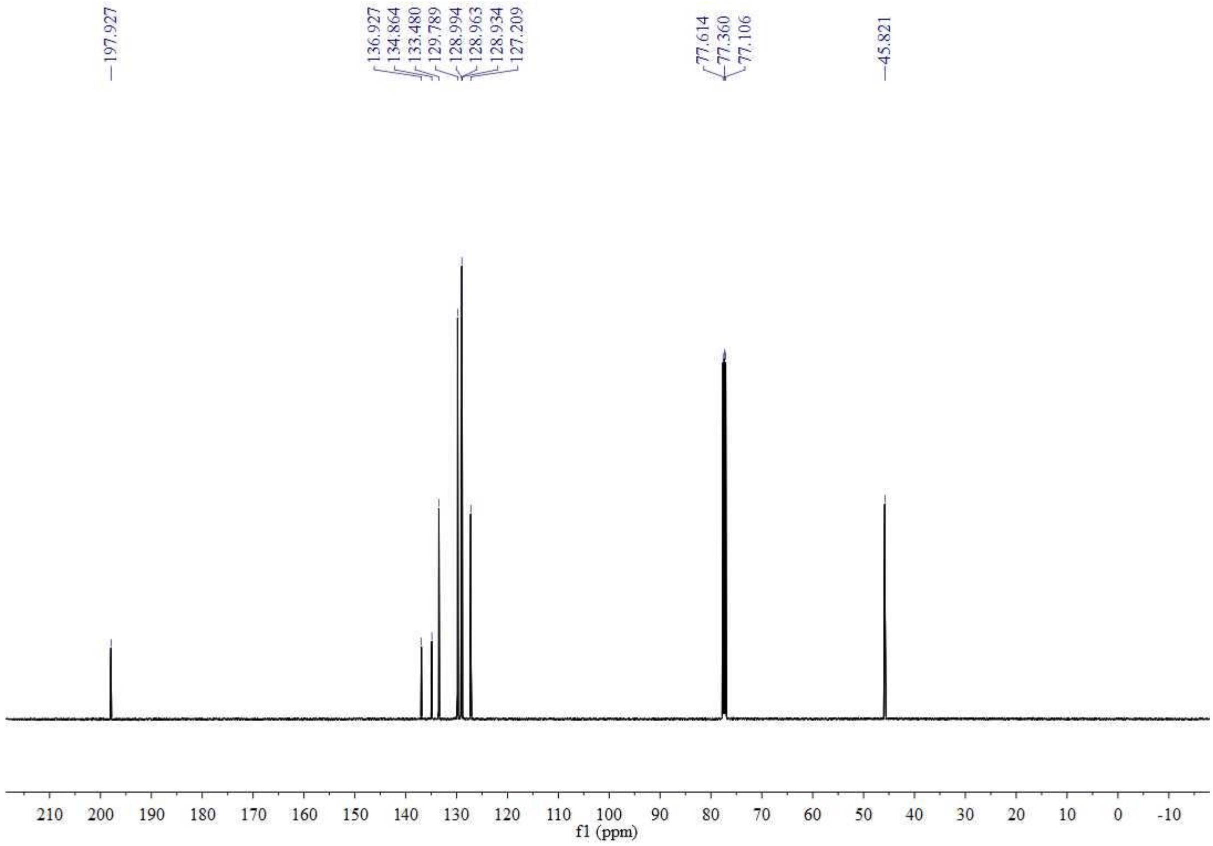

^{13}C NMR (126 MHz, $CDCl_3$) Spectrum of Compound **20a**

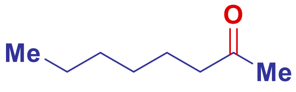

20b

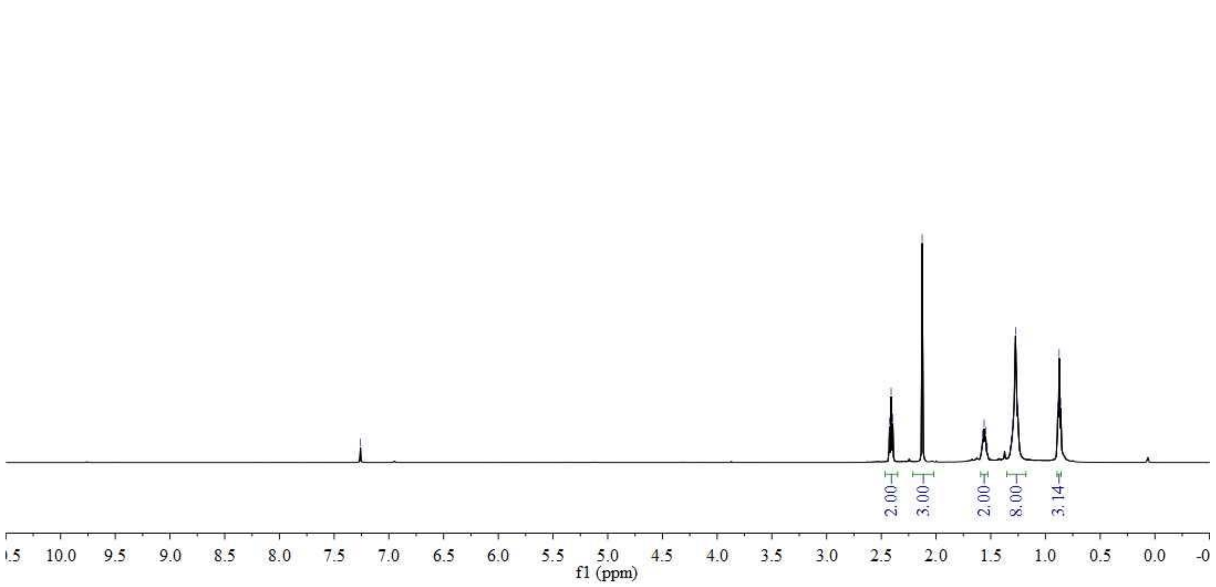

^{1}H NMR (500 MHz, $CDCl_3$) Spectrum of Compound **20b**

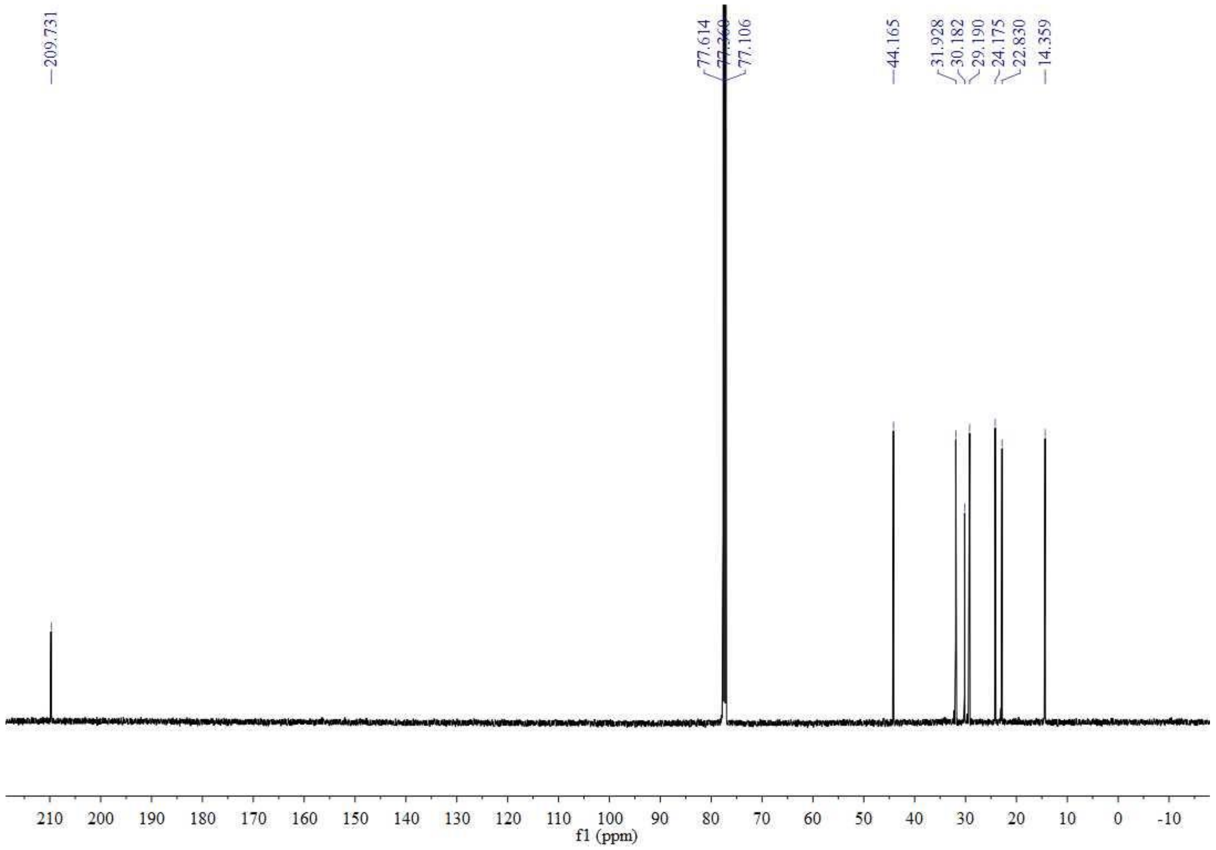

^{13}C NMR (126 MHz, $CDCl_3$) Spectrum of Compound **20b**

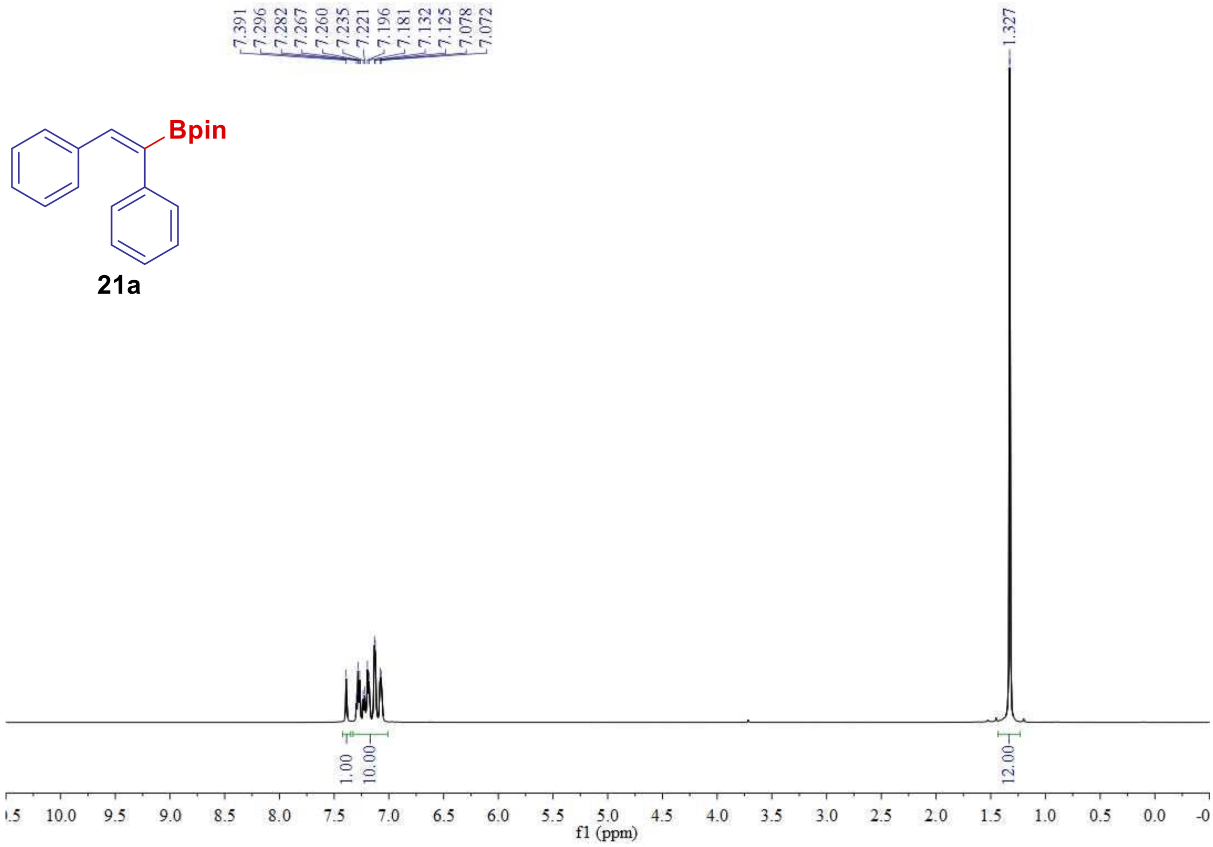

^{1}H NMR (500 MHz, $CDCl_3$) Spectrum of Compound **21a**

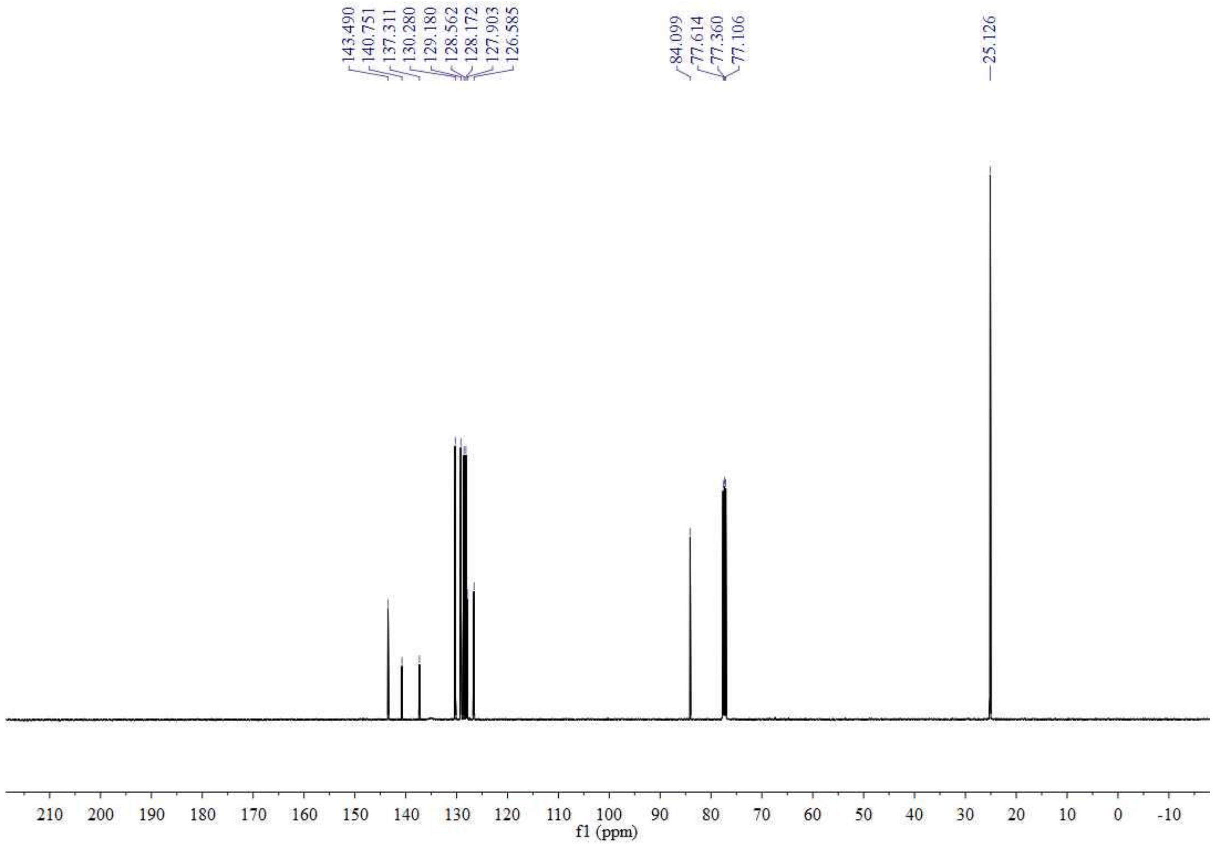

^{13}C NMR (126 MHz, $CDCl_3$) Spectrum of Compound **21a**

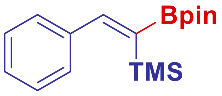

21b

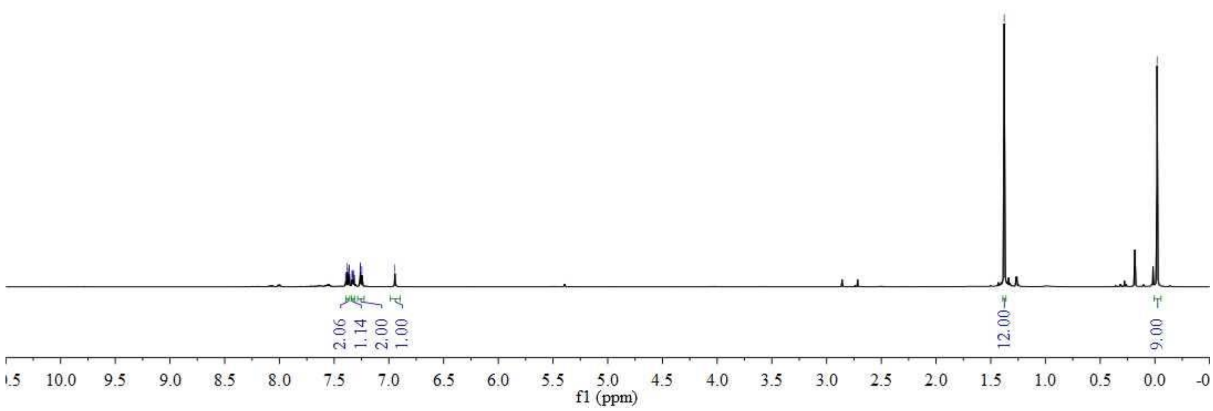

^{1}H NMR (500 MHz, $CDCl_3$) Spectrum of Compound **21b**

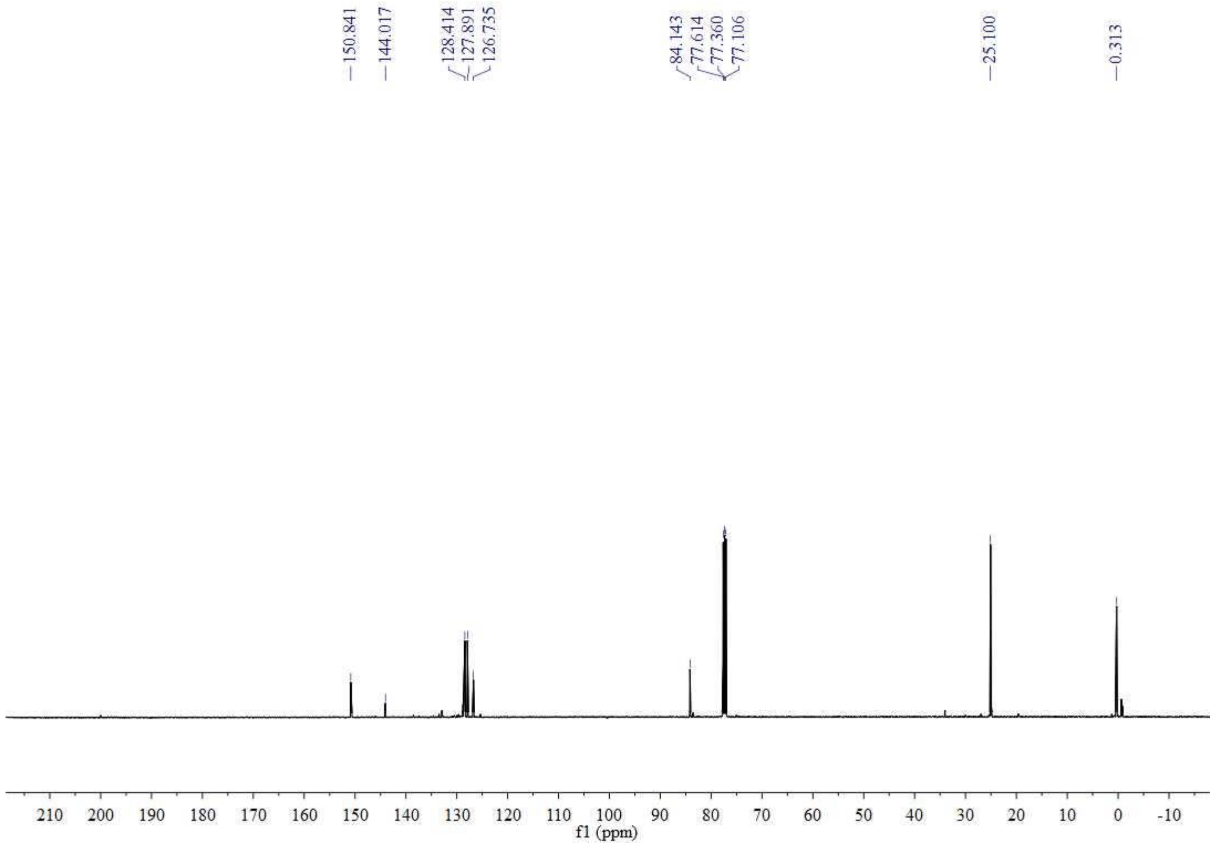

^{13}C NMR (126 MHz, $CDCl_3$) Spectrum of Compound **21b**

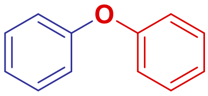

24

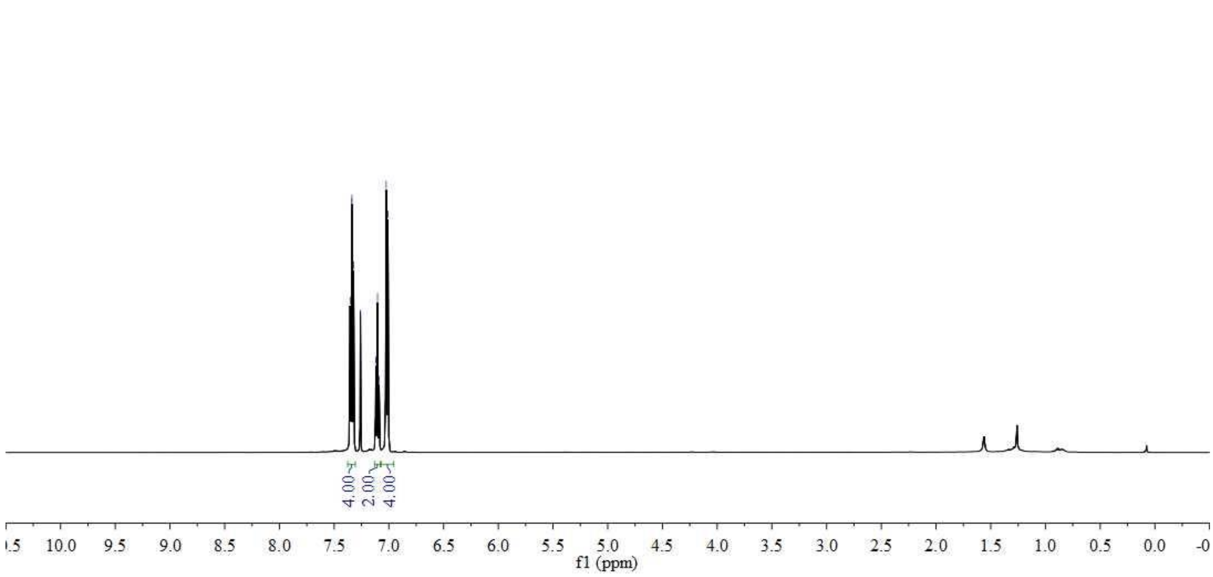

^{1}H NMR (500 MHz, $CDCl_3$) Spectrum of Compound **24**

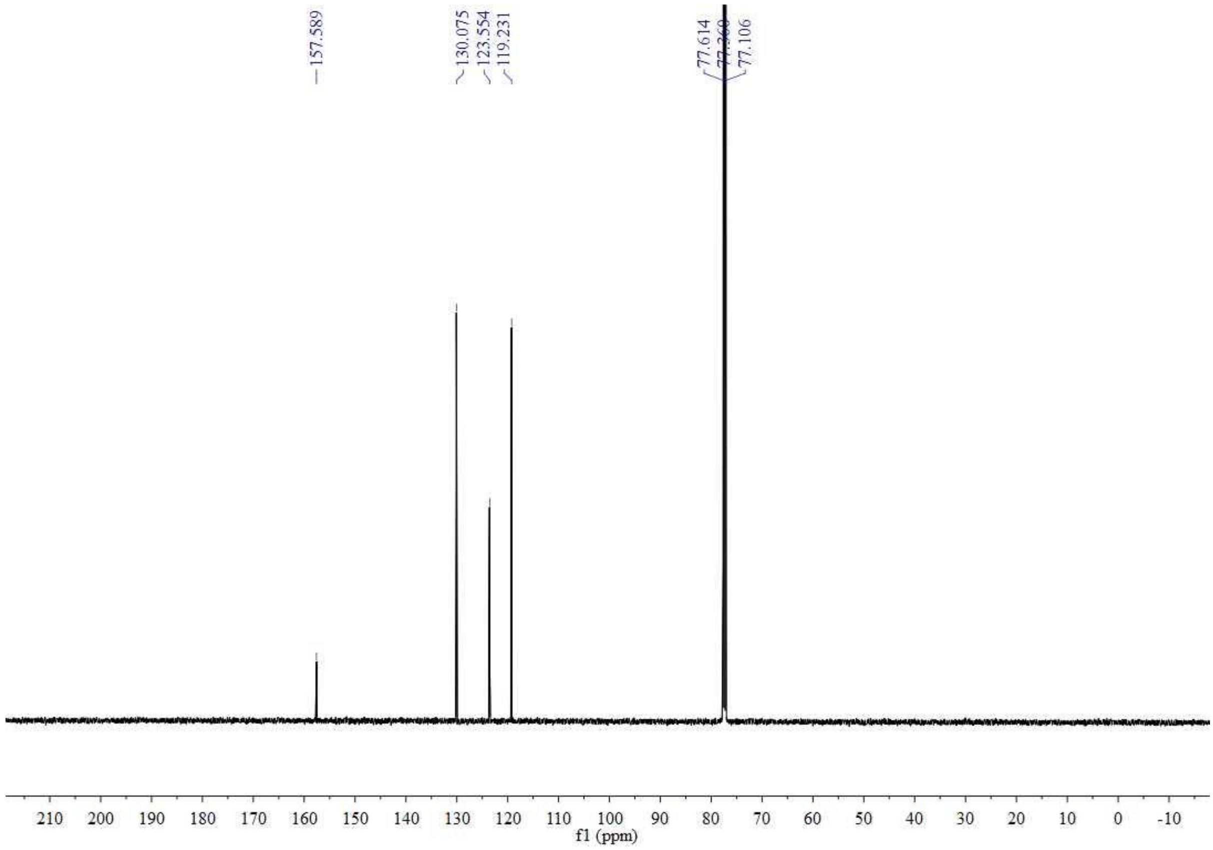

^{13}C NMR (126 MHz, $CDCl_3$) Spectrum of Compound **24**

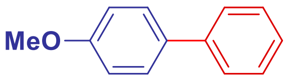

27a

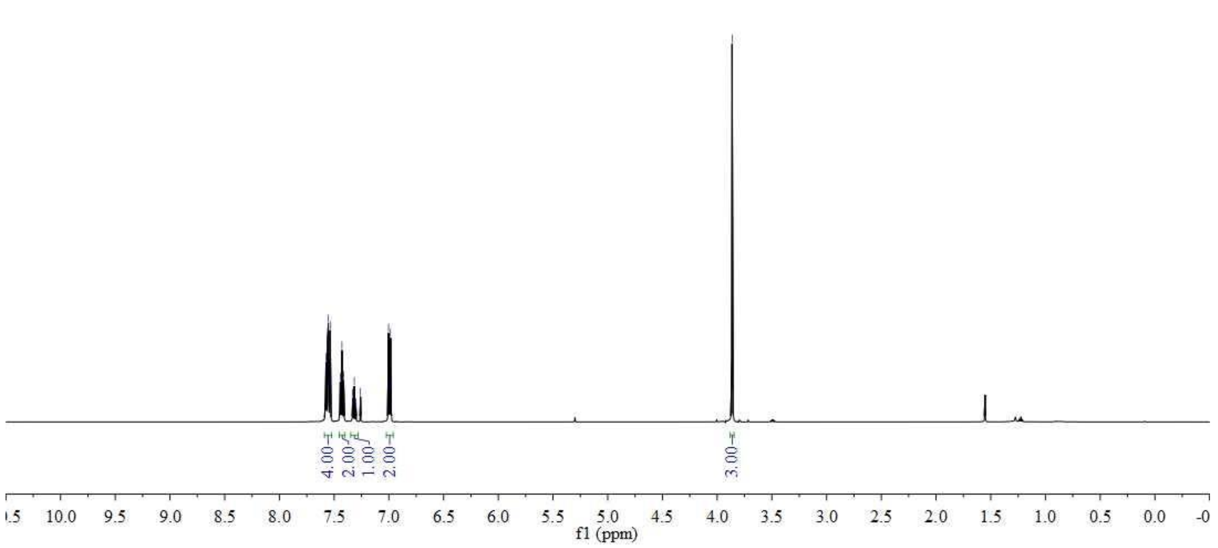

^{1}H NMR (500 MHz, $CDCl_3$) Spectrum of Compound **27a**

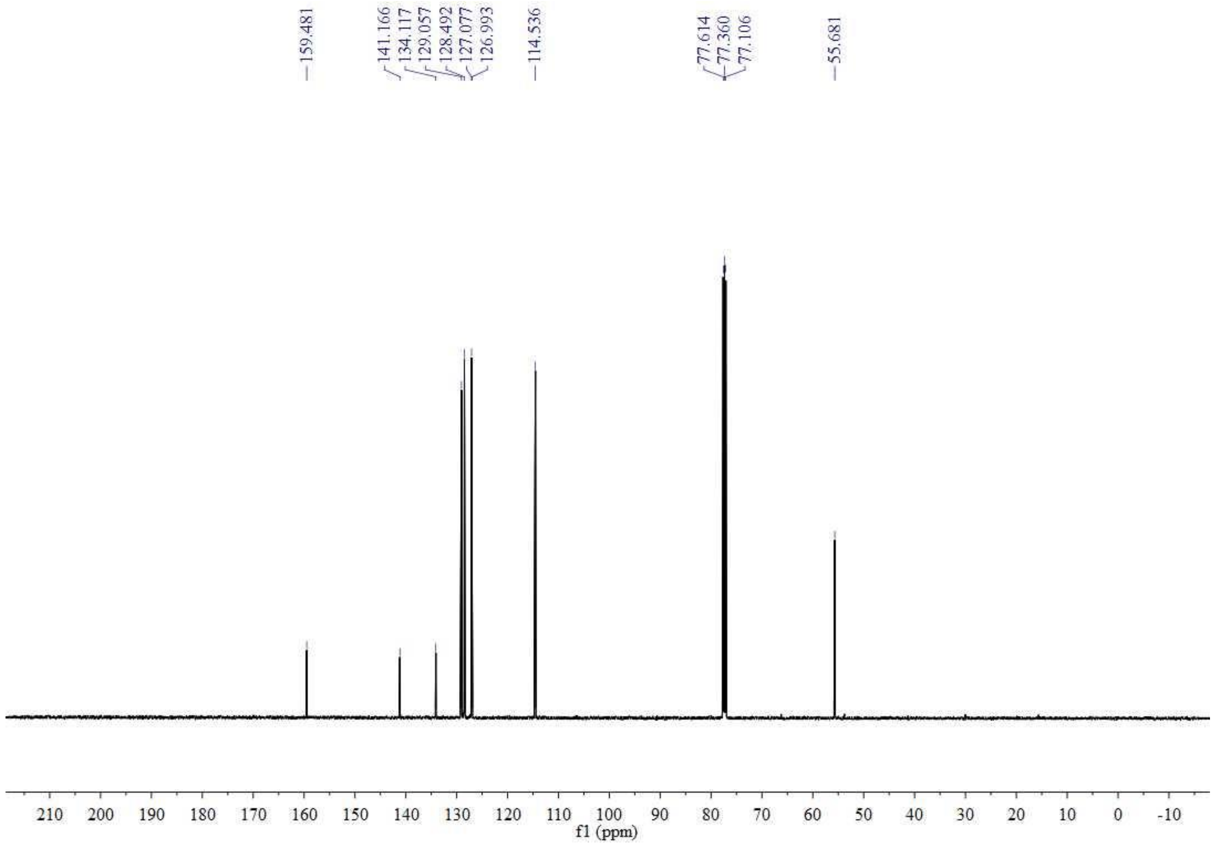

^{13}C NMR (126 MHz, $CDCl_3$) Spectrum of Compound **27a**

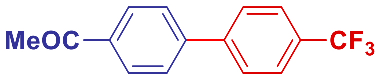

27b

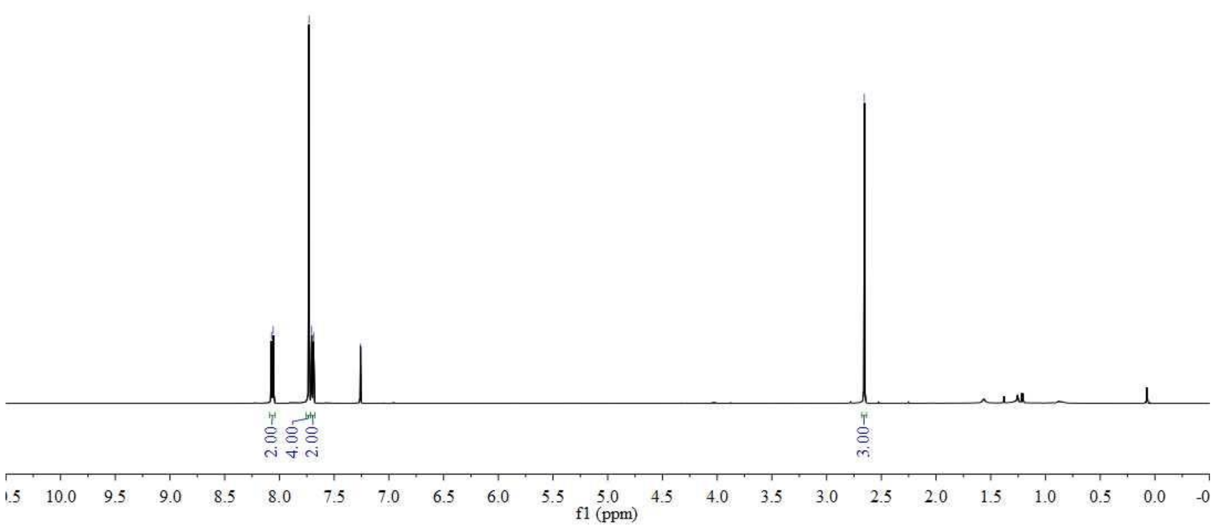

^{1}H NMR (500 MHz, $CDCl_3$) Spectrum of Compound **27b**

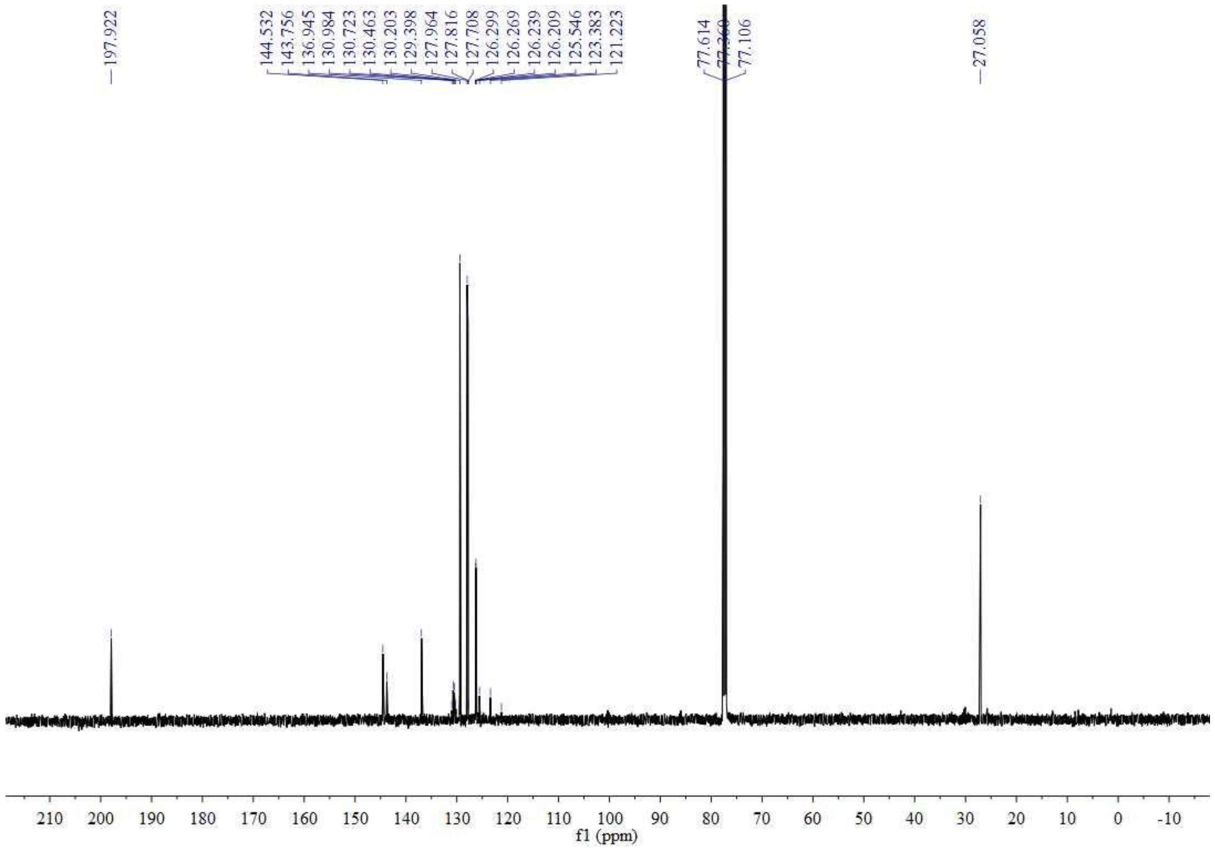

^{13}C NMR (126 MHz, $CDCl_3$) Spectrum of Compound **27b**

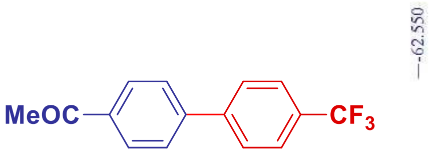

27b

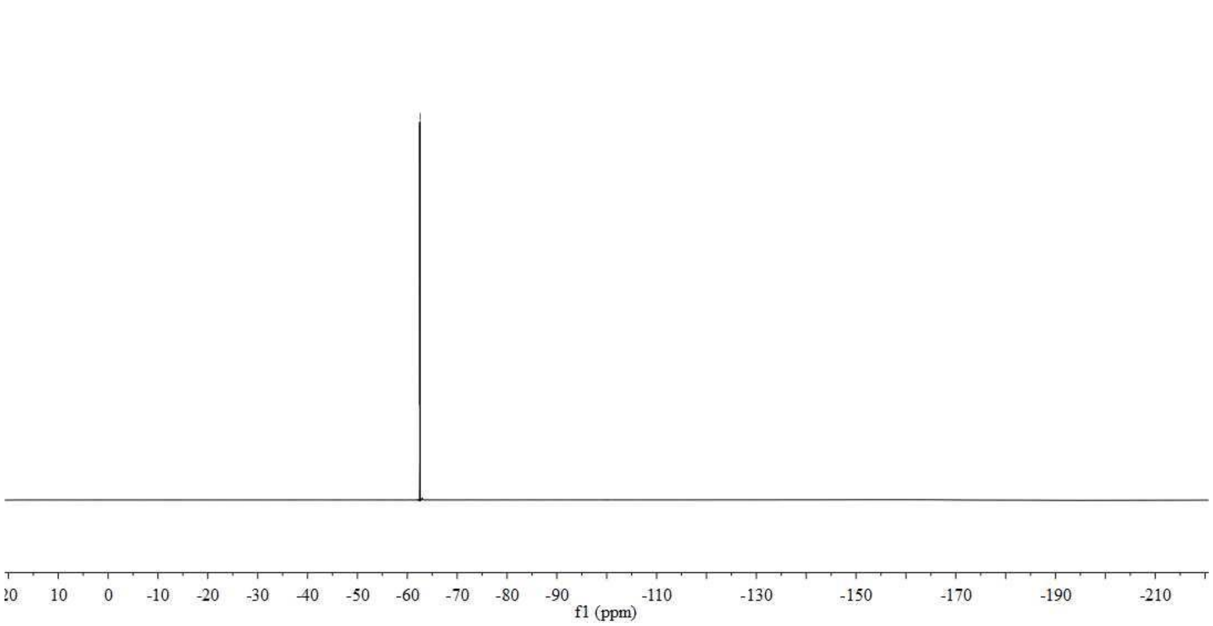

^{19}F NMR (471 MHz, $CDCl_3$) Spectrum of Compound **27b**

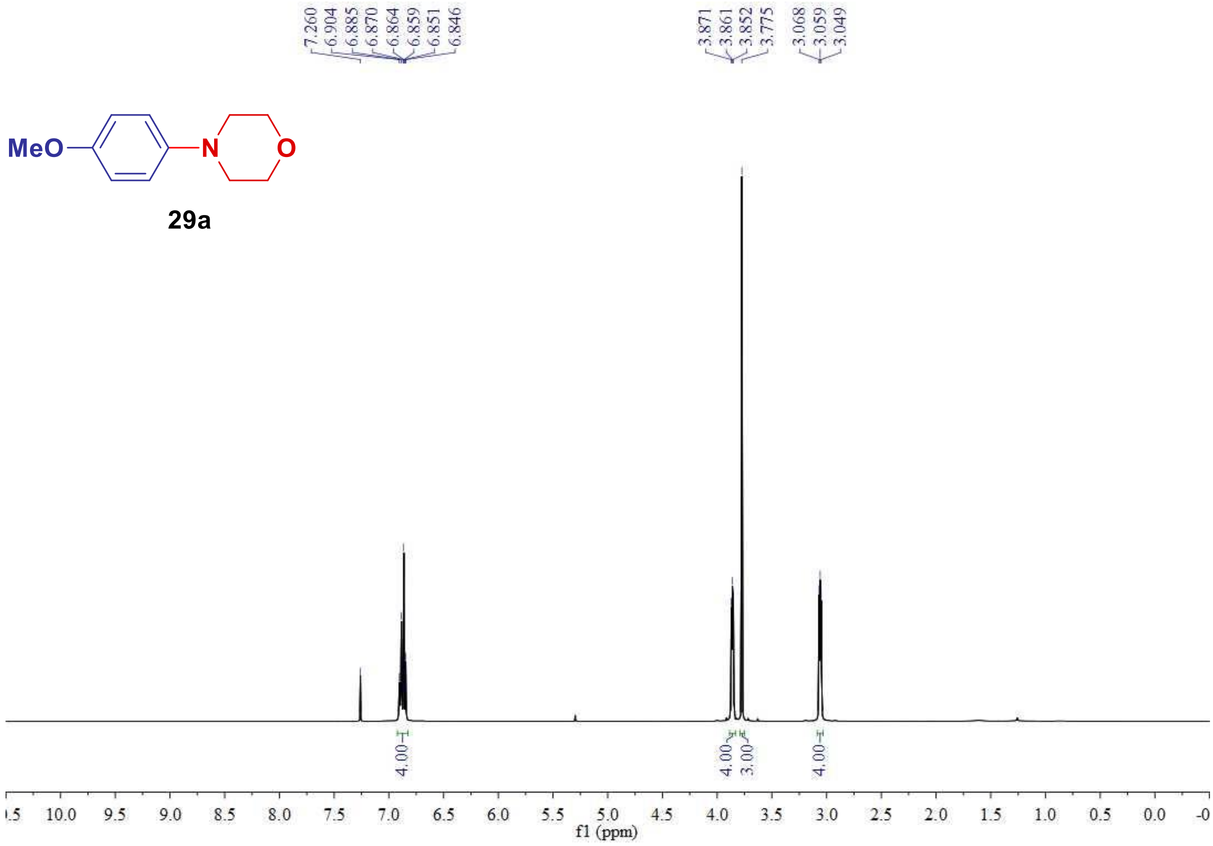

^{1}H NMR (500 MHz, $CDCl_3$) Spectrum of Compound **30a**

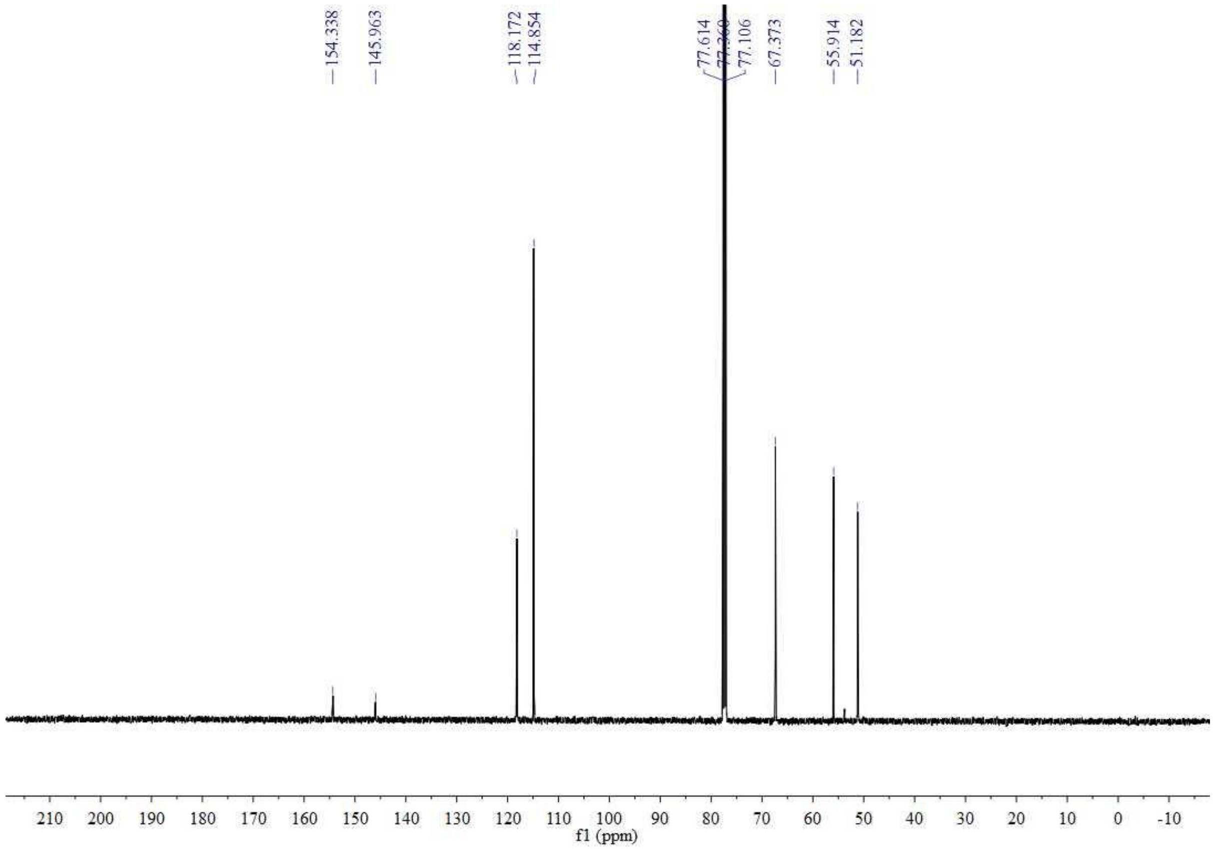

^{13}C NMR (126 MHz, $CDCl_3$) Spectrum of Compound **30a**

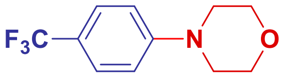

29b

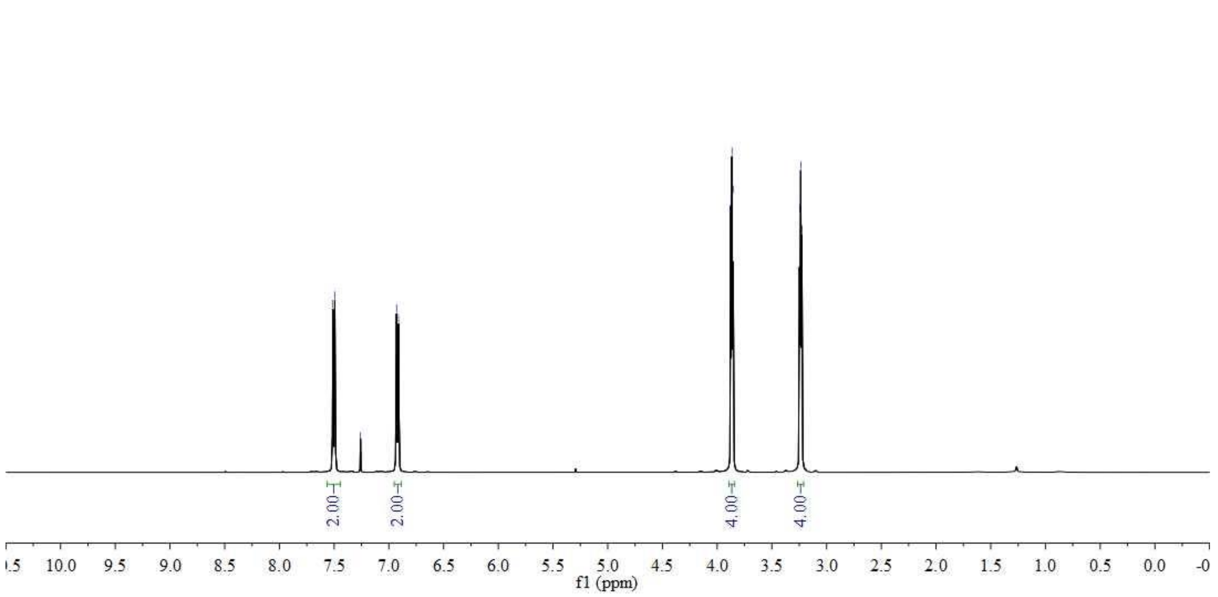

^{1}H NMR (500 MHz, CDCl$_3$) Spectrum of Compound **30b**

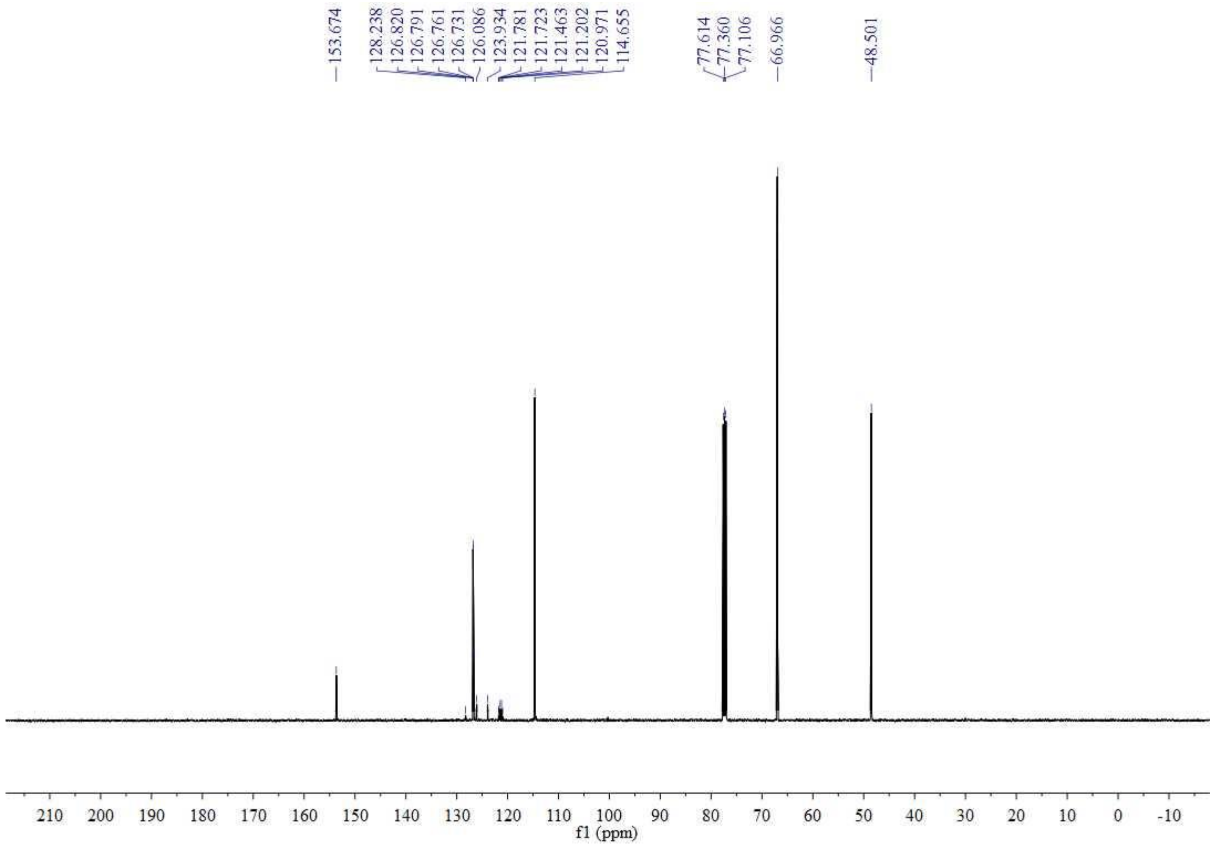

^{13}C NMR (126 MHz, CDCl$_3$) Spectrum of Compound **30b**

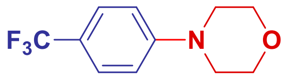

29b

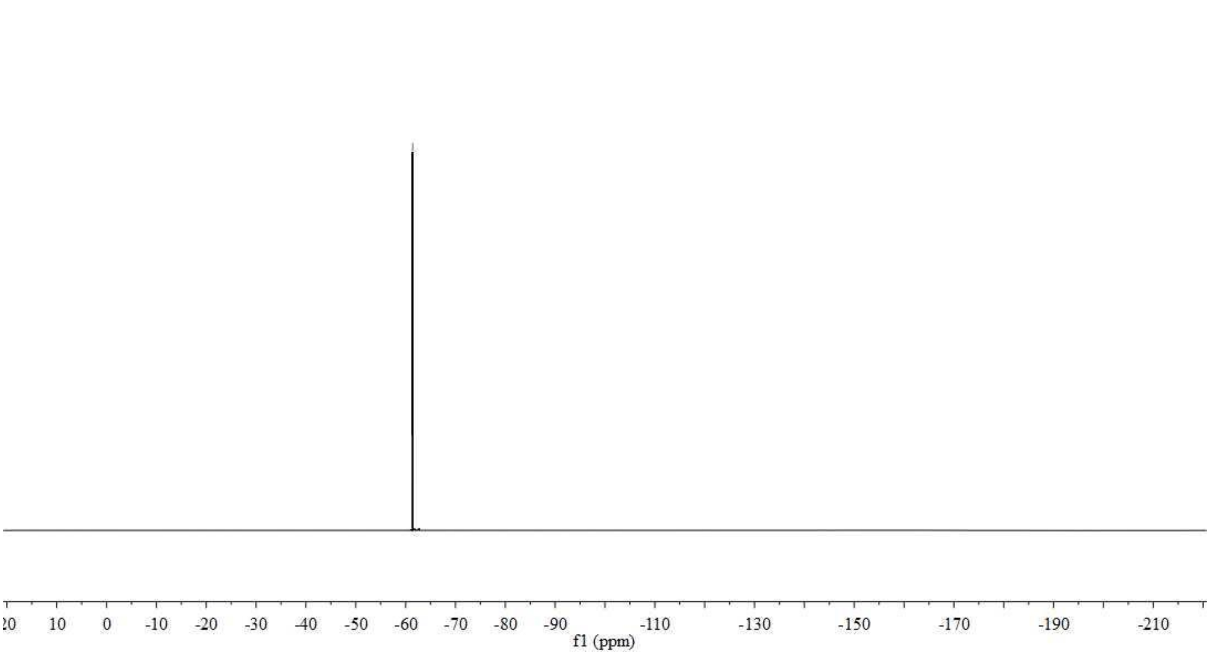

^{19}F NMR (471 MHz, $CDCl_3$) Spectrum of Compound **30b**

ItOct

Energy: -855.402604 au

Sum of electronic and thermal Energies: -854.858757 au

Geometry:

N	0.90871700	0.56719600	0.66538400
N	-0.90871400	-0.56718500	0.66538800
C	0.00000200	0.00000200	-0.18065400
C	-2.84044500	-0.57857600	-0.92574600
H	-3.46254400	-1.30918300	-1.45650200
H	-2.07300800	-0.25595300	-1.63357500
C	1.47839300	2.63039400	-0.51830700
H	0.86123600	2.34381100	-1.37099600
H	2.27989800	3.28873900	-0.86441000
H	0.85424500	3.18694000	0.18605300
C	-4.02309600	1.32249100	-1.98493500
H	-4.69704500	2.17702300	-1.86869000
H	-4.48998000	0.62181000	-2.68491700
H	-3.09569000	1.68260800	-2.43934200
C	2.84044700	0.57857000	-0.92575100
H	3.46255000	1.30917000	-1.45651100
H	2.07300800	0.25594600	-1.63357800
C	2.06545400	1.37530200	0.16165600
C	-1.47838300	-2.63039200	-0.51829000
H	-0.86122800	-2.34381300	-1.37098000
H	-2.27988600	-3.28874200	-0.86438700
H	-0.85423200	-3.18693100	0.18607400
C	2.93922500	1.82864500	1.33837000
H	2.39220100	2.51631600	1.98915000
H	3.80916900	2.36320100	0.95297800

H	3.29898800	0.99624400	1.94368900
C	-0.57175200	-0.36337700	1.99994200
H	-1.14382500	-0.72906200	2.83336900
C	-2.06544700	-1.37529800	0.16166600
C	0.57175100	0.36340500	1.99993900
H	1.14382200	0.72910000	2.83336400
C	-3.74918100	0.65133600	-0.61998100
C	-2.93921500	-1.82863600	1.33838400
H	-2.39218800	-2.51630200	1.98916700
H	-3.80915800	-2.36319600	0.95299600
H	-3.29897900	-0.99623200	1.94369900
C	3.07978000	-1.69010100	0.29901100
H	3.72249200	-2.57182200	0.39333200
H	2.11716300	-2.01265600	-0.10381500
H	2.90402400	-1.30264600	1.30492700
C	3.74917500	-0.65134600	-0.61997700
C	5.11360800	-0.24136100	-0.02602400
H	5.03111000	0.16716700	0.98194000
H	5.60737100	0.50816100	-0.65334500
H	5.77567800	-1.11144600	0.03034400
C	-5.11361000	0.24134700	-0.02602100
H	-5.03110700	-0.16717000	0.98194700
H	-5.60736900	-0.50818300	-0.65333400
H	-5.77568600	1.11142800	0.03034000
C	-3.07979100	1.69010300	0.29899800
H	-2.11717600	2.01265900	-0.10383100
H	-2.90403300	1.30265800	1.30491700
H	-3.72250700	2.57182200	0.39331100
C	4.02308300	-1.32251400	-1.98492700

H	4.69702800	-2.17704900	-1.86867600
H	4.48997000	-0.62184100	-2.68491600
H	3.09567500	-1.68262900	-2.43932900

I*t*Oct-CuCl

Energy: -2956.262915 au

Sum of electronic and thermal Energies: -2955.711971 au

Geometry:

N	0.91706600	-1.18302200	0.57844600
N	-0.91706700	-1.18301300	-0.57845800
C	-0.00000100	-0.35284500	0.00000100
C	-3.00366900	0.25155200	-0.57571800
H	-3.68070700	0.70586200	-1.30791200
H	-2.34952700	1.06735200	-0.25080400
C	1.56199500	0.04261700	2.61401600
H	1.05727000	0.96427700	2.31803700
H	2.38510400	0.30624000	3.28219400
H	0.85309500	-0.57784100	3.16824300
C	-4.32457300	1.16205100	1.30623100
H	-4.97483000	0.96491200	2.16378000
H	-4.88310900	1.78348100	0.59935300
H	-3.46847200	1.74528200	1.65572700
C	3.00366900	0.25154500	0.57572700
H	3.68070400	0.70584500	1.30793000
H	2.34952700	1.06734900	0.25082400
C	2.10596900	-0.72334600	1.38962400
C	-1.56199700	0.04265500	-2.61401000
H	-1.05727200	0.96431200	-2.31801800
H	-2.38510500	0.30629000	-3.28218400

H	-0.85309700	-0.57779400	-3.16824700
C	2.86953100	-1.95072200	1.90574600
H	2.25940800	-2.52917400	2.60453700
H	3.75323600	-1.60840200	2.44588500
H	3.20665200	-2.61158700	1.10782700
C	-0.56994000	-2.50713300	-0.36448900
H	-1.13940900	-3.33977700	-0.73083500
C	-2.10597000	-0.72332600	-1.38963000
C	0.56994100	-2.50713900	0.36445400
H	1.13940800	-3.33978800	0.73079000
C	-3.87316900	-0.16339200	0.65105100
C	-2.86953200	-1.95069300	-1.90577200
H	-2.25941100	-2.52913100	-2.60457500
H	-3.75323800	-1.60836400	-2.44590400
H	-3.20665200	-2.61157400	-1.10786500
C	3.09698100	-0.97948600	-1.70101000
H	3.72697800	-1.14625300	-2.58042200
H	2.19831200	-0.45220500	-2.03071600
H	2.79393600	-1.95996500	-1.32631500
C	3.87317100	-0.16338100	-0.65104600
C	5.14704600	-0.93292700	-0.24139400
H	4.93791300	-1.93408900	0.13818000
H	5.70604800	-0.39037300	0.52768300
H	5.80690600	-1.04815200	-1.10682400
C	-5.14704300	-0.93293500	0.24139100
H	-4.93790700	-1.93409100	-0.13819900
H	-5.70604800	-0.39037200	-0.52767700
H	-5.80690000	-1.04817600	1.10682000
C	-3.09697600	-0.97950900	1.70100400

H	-2.19831000	-0.45222900	2.03072000
H	-2.79392600	-1.95998100	1.32629500
H	-3.72697400	-1.14629200	2.58041200
C	4.32457300	1.16207200	-1.30620800
H	4.97483100	0.96494600	-2.16376000
H	4.88310800	1.78349200	-0.59932200
H	3.46847100	1.74530700	-1.65569700
Cl	-0.00000300	3.70252000	0.00000700
Cu	-0.00000100	1.56779700	0.00000700

SI*t*Oct

Energy: -856.603327 au

Sum of electronic and thermal Energies: -856.036257 au

Geometry:

N	-0.99689800	0.58505100	-0.81803600
N	0.99646200	-0.27383700	-0.96735200
C	-0.00026500	0.01924600	-0.10884700
C	2.68720100	-0.79365500	0.79930300
H	3.27536800	-1.65418800	1.14043700
H	1.81084200	-0.74179600	1.44870400
C	-1.58276500	2.63307200	0.41698400
H	-0.86211700	2.42426900	1.21024200
H	-2.39192900	3.24267700	0.82954000
H	-1.07971000	3.22147000	-0.35617800
C	3.57310500	0.64880400	2.60893100
H	4.20182300	1.49753600	2.89627300
H	3.97131200	-0.24383000	3.10252900
H	2.56631800	0.82616600	2.99769400
C	-2.68771000	0.47782300	1.02050200

H	-3.27680300	1.17254000	1.63137400
H	-1.81143600	0.21101100	1.61495000
C	-2.12821700	1.30963600	-0.16763400
C	1.58122800	-2.61879200	-0.49545300
H	0.86005200	-2.68920400	0.32132800
H	2.38998700	-3.33209600	-0.31196100
H	1.07871700	-2.91208500	-1.42201400
C	-3.19380100	1.65199000	-1.22164900
H	-2.81460500	2.37704000	-1.94673400
H	-4.05822200	2.10427500	-0.73210200
H	-3.53678800	0.77296600	-1.76850600
C	0.60864700	-0.05337100	-2.38346400
H	1.40631100	0.41205500	-2.96252200
C	2.12731400	-1.17595200	-0.59953000
C	-0.60892700	0.85559000	-2.22538600
H	-1.40654000	0.61316300	-2.92765100
C	3.55264000	0.47605400	1.07313300
C	3.19279800	-1.14351600	-1.70730000
H	2.81328300	-1.58141400	-2.63436700
H	4.05666500	-1.73506800	-1.39875900
H	3.53663000	-0.13191400	-1.92569600
C	-2.97056600	-1.81229700	-0.16519300
H	-3.57559600	-2.72552700	-0.17380800
H	-1.94458600	-2.08322300	0.09110400
H	-2.96146100	-1.41079900	-1.18103600
C	-3.55183500	-0.81070200	0.84995500
C	-5.01495700	-0.49159400	0.47429200
H	-5.11420600	-0.08659100	-0.53346700
H	-5.45217000	0.23071800	1.17163300

H	-5.62178900	-1.40183900	0.51936800
C	5.01544300	0.30098000	0.61105100
H	5.11436900	0.25921900	-0.47426200
H	5.45186300	-0.61457500	1.02392400
H	5.62320600	1.14214200	0.96018200
C	2.97220700	1.76202900	0.45572200
H	1.94620400	1.93103400	0.78805700
H	2.96363300	1.72763700	-0.63608000
H	3.57756100	2.62414800	0.75640100
C	-3.57142700	-1.49172300	2.23735700
H	-4.19891200	-2.38849600	2.22138900
H	-3.97051500	-0.81860200	3.00328700
H	-2.56424700	-1.78856100	2.54346200
H	-0.34176700	1.91191500	-2.35931300
H	0.34161700	-1.00240300	-2.86633900

SI*t*Oct-CuCl

Energy: -2957.460555 au

Sum of electronic and thermal Energies: -2956.886249 au

Geometry:

N	0.97764400	-1.23840200	0.50634100
N	-0.97764100	-1.23840000	-0.50633800
C	0.00000300	-0.45767000	0.00000100
C	-2.96404700	0.32712700	-0.60003800
H	-3.60881100	0.78385400	-1.35961400
H	-2.26471200	1.12058900	-0.31522100
C	1.55429200	-0.10081500	2.62804200
H	0.99425800	0.80749500	2.39558500
H	2.36225700	0.16490800	3.31446000

H	0.88503200	-0.79530300	3.14419500
C	-4.19609200	1.41350900	1.25391200
H	-4.85863000	1.30172800	2.11763400
H	-4.70113300	2.05646600	0.52642900
H	-3.29221500	1.93333700	1.58226400
C	2.96404600	0.32713000	0.60003600
H	3.60881000	0.78385700	1.35961200
H	2.26470800	1.12058800	0.31522100
C	2.12643500	-0.74901200	1.34739000
C	-1.55428300	-0.10081000	-2.62804000
H	-0.99426000	0.80750400	-2.39557800
H	-2.36224800	0.16490200	-3.31446200
H	-0.88501400	-0.79529300	-3.14418700
C	2.98484000	-1.95102600	1.77636600
H	2.43409900	-2.61804400	2.44418400
H	3.85205000	-1.58302600	2.32665300
H	3.35232900	-2.53433500	0.93290600
C	-0.57236500	-2.65996400	-0.50294000
H	-1.38605200	-3.31776600	-0.20315400
C	-2.12642900	-0.74901100	-1.34739000
C	0.57236700	-2.65996400	0.50294200
H	1.38605500	-3.31776700	0.20315900
C	-3.86260500	0.03366700	0.64184300
C	-2.98483000	-1.95102500	-1.77637100
H	-2.43407900	-2.61805600	-2.44416800
H	-3.85202500	-1.58302700	-2.32668300
H	-3.35234200	-2.53432100	-0.93291200
C	3.16202500	-0.81376800	-1.72013500
H	3.81140600	-0.90819300	-2.59647300

H	2.22675000	-0.35237400	-2.04561200
H	2.93557300	-1.82413100	-1.36975900
C	3.86260200	0.03367200	-0.64184600
C	5.20059300	-0.63410100	-0.25703900
H	5.08121300	-1.65959500	0.09525400
H	5.71257600	-0.06638300	0.52638600
H	5.86483000	-0.66625300	-1.12635600
C	-5.20059900	-0.63409500	0.25703100
H	-5.08122500	-1.65958800	-0.09526900
H	-5.71257800	-0.06636900	-0.52639100
H	-5.86483600	-0.66624900	1.12634800
C	-3.16203500	-0.81378400	1.72012800
H	-2.22675900	-0.35239900	2.04561300
H	-2.93558700	-1.82414600	1.36974600
H	-3.81142000	-0.90821200	2.59646200
C	4.19609700	1.41351600	-1.25390600
H	4.85862700	1.30173700	-2.11763600
H	4.70115100	2.05646100	-0.52642200
H	3.29222300	1.93335600	-1.58224600
H	0.23195900	-2.96194800	1.50059900
H	-0.23195800	-2.96194700	-1.50059800
Cl	-0.00000600	3.60677900	0.00000400
Cu	-0.00000100	1.46961500	0.00000100

I*t*Bu

Energy: -540.831530 au

Sum of electronic and thermal Energies: -540.523264 au

Geometry:

C	0.00000000	0.00000000	0.55773700

N	0.00000600	1.07007700	-0.28913800
C	-0.00005000	2.47454800	0.22220500
C	1.26205600	2.67951000	1.07613600
C	-1.26241600	2.67954700	1.07574300
C	0.00015300	3.46473500	-0.94941400
C	0.00000000	0.67750700	-1.62503000
H	2.16330300	2.53570700	0.47377800
H	1.28017400	1.96185400	1.89682000
H	1.27890800	3.69293800	1.48710000
H	-2.16348000	2.53574500	0.47311200
H	-1.27938600	3.69298200	1.48668700
H	-1.28079300	1.96190700	1.89643700
H	-0.88876400	3.35428400	-1.57599400
H	0.88922000	3.35418000	-1.57576400
H	0.00015700	4.48191500	-0.55210000
H	-0.00000800	1.35801800	-2.45715200
N	-0.00000600	-1.07007700	-0.28913800
C	0.00005000	-2.47454800	0.22220500
C	-1.26205600	-2.67951000	1.07613600
C	1.26241600	-2.67954700	1.07574300
C	-0.00015300	-3.46473500	-0.94941400
C	0.00000000	-0.67750700	-1.62503000
H	-2.16330300	-2.53570700	0.47377800
H	-1.28017400	-1.96185400	1.89682000
H	-1.27890800	-3.69293800	1.48710000
H	2.16348000	-2.53574500	0.47311200
H	1.27938600	-3.69298200	1.48668700
H	1.28079300	-1.96190700	1.89643700
H	0.88876400	-3.35428400	-1.57599400

H	-0.88922000	-3.35418000	-1.57576400
H	-0.00015700	-4.48191500	-0.55210000
H	0.00000800	-1.35801800	-2.45715200

I*t*Bu-CuCl

Energy: -2641.692228 au

Sum of electronic and thermal Energies: -2641.376857 au

Geometry:

C	0.00000000	0.00000000	-0.45065400
N	0.00000000	1.08302600	-1.28149000
C	-0.00006400	2.51605700	-0.81379200
C	1.26811100	2.77048100	0.01707200
C	-1.26854200	2.77040200	0.01665200
C	0.00015300	3.45051300	-2.03203300
C	0.00001000	0.67681400	-2.60686100
H	2.16556000	2.56553700	-0.57234600
H	1.29268200	2.14728200	0.91293200
H	1.29436200	3.81601000	0.33370500
H	-2.16576000	2.56537200	-0.57308700
H	-1.29498900	3.81591900	0.33331000
H	-1.29337100	2.14718400	0.91249100
H	-0.89007800	3.31818900	-2.65194900
H	0.89057800	3.31806600	-2.65164200
H	0.00016000	4.48139500	-1.67420100
H	0.00000100	1.35611800	-3.43787900
N	0.00000000	-1.08302600	-1.28149000
C	0.00006400	-2.51605700	-0.81379200
C	-1.26811100	-2.77048100	0.01707200
C	1.26854200	-2.77040200	0.01665200

C	-0.00015300	-3.45051300	-2.03203300
C	-0.00001000	-0.67681400	-2.60686100
H	-2.16556000	-2.56553700	-0.57234600
H	-1.29268200	-2.14728200	0.91293200
H	-1.29436200	-3.81601000	0.33370500
H	2.16576000	-2.56537200	-0.57308700
H	1.29498900	-3.81591900	0.33331000
H	1.29337100	-2.14718400	0.91249100
H	0.89007800	-3.31818900	-2.65194900
H	-0.89057800	-3.31806600	-2.65164200
H	-0.00016000	-4.48139500	-1.67420100
H	-0.00000100	-1.35611800	-3.43787900
Cl	0.00000000	0.00000000	3.60165300
Cu	0.00000000	0.00000000	1.46832900

SI*t*Bu

Energy: -542.034476 au

Sum of electronic and thermal Energies: -541.702899 au

Geometry:

C	-0.00000400	-0.56906000	-0.00000100
N	1.07882300	0.22928200	-0.12571600
C	2.47099900	-0.27512000	-0.00535600
C	2.56599900	-1.65880300	-0.66386500
C	2.85686800	-0.37906700	1.48404100
C	3.42558900	0.69017000	-0.72961300
C	0.75976200	1.66476500	0.08247300
H	2.29142500	-1.60211200	-1.72011400
H	1.89323900	-2.36671200	-0.18246500
H	3.59318100	-2.02735800	-0.58895900

H	2.78835800	0.59122900	1.98471400
H	3.88490800	-0.73649900	1.59318000
H	2.19037300	-1.07770800	1.99486200
H	3.46134000	1.67333900	-0.25328800
H	3.12893800	0.82085000	-1.77399700
H	4.43932200	0.28312000	-0.71392100
H	1.25992000	2.30515800	-0.64401300
N	-1.07882800	0.22928500	0.12571800
C	-2.47100100	-0.27512200	0.00535700
C	-2.56598700	-1.65880200	0.66387000
C	-2.85685900	-0.37907500	-1.48404300
C	-3.42560000	0.69016500	0.72960800
C	-0.75976500	1.66476800	-0.08247100
H	-2.29142800	-1.60210200	1.72012200
H	-1.89321300	-2.36670400	0.18248100
H	-3.59316400	-2.02737100	0.58895300
H	-2.78834700	0.59122300	-1.98471400
H	-3.88489600	-0.73651100	-1.59319100
H	-2.19035500	-1.07771200	-1.99485700
H	-3.46135300	1.67333400	0.25328300
H	-3.12895900	0.82084600	1.77399500
H	-4.43933100	0.28311100	0.71390800
H	-1.25992400	2.30515900	0.64401600
H	-1.05636200	1.98727300	-1.08867500
H	1.05636200	1.98726700	1.08867600

SI*t*Bu-CuCl

Energy: -2642.891053 au

Sum of electronic and thermal Energies: -2642.552165 au

Geometry:

C	-0.00001100	-0.44685000	-0.00002500
N	-1.09477900	-1.23094400	-0.10737300
C	-2.51076600	-0.74261400	-0.00375400
C	-2.76952200	0.34451900	-1.05902700
C	-2.76870500	-0.20221900	1.41494500
C	-3.46553100	-1.91559600	-0.28667600
C	-0.75075000	-2.64941800	0.12764400
H	-2.55557600	-0.03218500	-2.06224700
H	-2.16536800	1.23890200	-0.89249000
H	-3.81928400	0.64645000	-1.01886300
H	-2.57484600	-0.97013700	2.16973200
H	-3.81004700	0.11464300	1.51599500
H	-2.13050100	0.65829000	1.62601700
H	-3.38054600	-2.71391200	0.45415800
H	-3.30085400	-2.33765700	-1.28153500
H	-4.49194500	-1.54624900	-0.25018700
H	-1.28734100	-3.31729700	-0.54283200
N	1.09473400	-1.23097200	0.10734300
C	2.51074000	-0.74268200	0.00375300
C	2.76952300	0.34439700	1.05907500
C	2.76870700	-0.20223700	-1.41492000
C	3.46546700	-1.91570500	0.28662600
C	0.75066500	-2.64944300	-0.12762600
H	2.55556200	-0.03234600	2.06227800
H	2.16539200	1.23880200	0.89257300
H	3.81929300	0.64630300	1.01892900
H	2.57482500	-0.97011800	-2.16974000
H	3.81006100	0.11459100	-1.51595000

H	2.13053700	0.65830400	-1.62596500
H	3.38044600	-2.71399300	-0.45423400
H	3.30079100	-2.33779600	1.28147300
H	4.49189300	-1.54639000	0.25013800
H	1.28723800	-3.31731500	0.54287100
H	0.98261600	-2.93110400	-1.16269300
H	-0.98271100	-2.93103600	1.16272000
Cl	0.00006200	3.61380500	0.00001100
Cu	0.00002500	1.47748500	-0.00000800
